# Redefining the Genus *Corollospora* Based on Morphological and Phylogenetic Approaches

**DOI:** 10.3390/jof9080841

**Published:** 2023-08-11

**Authors:** Pedro Correia, Egídia Azevedo, Maria F. Caeiro

**Affiliations:** 1 Centro de Ecologia, Evolução e Alterações Climáticas (ce3c), Faculdade de Ciências da Universidade de Lisboa (FCUL), DBV, C2, Campo Grande, 1749-016 Lisboa, Portugal; pedro.correia451@gmail.com (P.C.); emazevedo@fc.ul.pt (E.A.); 2 Centro de Estudos do Ambiente e do Mar (CESAM Lisboa), Faculdade de Ciências da Universidade de Lisboa (FCUL), DBV, C2, Campo Grande, 1749-016 Lisboa, Portugal

**Keywords:** phylogenetic analyses, p-distance, *Corollospora maritima sensu stricto*, Halosphaeriaceae, marine fungi, ITS region

## Abstract

The present study, initially to resolve the cryptic species within *Corollospora maritima*, is to determine how to attain taxonomic discrimination at species and generic levels. Multiple sequence alignments (MSAs) of the ITS, 28S, and 18S regions of the nuclear ribosomal cistron were separately subjected to pairwise distance assessments, Bayesian, and Maximum likelihood phylogenetic analyses. Morphological descriptions of 15 type strains of *Corollospora* species, along with MSAs involving representatives of the whole genus *Corollospora* (268 isolates, many from *C. maritima sensu lato*) totaling 355 published sequences, allowed phylogenetic assessments conducted to the following p-distance thresholds in the ITS/28S regions: ≥3%/1% for species segregation and ≥8%/2% for generic segregation. This resulted in the introduction of 10 new genera encompassing 13 new combinations of current *Corollospora* species: *Ajigaurospora pseudopulchella*, *Corollosporella anglusa*, *Corollosporella ramulosa*, *Corollosporopsis portsaidica*, *Garethelia parvula*, *Honshuriella fusca*, *Keraliethelia pulchella*, *Nakagariella filiformis*, *Paracorollospora angusta*, *Paracorollospora luteola*, *Paracorollospora marina*, *Shirahamella gracilis*, and *Tokurathelia colossa*. Furthermore, seven undefined genera considered putative new genera (pNGenus A to G), and 16 undefined putative new species (seven spp. come from the resolution of the *C. maritima* complex), await re-assessment of their morphology and additional molecular data, which may result in the recognition of new taxa.

## 1. Introduction

The genus *Corollospora* was introduced by Werderman in 1922 for *Corollospora maritima*, described as a coelomycete. Barghoon and Linder [1], unknowing of the previous study, established the new genus *Peritrichospora* (Ascomycota), with *Peritrichospora integra* as the type species along with a second species *P. lacera.* Later, Kohlmeyer [2] examined the type material of *P. integra*, synonymizing it as *Corollospora maritima. Corollospora*, the largest genus among the marine fungi, belongs to the Halosphaeriaceae, Microascales, Hypocreomycetidae, Sordariomycetes, Ascomycota [3]. It comprises twenty-seven species [4,5], most of them only described as sexual morphs. However, *C. intermedia*, *C. luteola*, *C. pulchella*, and *C. anglusa* have both morphs, while *C. ramulosa*, *C. parvula*, and *C. marina* only have asexual morphs [4,6]; *Corollospora mediterranea* was introduced based on the description of chlamydospores (asexual conidia) and molecular data [4]. 

The delineation of *Corollospora* species has been mainly based on the peridial wall structure, asci and ascospores size, septation, and appendage ontogeny [7,8,9,10,11,12,13,14]. Peridium shows variations in the number of cell layers, varying from one in *C. portsaidica*, to three layers in *C. colossa*, *C. filiformis*, *C. lacera*, while most have two layers [7,12,13]. Septation of the ascospores vary from one septum in *C. anglusa*, *C. cinnamonea*, *C. gracilis*, *C. maritima*, and *C. portsaidica*, while others are phragmoseptate: *C. augusta*, *C. armoricana*, *C. beasarispora*, *C. baravispora*, *C. borealis*, *C. californica*, *C. colossa*, *C. filiformis*, *C. indica*, *C. intermedia*, *C. lacera*, *C. luteola*, *C. mesopotamica*, *C. pulchella*, *C. pseudopulchella*, and *C. quinquseptata*; *C. fusca* and *C. novofusca* have ascospores transversely and longitudinally septate. Ascospore dimensions also vary within the genus: length from 18 µm in *C. anglusa* and *C. cinnamomea*, to 220 µm in *C. fusca*; diameter from 3 µm in *C. anglusa* and *C. gracilis*, to 38 µm in *C. fusca*. Ascospores are mostly hyaline, but the following species have brown ascospores: *C. cinnamonea*, *C. portsaidica*, *C. fusca*, and *C. novofusca*. Relatively to the ontogeny of the appendages, the primary appendages (polar spines or terminal appendages) formed by an outgrowth of the episporium and mesosporium are predominantly present, although absent in *C. colossa*, *C. filiformis*, *C. pulchella*, and *C. pseudopulchella*. Equatorial and polar ribbon-shaped secondary appendages are developed by the fragmentation and peeling of the exospore, forming apical sheets or a double frill around the central septum [12,13,14,15,16]. 

Based on analyses of partial 28S sequences from the nuclear ribosomal DNA, Campbell et al. [7] carried out the first molecular study evaluating the generic placement of *Corollospora* species and other species that had been transferred from the genus *Corollospora* to *Arenariomyces*, *Kohlmeyeriella*, and *Nereiospora* [14]. Classification, based on the ontogeny of ascospore appendages as a generic character, was also evaluated [7]. These authors [7,14] stated that, despite the interspecific morphological variations in ascospores and their appendage morphology, *Corollospora* was a monophyletic genus. The fact that *Nereiospora* species grouped within the *Corollospora* clade, suggested that the absence of an exosporium and secondary exosporial appendages are not key characters for generic delineation. The study also showed that primary and secondary appendages and their distinctive ontogeny, are phylogenetically significant at the species level. *Arenariomyces* were placed in the Halosphaeriales but distantly related to *Corollospora*, while *Kolhmeyeriella tubulata* (ex *Corollospora tubulata*) was transferred to the Lulworthiales. Other studies [17] have shown that *Nereiospora* species are distantly placed from *Corollospora*. Subsequently, many studies included molecular approaches to address the taxonomy of *Corollospora* species [3,4,5,14,16,18,19,20] and other genera within the Halosphaeriaceae, which were described as being primarily marine, generally having appendaged or sheathed ascospores, with asexual morphs known for only a few genera [8]. 

*Corollospora maritima* is most frequently collected on marine woody and herbaceous substrates associated with sand grains and from sea foam [16,20,21] and plays an important environmental role as a saprobe. Interesting traits have been attributed to this fungus, such as the production of a biocompound (corollosporine) with antibacterial activity [22] and the ability to decompose hydrocarbon complexes such as petroleum [19]; it was also proposed as a bioindicator of sand beaches degradation [23]. 

*Corollospora maritima* is known as a cosmopolitan species [16,17,18,23,24,25], having been collected in Portugal, throughout multiple marine surveys [26,27,28,29,30,31]. Bebout et al. [32], based on temperature requirements for growth, reported the existence of physiological races of *C. maritima* collected at different geographical regions. Roberts et al. [27], by applying Randomly Amplified Polymorphic DNA (RAPD) analysis, also identified races in different geographical isolates of *C. maritima*. Later, it was recognized as cryptic species that may constitute a species complex [33,34,35].

More recently, the occurrence of genetic lineages of *C. maritima sensu lato* was recognized in studies mainly focused on ecological aspects involving a large number of isolates from samples collected at locations in the Gulf of Mexico, Caribbean Sea, Eastern and Western Pacific Ocean [17] and Playa Hermosa, Mexico [25]. Both studies confirmed it as a cosmopolitan cryptic species and morphologically defined by dimensions and color of ascospores; the genetic variability, evaluated through the sequencing of the ITS region from the nuclear ribosomal cistron (rDNA), clearly evidenced the existence of five lineages [17,25]. These studies reinforced the need for additional efforts to address *C. maritima* taxonomy. 

In the last decade, intensive attempts have been made at finding accurate DNA barcodes for species and higher taxa delimitation, and to establish identity thresholds [36,37,38,39,40]. The DNA regions most extensively studied were the ITS region, 28S, and 18S genes from the rDNA, and a set of protein-coding genes: *RPB2* (the second largest subunit of the RNA polimerase II), *TEF* (translation elongation factor), *RPB1* (the largest subunit of the RNA polimerase II), *B-tubulin*. Without underestimating the relevance of the others, the ITS region was proposed as the universal barcode for fungi [36]. Since then, it has been increasingly used in association with other approaches aiming at the attribution of appropriate scientific names through reference specimens [37]. The establishment of threshold values for discrimination at the different taxonomic levels is key in this matter, and an issue still waiting for answers widely accepted. This was evidenced in the following two examples of studies involving the ITS region. Velmurugan et al. [38], based on a pairwise distance (p-distance) model and 171 fungal specimens from marine sediments, suggested a barcode gap of 15% for genera, while Millberg et al. [39] proposed homologies of 98–100% for presumed species and 94–97% for genus level (study involving a *Pinus sylvestris* community). Later, Vu et al. [40] presented a comprehensive study involving ca. 100 000 fungal strains assigned to ca. 17 000 species of filamentous fungi from the CBS fungal biobank. They found the following identity values: 99.6/99.8 (ITS/28S gene) to identify fungal species and 94.3/98.2 (ITS/28S gene) to discriminate at the genus level [41]; although recognizing the need for taxonomic revisions, especially at the genus level, more than 99% of the comparisons involving the species validated in that study evidenced within-species similarities of 94–100% in the ITS region, and 96–100% in the 28S gene.

Meanwhile, implementing the idea of grouping sequences according to p-distance values, a database and sequence management environment (UNITE) [41] was created based on the ITS region of fungi (https://unite.ut.ee/, accessed on 18 December 2022). Sequences from the International Nucleotide Sequence Database Collaboration (http://www.insdc.org, accessed on 18 December 2022) are clustered in species hypothesis (SH), in steps of 0.5% p-distance value, 3% being the highest threshold value accepted for species discrimination [42]; each cluster has an SH number and DOI, to facilitate scientific communication and data assembly [43]. This platform has been increasingly used and recently was the subject of a publication [44] resuming the open discussion about how to deal with DNA-based typification, mainly involving dark fungal taxa (not cultivated in the laboratory).

Still concerning the use of molecular data in taxonomic placements, and among others considered in this work, are the publication by Jeewon and Hyde with recommendations to resolve taxonomic ambiguities [45], the study of Lücking et al. [46] debating approaches to achieve unambiguous identifications of fungi, and a more recent publication [47] with a discussion concerning the contributions from traditional taxonomic studies and challenges of metabarcoding, for novel species discovery. These studies [45,46,47] and many others, highlighted the relevance of an integrative taxonomy for species delimitation, by combining phylogeny, phenotypic and reproductive biology (when feasible). DNA barcoding using ITS region remained a first choice (mainly in metabarcoding), being advisable phylogenetic approaches based on multiple sequence alignments [45,46,47]. An in-deep approach to these subjects is found in a recent work where an integrative taxonomy was applied to species delimitation in Ascomycota [48].

The present study, including the characterization of new Portuguese isolates, was primarily concerned with the disentangling of the *Corollospora maritima* complex (*C. maritima sensu lato*), by combining molecular (p-distance assessments and Bayesian and Maximum likelihood phylogenetic analyses) and morphological evaluations. Once recognized the need of also addressing the whole diversity within the genus *Corollospora*, the evaluations were extended to the *Corollospora* species with published sequences, ultimately conducting generic revision. A stepwise assessment of p-distances through multiple sequence alignments (MSAs) involving the ITS region and the 28S gene, was then carried out: (i) initial evaluation of the *C. maritima* complex, to identify the most likely representatives of *C. maritima sensu stricto*, (ii) sectorial evaluations targeting the genetic diversity within each *Corollospora* species, and (iii) global evaluation, based on representatives of all *Corollospora* species, to find genetic relationships between them. These assessments are anticipated to find out thresholds for species and generic delineation. The phylogenetic analyses were then extended to concatenated MSAs including the three ribosomal regions, and extra sequences from other Halosphaeriaceae, other genera within the Microascales, and representatives of the Xylariales (outgroup). Finally, the molecular results were confronted with the morphological descriptions of *Corollospora* species, allowing to establish new genera and new combinations of recognized species.

## 2. Materials and Methods

### 2.1. Collection Sites and Collected Materials

The present study includes a total of eleven isolates of *Corollospora maritima*: nine isolates resulted from a survey conducted in 2007–2008, where *Pinus pinaster* and *Fagus sylvatica* baits were submerged in two marinas (Cascais and Sesimbra) in the Portuguese west coast, as described in Azevedo et al. [28,29], and two new isolates were sampled on drift substrates and sand collected in September 2019, in Conceição beach (38°42′ N, 9°25′ W), Cascais (Table 1). These isolates belong to the collection of marine fungi from Faculdade de Ciências da Universidade de Lisboa (FCUL).

The collections were carried out in the intertidal zone during low tide. Sea water temperature, pH, and seawater salinity were recorded, by means of a thermometer, commercial pH measuring strips (Merck), and a refractometer Atago S/Mill-E (Fisher Scientific Bioblock).

### 2.2. Incubation of Substrates and Fungi Detection

The conditions of incubation and fungal detection concerning the survey of 2007–2008, were described in Azevedo et al. [28,29]. The small pieces of drift substrates collected in 2019 were incubated with sand samples (30 g/dish) in Petri dishes for three months, having been re-hydrated with sterile seawater and examined under a dissecting microscope Leica DM 750 (Leica Microsystems CMS GmbH, Wetzlar, Germany), on a weekly basis, for the detection of fruit bodies and spores of marine fungi. 

### 2.3. Morphological Characterizations and Establishment of Corollospora maritima Isolates

The methodologies referred to in this section were described by Azevedo et al. [28,29]. Briefly, fruit bodies detected under a dissecting microscope Wild M8 (Germany) were picked up with a needle and observed under a light microscope (Leitz Larboux S with Normaski optics) using seawater as mounting media. The micromorphology of reproductive structures was recorded and identified with the aid of the following illustrated dichotomous keys [16,21,49,50]. Photographs of microscopic characters were taken with a Leica Wild MPS camera using Fujichrome RTP 135 64T Tungsten film.

Axenic cultures of the new isolates were obtained by single spore isolation on corn meal agar (CMA) (Oxoid) prepared in 50% seawater supplemented with agar (LABCHEM) to a final concentration of 17 g/L, and 0.05 mg/L chloramphenicol. The isolates were preserved at 4 °C in sterile 50% sea water. The macro-morphology of fungal cultures was characterized after three weeks of incubation at 25 °C on CMA, following the method described by Sidrim and Moreira [51]. Photographs and micromorphological characterization of the colonies mounted in lactophenol were taken with a smartphone (Samsung Galaxy J5) camera.

### 2.4. Molecular Evaluation

#### 2.4.1. DNA Extraction

DNA extraction was based on the in-house method described by Azevedo et al. [52]. Briefly, the mycelium or other fungal structures were incubated at 60 °C for 16 h with 0.5 mg/mL proteinase K (Bioline) in 100 mM Tris-HCL pH 8.9. The next steps were an incubation at 98 °C for five minutes (proteinase K inactivation), followed by centrifugation for ten-minutes at 12,000× *g*; the supernatant containing the extracted DNA was maintained at 4 °C (−20 °C for long-term storage). 

#### 2.4.2. PCR Amplification, Sequencing, and Processing the Sequencing Results

The nuclear ribosomal ITS region (ITS1+ITS2+5.8S gene) was amplified by the primers ITS4/ITS5 [53]. The nuclear ribosomal 28S gene was partially amplified by the primers LR0R/LR5 [54]. PCR reaction mixtures of 20 µL contained 10 µL of My Taq^TM^ Red Mix 2x (Bioline), 2 µL of 10 µM forward and reverse primers (1 µL/each), and template DNA (1 µL), diluted in ultrapure H_2_O. The amplifications occurred in a Thermocycler T100 (BioRad) for 35 cycles with an initial denaturation step of three minutes at 95 °C, followed by 35 amplification cycles and a final extension at 72 °C for three minutes. Each amplification cycle consisted of denaturation at 95 °C for 15 s, annealing at 48 °C/52 °C (28S gene/ITS region) for 15 s, and extension at 72 °C for 30 s. Aliquots of the PCR products were visualized on 1% agarose gels in TBE (Tris-borate EDTA buffer) stained with ethidium bromide (0.2 mg/mL); and the PCR products with the expected bands were purified with the DNA Clean & Concentrator-5 kit (Zymo Research), according to the manufacturer’ instructions, and sent for Sanger sequencing in a commercial lab (StabVida, Portugal).

The sequences were analyzed and manually edited with the aid of the program BioEdit v7.2.5 [55], by visualization of the chromatograms. Then, the sequences were identified using the Basic Local Alignment Search Tool (BLAST), available at the National Center for Biotechnology Information (NCBI) website (https://www.ncbi.nlm.nih.gov/). 

#### 2.4.3. Phylogenetic Analyses and Assessment of Pairwise Distances

ITS and 28S evaluations were performed with the sequences of the 11 Portuguese isolates selected for this study (Table 1) and sequences (also 18S) retrieved from the following databases: NCBI (https://www.ncbi.nlm.nih.gov/nuccore, NITE (https://www.nite.go.jp/nbrc/catalogue/), and UNITE (https://unite.ut.ee/) (Table S1). The molecular evaluations, separately addressing each ribosomal region, included the publicly available sequences from *Corollospora maritima* and sequences from the other *Corollospora* species. The global evaluations also included outgroup sequences, i.e., from *Cucullosporella mangrovei* and *Trailia ascophylli*.

The sequences were subjected to multi-sequence alignments (MSAs) using MUSCLE software in MEGA X 10.1 [56], with the elimination of the gaps present in more than 50% of the sequences. The distance matrices were generated with the use of the software MEGA X 10.1 (https://www.megasoftware.net/), using the p-distance model and 1000 bootstraps, including transitions and substitutions with uniform ratios between sites as variance parameters and pairwise deletion as a gap processing parameter. The estimation of evolutionary divergence between sequences was conducted in MEGA X 10.1.

The phylogenetic analyses followed the Maximum likelihood (ML) model using MEGA X 10.1, and the Bayesian Posterior Probabilities (PP) model using BEAST 1.10.4 [57] and associated programs (BEAUTI 1.10.4, TreeAnnotator 1.10.4 and FigTree 1.4.4.). The ML tree was built using as variance parameters 1000 bootstraps, the Tamura–Nei model for nucleotide substitutions, and uniform ratios between sites. The BEAST 1.10.4-based tree was built using BEAUTI and basing the tree on the Yule speciation method; the .xml file was then imputed into BEAST to generate a file with ten thousand replicas; this output file was analyzed on TreeAnnotator, and a final version of the tree was obtained using FigTree. A combined tree was the final output, based on the tree with the most fitting support values: posterior probability (PP) or bootstrap (BS). 

A concatenated/multi-loci sequence alignment was produced based on MSAs for the three ribosomal regions (28S, ITS, and 18S) extended to sequences representative of other Halosphaeriaceae, other genera belonging to the same order (Microascales) and to the Xylariales as outgroup. Concerning the Halosphaeriaceae, this MSA included two representative strains of each species with ITS sequences without disputable p-distances in the ITS and 28S regions. All sequences present in the former alignments were kept. MEGA X was used to concatenate partial MSAs into a final wide-ranging MSA. The phylogenetic analyses (ML and PP) and the final tree followed the methodologies referred to above.

#### 2.4.4. Steps of the Molecular Evaluation

Preliminary studies comprised the identification of the sequences generated in this study and others retrieved from the databases (when required), using the Nucleotide Basic Local Alignment Search Tool (BLASTN) (https://blast.ncbi.nlm.nih.gov/). These evaluations contributed to the construction and annotation of a comprehensive table (Table S1), as is detailed in the results section.

The first MSA targeted the placement of the *Corollospora maritima* complex (*C. maritima sensu lato*) within the genus *Corollospora*. Estimation of p-distance values in the ITS region included all the available sequences of *C. maritima* and representative sequences (two, whenever possible) of the other *Corollospora* species. The expected result was the identification of the strains/isolates belonging to *C. maritima sensu stricto*.

The next assessments consisted of sectorial MSAs, each one only including the public sequences of one *Corollospora* species and two representative sequences of *C. maritima* (resulted from step 1). This process aimed at the selection of representative sequences of each taxon, to participate in global evaluations targeting the genus *Corollospora*. 

The global assessments, also based on MSAs separately targeting the ITS and 28S regions, intended to find out molecular and phylogenetic relationships among the current *Corollospora* species.

Finally, a multi-loci assessment extended to other representatives of the Microascales and to outgroup sequences of Xylariales, intended to confirm the former results.

### 2.5. Description of New Genera and New Combinations

Delineation of new taxa (new genera and new combinations based on type strains of previously recognized species) was based on the molecular data and morphological descriptions from the type strains of the re-assessed species. 

## 3. Results and Discussion

### 3.1. New Samplings

The new samplings were carried out at Conceição beach (Cascais), where the sea water temperature was 16 °C, the salinity was 36 and the pH was 6.5.

From the attempts of single spore isolation, two new isolates of *Corollospora maritima* were obtained from ascocarps on a drift culm and sand (CaC1 and CaC2, respectively) (Table 1).

### 3.2. Characterization and Identification of the Portuguese Isolates

The morphological characterization of the Portuguese isolates of *C. martima* ([28,29]; present work) is shown in Table 1 and further described in Section 4.1.

The molecular identifications were carried out with the DNA extracted from 11 isolates, amplified in PCR reactions targeting the ITS and/or 28S regions (GenBank sequence IDs in Table 1). These sequences were subjected to BLASTN, and the highest hits revealed a high number of sequences registered as *C. maritima* (at least 80 ITS and ten 28S sequences), with the following identity values: ITS sequences: >98%, 28S sequences: >99%. 

### 3.3. Is Corollospora maritima a Species Complex?

Preliminary estimation of p-distance values was based on an MSA with only 285 positions, involving 137 ITS sequences (all available sequences of *C. maritima* and up to two sequences from each *Corollospora* species). Despite the expected high p-distance values found (>10%) between many *C. maritima* sequences, they grouped in three sets, each consisting of sequences set apart by p-distances ≤4%. Two groups only included *C. maritima* sequences and the third, with more than 40 *C. maritima* sequences, also included two sequences of *C. portsaidica*.

An assessment targeting the ITS region of the *C. maritima* complex, but also including *C. portsaidica* sequences, resulted in an MSA with 451 positions and 173 sequences (nine from the Portuguese isolates; Table 1), distributed in three groups (hereinafter referred to as Group 1, Group 2, and Group 3), and one single sequence. Each group consisted of sequences segregated by p-distances of 0–6%: Group 1 comprised 104 isolates (nine Portuguese) of *C. maritima*, Group 2 consisted of 58 *C. maritima* and two *C. portsaidica* isolates, and Group 3 consisted of 11 *C. maritima* isolates (Table 2). The single sequence, from the strain NBRC 106651, was identified as *C. maritima* by BLASTN (Table 2, Tables S1 and S2).

### 3.4. Sectorial Molecular Evaluations 

File S1 gathers the MSAs separately targeting each of the groups previously revealed in the *Corollospora maritima* complex, as well as the MSAs generated for the other species of *Corollospora* (addressed in Section 3.5.3). 

File S2 shows the p-distance matrices from the previous MSAs, which are summarized in Table 2; notes to Table 2 (and to Table S1) are provided in Table S2.

#### 3.4.1. Disentangling the *Corollospora maritima* Complex

The MSAs generated for the ITS and 28S regions from the three groups of the *C. maritima* complex, also included the strain NBRC 106651, stated with the following: “DNA sequence analysis suggested the identification of this strain was disputable” (https://www.nite.go.jp/nbrc/catalogue/NBRCCatalogueDetailServlet?ID=NBRC&CAT=00106651&lang=jp, accessed on 30 July 2023).

Group 1 encompasses 104 isolates, 11 (~10%) described in this study and 71 (~67%) belonging to the lineage 1 established in Velez et al. [17,25]; of which 104 isolates are represented by ITS sequences and only 21 by 28S sequences (Table 1 and Table 2, Table S1). Little genetic diversity was found in the ITS region of 103 isolates (p-distances ≤ 3%, even 0–1% for 101), except between them and the isolate CMG 52 (p-distances of 3–6%) (File S2). For the 21 isolates with 28S sequences (including CMG 52), they segregate by p-distances of 0% (1% for the isolates MD 825 and JK 4834, without ITS sequences and, therefore, not subjected to the global evaluations) (File S2; Table S1). For the strain NBRC 106651, p-distances of 13–15%/6–7%, in the ITS/28S regions were recorded, clearly above the expected values for isolates from the same species (File S2; Table 2). The main conclusion from these assessments is that (except for the isolate CMG 52) all the isolates included in Group 1 appear to belong to the same species, and therefore referred to as *C. maritima sensu stricto* (see Section 3.5.3). Therefore, in further evaluations, two members of Group 1 were used as representatives of both *C. maritima* and genus *Corollospora*.

Group 2 consists of 58 *C. maritima* and three *C. portsaidica* isolates (only one with sequences from both regions). Sixty isolates have ITS sequences: two *C. portsaidica* and 58 *C. maritima* (strain NBRC 32118 and 57 isolates belonging to the previously named lineages 4 and 5) [17,25] (Table 2 and Table S1). This group splits into four sub-groups (A–D) segregating by p-distances of 1–6% from each other. Each sub-group consists of the following sequences segregating by p-distances of 0% (0–3% in the sub-group D): sub-group A, two sequences of *C. portsaidica*; sub-group B, 50 sequences from the lineage 5 [17,25]; sub-group C, five sequences from the lineage 4 [17,25]; sub-group D, three sequences of *C. maritima* (strain NBRC 32118, with disputable identification, according to its voucher in NBRC) and two isolates included in the lineage 5 [17,25]. For the 28S gene, Group 2 is represented by only three isolates segregating by a p-distance of 0%: *C. portsaidica* MF 832 and MD 1301 (former sub-group A), and *C. maritima* NBRC 32118 (former sub-group D) (File S2). The members of Group 2 evidence p-distances of 7–9%/3% in the ITS/28S regions, from the strain NBRC 106651; and 15–18%/7% in the ITS/28S regions, to the selected representatives of Group 1. These values are clearly above the p-distances expected between representatives of the same species (File S2; Table 2).

Group 3 is exclusively represented by ITS sequences of 11 *C. maritima* isolates previously named lineages 2 and 3 [17,25] (Table 2 and Table S1). These isolates split into two sub-groups (two and nine sequences, corresponding to the lineages 2 and 3, respectively) segregating by a p-distance of 4% (0% between the sequences of each sub-group) (File S2). High p-distances (16–17%) were found, either to the strain NBRC 106651 or to the representatives of Group 1 included in the evaluation; once again, values clearly above those expected for representatives of the same species (File S2; Table 2).

A query to UNITE considering distances of 3.0 to the closest SH [42] fully supports the aforementioned results: Group 1 is represented by SH0176164.09FU (96 sequences) and SH0176168.09FU (exclusively the isolate CMG 52); Group 2 splits in four SHs (SH0053802.09FU, SH0053803.09FU, SH0053804.09FU, SH0053805.09FU), and Group 3 in two: SH0158721.09FU (nine sequences) and SH0158722.09FU (two sequences); the ITS sequence of the isolate NBRC 106651, like other sequences only represented in NBRC, was not found.

#### 3.4.2. Molecular Diversity within Each *Corollospora* Species 

Estimation of p-distance ranges between the members of each species, allowed assessment of genetic diversity and to select representatives (the most distant strains within each species) for the global alignments (restricted to representative sequences) to analyze the relationships between the species of the genus *Corollospora*.

A highly variable number of members with published sequences (2–60) was found in each of the 17 *Corollospora* species subject to this study (Table 2). Eleven species (>50%) had excluded sequences (28 in total), resulting in the elimination of 12 isolates/strains (out of 309) (Table 2, Tables S1 and S2). The eliminations were due to different issues, namely identification problems (identity <90% with any other published sequence), mistaken identifications, and/or incorrect sequence submissions as, for example: one sequence of *Alternaria* spp. registered as *C. anglusa* and three identical 18S sequences (sharing 100% coverage and 1094/1094 identities), attributed to isolates from three species (*C. pulchella* NBRC 32124, *C. fusca* NBRC 32108, and *C. colossa* NBRC 32103) (Tables S1 and S2). 

Regarding the p-distances found within each species, the split into two or more clusters with discontinuity of values was occasionally found. When these values were clearly above the thresholds expected for species segregation (3% in the ITS region and 1% in the 28S gene), each cluster may represent a different species. When the molecular identification is not fully supported, that cluster will represent a putative new species. The absence of full support means that the molecular data are not coming from a type strain, and there is insufficient morphological information or a lack of data from the ITS region.

#### 3.4.3. New Taxa and Inconclusive Assignments 

Concerning the *Corollospora maritima* complex, it is worth noticing the absence of a type of strain. Therefore, type sequences are not available. In total accordance with the lineages previously established [17,25], the three groups formerly outlined in Section 3.4.1 clearly segregated from each other by p-distances undoubtedly above the thresholds accepted for species segregation. Three species thus seem to correspond to these genetically well-defined groups. Group 2 is heterogenous, also including an already well-established species (*C. portsaidica*) and Group 3 only comprehends 11 isolates circumscribed to two geographical regions (Hawaii and Cuba). Group 1 exclusively encompasses morphologically recognized *C. maritima* members, in large numbers and worldwide in distribution; therefore, this group is considered the most reliable representative of *Corollospora maritima sensu stricto,* from now designated *C. maritima*.

P-distances over the aforementioned thresholds were also found within other species evaluated in this study: *C. colossa*, *C. fusca*, and *C. intermedia*/*Varicosporina prolifera* (in the ITS and 28S regions); *C. gracilis* (in the ITS region); *C lacera*, *C. pulchella*/*Clavariopsis bulbosa*, *C. pseudopulchela*, and *C. quinqueseptata* (in the 28S gene); (Table 2 and Table S2; File S2).

A query to UNITE [42] also confirmed two SHs for *C. gracilis* (SH0182673.09FU, SH0182674.09FU) and *C. fusca* (SH0099123.09FU, SH0137920.09FU).

It is accepted that the re-evaluation of taxonomic placements (creation of new taxa and/or new combinations) must depend on data from newly evaluated strains and/or attributed to recognized type strains [37]. Therefore, except for the Portuguese isolates of *C. maritima*, this study was reliant almost exclusively on published data (without the inclusion of new morphological and/or molecular data). However, many sequences belong to the type strain of the recorded species. 

Thus allows combining of the published molecular and morphological data for the establishment of new taxa. When a new taxon is based on molecular data not derived from type material or on insufficient molecular data (usually given by the ITS region), it was considered a putative new taxon.

This study also identified problematic sequences, which led to “inconclusive assignments”, and strongly recommends the re-evaluation of the respective species. *C. intermedia*/*Varicosporina prolifera* is a paradigmatic case because there is no type of strain of *Corollospora intermedia* and the anamorph *Varicosporina prolifera* NBRC 100413, recognized by Nakagiri [58], lacks published sequences. On the other hand, the sequences of the other two strains of this species (*C. intermedia* NBRC 104402 and *Varicosporina prolifera* NBRC 32120) segregated by p-distances far from any threshold acceptable for representatives of the same species (14%/4% in the ITS/28S regions), and another 28S sequence (from *C. intermedia* PP3910) is still more distant (≥12%) from the others (File S2; Table 2, Tables S1 and S2); see also Table 3.

Finally, apart from the exceptions indicated, the p-distances between most of the members of the species evaluated were, as expected, clearly below the maximum thresholds for species segregation assumed in this study (3% in the ITS region, 1% in the 28S gene).

### 3.5. How Many Taxa May Be Defined?

In addition to the finding of distinct species in the *C. maritima* complex and within other *Corollospora* species, particularly high p-distances (≥15%/≥6% in the ITS/28S region) were also found between *C. maritima* and the representatives of many other species (Table 2; File S2). Many of those values are even compatible with generic segregation [39,40,59].

To address the hypothesis that *Corollospora* may consist of a set of genera, global assessments based on suitable representatives of the current *Corollospora* species, were performed. Whenever possible, the global MSAs included two sequences of each taxon, selected in accordance with the following criteria: (i) sequence length (at least 450 nt/500 nt, for the ITS/28S regions), (ii) representativeness (belonging to the type of strain), and (iii) coverage (belonging to a strain with available ITS and 28S sequences, by this order of relevance). Furthermore, to represent the genetic diversity already identified in each taxon (Table 2), the more distant sequences were selected and taxa with greater diversity were represented by additional sequences.

**Figure 1:**
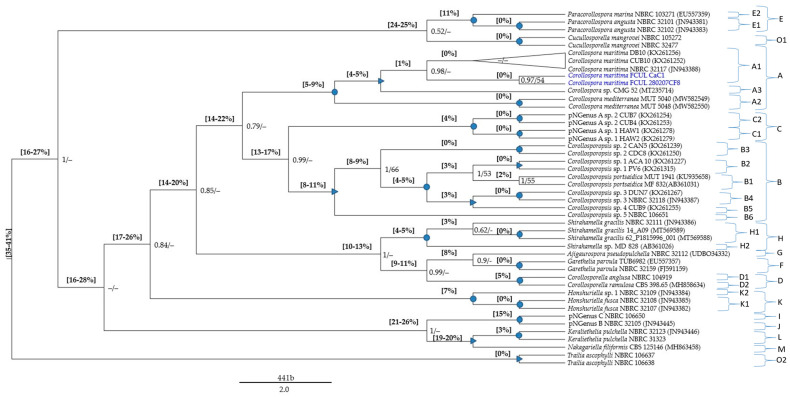
Phylogenetic tree based on new and retrieved nuclear rDNA ITS sequences representative of Halosphaeriaceae taxa assembled in a 441 positions MSA. Posterior probability (PP) values from the Bayesian analysis followed by bootstrap (BS) values from the maximum likelihood analysis are added to the right of the nodes (PP/BS). Numbers inside square brackets to the left of the nodes represent the minimum and maximum p-distance values between the sequences clustering in the two splitting branches. Circles signalize highly supported nodes (PP = 1; BS ≥ 90), and triangles signalize well-supported nodes (PP ≥ 0.95; BS ≥ 70); values of PP < 0.5 and Bs < 50% are signalized by “-”. New sequences are written in blue, and all sequences are labeled with the strain name and accession number. A—species of the genus *Corollospora*; B to M—species belonging to the new genera defined in the present study: *Corollosporopsis* (B), pNGenus A (C), *Corollosporella* (D), *Paracorollospora* (E), *Garethelia* (F), *Ajigaurospora* (G), *Shirahamella* (H), pNGenus C (I), pNGenus B (J), *Honshuriella* (K), *Keraliethelia* (L), *Nakagariella* (M); Outgroups—O. Current species comprising the putative new genera (Table 3): pNGenus A: *C. maritima* (lineages 2 + 3 in [18,25]); pNGenus B: *C. colossa*, pNGenus C: *C. colossa*.

**Figure 2:**
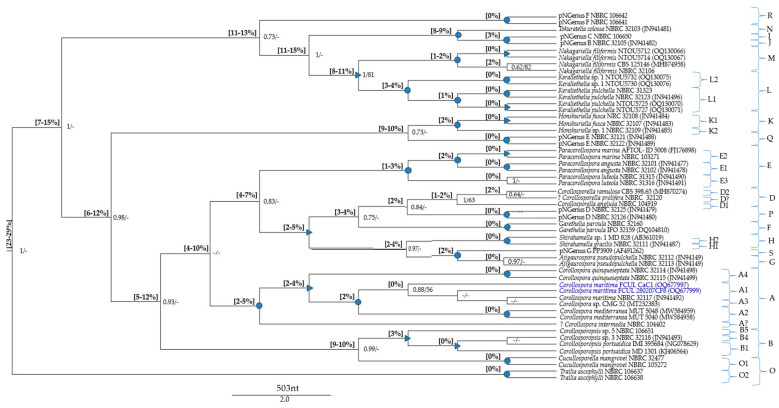
Phylogenetic tree based on new and retrieved nuclear rDNA 28S sequences representative of Halosphaeriaceae taxa assembled in a 503 positions MSA. Posterior probability (PP) values from the Bayesian analysis followed by bootstrap (BS) values from the maximum likelihood analysis are added to the right of the nodes (PP/BS). Numbers inside square brackets to the left of the nodes represent the minimum and maximum p-distance values between the sequences clustering in the two splitting branches. Circles signalize highly supported nodes (PP = 1; BS ≥ 90), and triangles signalize well-supported nodes (PP ≥ 0.95; BS ≥ 70); values of PP < 0.5 and Bs < 50% are signalized by “-”. New sequences are written in blue, and all sequences are labeled with the strain name and accession number. A—species of the genus *Corollospora*; B to S—species belonging to the new genera defined in the present study: *Corollosporopsis* (B), *Corollosporella* (D), *Paracorollospora* (E), *Garethelia* (F), *Ajigaurospora* (G), *Shirahamella* (H), pNGenus C (I), pNGenus B (J), *Honshuriella* (K), *Keraliethelia* (L), *Nakagariella* (M), *Tokurathelia* (N), pNGenus D (P), pNGenus E (Q), pNGenus F (R), pNGenus G (S); outgroup—O. Current species comprising the putative new genera (Table 3): pNGenus B: *C. colossa*; pNGenus C: *C. colossa;* pNGenus D: *C. cinnamomea;* pNGenus E: *C. lacera*; pNGenus F: *C. pseudopulchella* (NBRC 106641 + NBRC 106642); pNGenus G: C. *quinqueseptata* PP3909.

**Figure 3:**
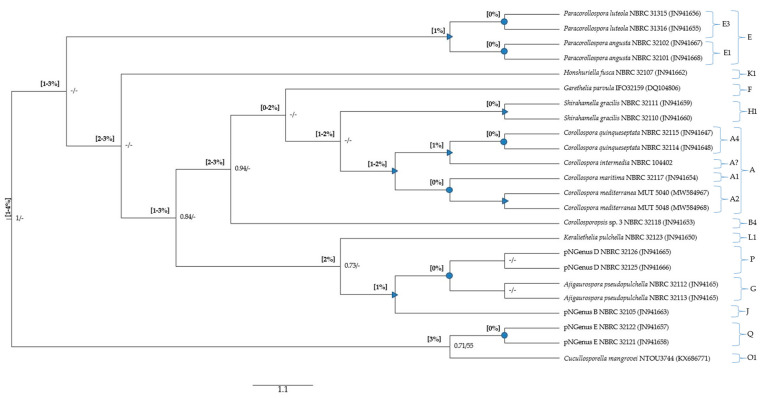
Phylogenetic tree based on new and retrieved nuclear rDNA 18S sequences representative of Halosphaeriaceae taxa assembled in 839 positions MSA. Posterior probability (PP) values from the Bayesian analysis followed by bootstrap (BS) values from the maximum likelihood analysis are added to the right of the nodes (PP/BS). Numbers inside square brackets to the left of the nodes represent the minimum and maximum p-distance values between the sequences clustering in the two splitting branches. Circles signalize highly supported nodes (PP = 1; BS ≥ 90), and triangles signalize well-supported nodes (PP ≥ 0.95; BS ≥ 70); values of PP < 0.5 and Bs < 50% are signalized by “-”. New sequences are written in blue, and all sequences are labeled with the strain name and accession number. A—species of the genus *Corollospora*; B to L—species belonging to the new genera defined in the present study: *Corollosporopsis* (B); *Paracorollospora* (E); *Garethelia* (F); *Ajigaurospora* (G); *Shirahamella* (H); pNGenus B (J); *Honshuriella* (K); *Keraliethelia* (L); pNGenus D (P); pNGenus E (Q); O—outgroup. Current species comprising the putative new genera (Table 3): pNGenus B: *C. colossa;* pNGenus D: *C. cinnamomea;* pNGenus E: *C. lacera*.

The global MSAs (File S3) were subjected to pairwise distance estimations (File S4) and phylogenetic analyses; p-distance ranges were also indicated in the phylogenetic trees (Figure 1, Figure 2 and Figure 3).

#### 3.5.1. How Many Species?

The global assessments based on MSAs including representatives of all *Corollospora* species with sequence data, confirmed the p-distances stated in the previous sections and summarized in Table 2. Thus, in addition to confirmation of the species under analysis, 13 members (from diverse species) were deemed unidentified species, i.e., putative new species (pNSpecies). 

The pNSpecies [explanations and placements in the phylogenetic trees (Figure 1 and Figure 2), provided in the Section 3.5.2] are members from several *Corollospora* species: strain CMG 52 (clade A4), strain MD 828 (clade D6), strain NBRC 32109 (clade F2), strains NTOU5730 and NTOU5732 (clade L2), strains NBRC 106650 and NBRC 32105 (clade I/J), 11 isolates (Group 3 in Section 3.4.1) segregating in two species (clades C1, C2), 59 isolates (part of Group 2 in Section 3.4.1) segregating in five species (clades B2 to B6) (see also Table S1).

#### 3.5.2. How Many Genera?

Regarding the p-distances between *C. maritima* and the other species under analysis, only two species (both represented by type strains) could undoubtedly be recognized as belonging to the genus *Corollospora*: *C. mediterranea* and *C. quinqueseptata* (excluding the isolate PP3909, segregating by a p-distance of 8% from the other two *C. quinqueseptata* and *C. maritima*). It is worth noticing that the maximum p-distance between these two species and *C. maritima* is 6% in the ITS region and 2% in the 28S gene (Table 2 and Table 3; File S4), close to the thresholds recognized for generic segregation (40): 5.7% (assuming 94.3% identity) in the ITS region and 1.8% (assuming 98.2% identity) in the 28S gene. 

Therefore, from the results of the global assessments only targeting the *Corollospora* species with available molecular data (19 out 27), only the three species just mentioned appear to belong to the genus *Corollospora*. The other species may become new genera to be introduced, their number depending on the phylogenetic relationships (p-distances included) to be found between them (File S4; Table 3), and the morphological features described for the corresponding type strains (Table 4).

To better address the question of expected p-distances between species from different genera within the Halosphaeriaceae, the final analyses included outgroup sequences from species of other recognized halosphaeriacean genera (*Cucullosporella mangrovei* and *Trailia ascophyli*).

The minimum p-distance found between *Corollospora* and outgroup sequences was 21%/8% in the ITS/28S regions, for *Cucullosporella mangrovei*; higher values (32%/19%) were found for *Trailia ascophyli*, which demonstrates a wide range of p-distances among species belonging to the Halosphaeriaceae. Accordingly, only *Trailia ascophyli* was segregated from the other species with partially high support values (PP = 1; BS < 50) in both phylogenetic trees (Figure 1 and Figure 2; File S4). Additionally, the range of p-distances found between the *Corollospora* species and the outgroups were 21–41% in the ITS region, 8−23% in the 28S gene, and only 3–4% in the 18S gene (Figure 1, Figure 2 and Figure 3, File S4). These results are in accordance with previous studies regarding p-distance assessments at species and genera levels, which pointed to a higher resolution of the ITS region [36,40].

Data from the ITS was then considered required for the establishment of new genera, contributing the 28S gene for a second level of validation. The 18S gene was included to allow comparison of threshold values and evaluation of suitability in phylogenetic analyses aiming at species identification, frequently performed with the inclusion of 18S sequences, namely in concatenated MSAs and coalescent analyses [8,20,48].

The data concerning the ITS region clearly evidenced six species segregating from the others (*C. maritima* included) by minimum values of p-distance of at least 16%, clearly above the values presumed for genus segregation [38,39,40]: *C. colossa*, *C. filiformis*, *C. fusca*, *C. angusta*, *C. marina*, and *C. pulchella*/*Clavariopsis bulbosa* (p-distance = 3% between the two morphs of this species).

Considering the 28S gene, a minimum p-distance of 4% (mostly 6%) was found between the members of the taxa referred to above. Additionally, a p-distance of 8% to the others (12% to *C. maritima*) was evidenced by the type of strain of *C. colossa* (NBRC 32103), which lacks ITS sequence (File S4; Table 3; see also Table S1).

The taxa referred above, each one consisting of one or more strains, also evidence distinctive morphological features (Table 4), which contributed to their proposal as new genera.

Each new genus consists of one or more species (new combinations), whose descriptions are provided in Section 4.2 (Figure 1 and Figure 2; Table 4 and Table S1; File S4). The new taxa are: *Paracorollospora* (*Paracorollospora angusta*, *Paracorollospora marina*, and *Paracorollospora luteola*) (clade E); *Honshuriella* (*Honshuriella fusca* and *Honshuriella* sp.) (clade F); *Nakagariella* (*Nakagiriella filiformis*) (clade G); *Keraliethelia* (*Keraliethelia pulchella*) (clade H); *Tokurathelia* (*Tokurathelia colossa*) (clade N), and the putative new genera B and C, each with one species (strains NBRC 32105 and NBRC 106650, respectively) (clade I/J). Each new genera segregate in highly supported clades (PP = 1; BS > 90) in both phylogenetic trees (Figure 1 and Figure 2).

The new genus *Tokurathelia* was exceptionally created despite missing an ITS sequence. This was due to be based on a type of strain morphologically distinct from the other *Corollospora* species (Table 4), with a 28S sequence validated by the Fungal Barcoding Consortium (FBC), segregating by high p-distances (≥8%) from the other species under evaluation (Table 3). 

Additionally, there are other species with distinctive morphological features, which segregate from *C. maritima* by p-distances (14–17%/5% in the ITS/28S regions) also accepted for generic segregation [39,40]. These new genera and the new combinations listed below are described in Section 4.2: *Corollosporopsis* (*Corollosporopsis portsaidica* and five *Corollosporopsis* spp.) (clade B); *Shirahamella* (*Shirahamella gracilis* and *Shirahamella* sp.) (Clade H); *Corollosporella* (*Corollosporella anglusa* and *Corollosporella ramulosa*) (Clade D); *Ajigaurospora* (*Ajigaurospora pseudopulchella*) (Clade G); *Garethelia* (*Garethelia parvula*) (clade F); pNGenus A (two spp. corresponding to the two sub-groups of Group 3 defined in Section 3.4.1) (clade C) (See Table 3 and Table 4; Figure 1 and Figure 2; Files S2 and S4).

In the ITS region, *Corollosporopsis* and the pNGenus A, both segregate by a minimum p-distance of 13% from the other species and 14% from *C. maritima*. Identical values were found for the four new genera first mentioned, except between their species, segregating from each other by lower p-distances: 8–10%/2–3% in the ITS/28S regions (Table 3; File S4). Considering that these values are just slightly above the minimum threshold accepted for genus segregation (40), these taxa could either be accepted as four new genera or could be proposed as species belonging to the same genus. The first option (four separate genera) was chosen due to the obvious morphological differences among them (Table 4 and descriptions in Section 4.2).

The species of these six new genera (except *Ajigaurospora*, with only one ITS sequence) cluster in highly supported clades (PP = 1; BS > 90); they also segregate from the closest genus/genera with well-supported PP values (>0.9), but only in the ITS region (Figure 1 and Figure 2), which is certainly due to the previously mentioned higher resolution of this region for discrimination at species and genus levels.

There are three putative new genera (A, B, and C) recognized in the present study that are only supported by molecular data not attributed to a type of strain. The pNGenus A comprises two species corresponding to the lineages 2 and 3 of *C. maritima sensu lato* [17,25], which lack morphological descriptions and designation of type strains. It is notable that each species is from one distinct geographical region (Hawaii and Cuba), the “cubensis” species co-occurring with *Corollosporopsis* sp. 4. 

Concerning the species of pNGenus B and pNGenus C (strains NBRC 32105 and NBRC 106650, respectively), which current taxonomic placement is *C. colossa*, they should be placed in the new genus *Tokurathelia*. However, this would be in total disagreement with the p-distance ≥8% in the 28S gene, evidenced by the type of strain of the new genus *Tokurathelia* (missing ITS sequence). Notice that these two putative new genera were based on ITS sequences (p-distance of 15% between both), the 28S sequences being decisive for the establishment of a clear segregation from the new genus *Tokurathelia.* On the other end, the three genera cluster in a highly supported clade (PP = 1; BS > 90), where the two pNGenera group in a highly supported sub-clade (I/J) (Figure 2). These results confirm the need for data from the ITS region to get a better resolution in taxonomic placements and recommend the morphological and molecular re-evaluation of these isolates; new samplings would be very helpful. Apart from other possible explanations, including the existence of intragenic variation in the ribosomal region [59,60], the current *C. colossa* may be another cryptic taxon.

Still concerning the intragenic variation found in many fungal species, it is often under the 3% divergence threshold in the ITS region (p-distance = 0.0083–0.0268) [60]. However, it may explain several pNSpecies and other two pNGenera found in the present study: *Corollospora* sp. CMG 52, *Shirahamella* sp., *Keraliethelia* sp. 1, and the species from the pNgenera F and G are discussed in the next Section 3.5.3.

#### 3.5.3. Undetermined Taxonomic Placements

Considering the existence of data for the ITS region and the undoubtedly high p-distances (14–24%) to *C. maritima*, three putative new genera (A, B, and C) not based on type strains, were defined in the previous section (Table 4). On the other end, though only relying on sequences from the 28S gene evidencing p-distances ≥5% to *C. maritima*, there are other species that may not belong to the genus *Corollospora* (Table 3).

Therefore, four putative new genera (pNGenus D to pNGenus G) were found in the global evaluations involving the 28S gene (p-distance: ≥6% to *C. maritima*; ≥2% to the other species). They are based on isolates currently identified as *C. cinnamomea* (pNGenus D), *C. lacera* (excluding the strain PP2509) (pNGenus E), *C. pseudopulchella* (only the strains NBRC 106641 and NBRC 106642) (pNGenus F), and *C. quinqueseptata* PP3909 (pNGenus G) (Table 3; File S4). Each one of these putative new genera appear in highly supported clades (PP = 1; BS > 90) in the phylogenetic tree, the pNGenus G clustering with the new genus *Ajigaurospora* (PP = 1; BS = 98) (p-distance = 2%) (Figure 2; Table 3).

Furthermore, *Paracorollospora luteola* could be accepted as a new combination, only based on 28S sequences from the type of strain (*C. luteola* TKB-C-1250 = NBRC 31315) and other two isolates, which cluster in the new genus *Paracorollospora*, by a p-distance of 1% from *Paracorollospora marina* and 3% from *Paracorollospora angusta* (Figure 2; Table 3; File S4). This new combination (*Paracorollospora luteola*) was created despite segregating by just 1% from *Paracorollospora* marina, which is an inconclusive value, only indicative of belonging to the same genus. The recommended evaluation also included the ITS region, which was not possible because only the strain *Sigmoidea marina* TUB557, without other published sequences, has an inconclusive short ITS sequence (381 nt) (Table 3; Table S1). However, a previous study involving the marine *Sigmoidea* species [61], found clear morphological differences in type material from both species, at that time designated as *Sigmoidea marina* and *Sigmoidea luteola*. This was taken into account in the present study, conducting the nomination of *Paracorollospora luteola* (Table 3). 

The 28S global assessment also confirmed the sectorial analyses it’s it’s ading it’s aITS and 28S regions and gave additional information, about the controversial placements of allegedly two morphs of the same organism: *C. intermedia* NBRC 104402 and *Varicosporina prolifera* NBRC 32120. *Corollospora intermedia* appears in a highly supported clade (PP = 1; BS > 90) with the three species of *Corollospora* recognized in the present study (p-distances of 2%/3%/5% from *C. quinqueseptata*/*C. maritima*/*C. mediterranea*); *Varicosporina prolifera* NBRC 32120 clusters with the two *Corollosporella* species (p-distance = 1–2%) in the *Corollosporella* clade, with a highly supported PP value (Table 3; File S4; Figure 2). While the placement of *Varicosporina prolifera* in the genus *Corollosporella* makes sense, phylogenetically joining morphologically related species (all have asexual morphs in *Varicosporina*), the placement of *C. intermedia* in the *Corollospora* clade is controversial (p-distance from the others ≥2%) (Table 3 and Table S2).

### 3.6. Additional Assessments

#### 3.6.1. Evaluations Based on the 18S Gene

The 18S gene assessment was limited to the members evaluated in the MSAs performed for the ITS and 28S regions, excluding *Tokurathelia colossa* NBRC 32103 (type strain) and *Honshuriella fusca* NBRC 32108. The 18S sequences from these isolates were excluded because, being from different species, they are inexplicably identical (Tables S1 and S2; Section 3.4.2).

The minimum value of p-distance (0%) was always found between sequences from the same species, but also between sequences of species from the same genus (*C. maritima* and *C. mediterranea*) or different genera (*Garethelia parvula* and *Shirahamella gracilis*; *Ajigaurospora pseudopulchella* and pNGenus D (the current *C. cinnamomea*); 1% and 2% were not discriminative considering that 2% was found within the same genus (between two out of the three *Corollospora* species) and 1% was frequently found in situations involving species from different genera (Table S4; not clearly evidenced in Figure 3); the maximum values were found between species of different genera (3%) and between the outgroup (*Cucullosporella mangrovei*) and the others (3–4%) (Figure 3; File S4). 

Considering that only values of p-distance ≥3% could unequivocally be attributed to sequences from species of different genera, the main finding was the impossibility of designating p-distance thresholds for species or genera segregation. However, it is worth noticing that 3% was the value found between the *Corollospora* species (including *C. maritima* NBRC 32117) and the single representative of the new genus *Corollosporopsis* (*Corollosporopsis* sp. 3, registered as *C. maritima* NBRC 32118). This result supports the previously reported segregation of the *C. maritima* complex into several species, these two species having been recognized as belonging to different genera.

Regarding the phylogenetic analysis based on the 18S MSA, highly and well-supported clades only cluster sequences from the same species or from the same genus, except for the following three genera: *Ajigaurospora* and pNGenus D cluster in a highly supported sub-clade (p-distance = 0%), which clusters with pNGenus D in a well-supported clade (p-distance = 1%). These results corroborate the already referred lower discrimination of the 18S gene for segregation at species and genus levels.

#### 3.6.2. Phylogenetic Analyses Based on a Concatenated MSA 

**Figure 4:**
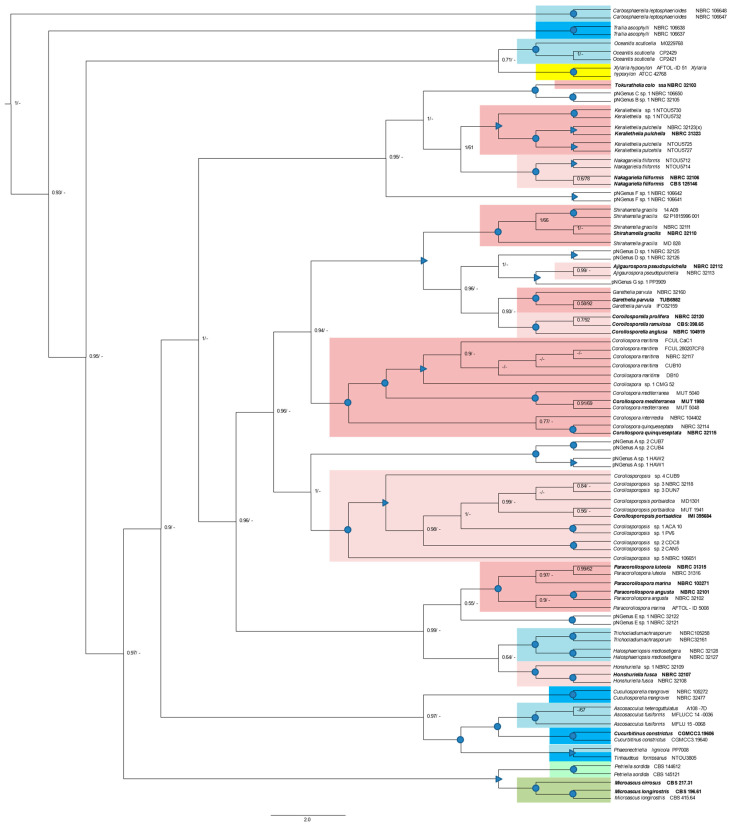
Phylogenetic tree based on 94 concatenated new and retrieved nuclear rDNA 28S/ITS/18S sequences representative of Microascales (Halosphaeriaceae and Microascaceae) and Xylariales taxa assembled in a 1682 positions MSA. Posterior probability (PP) values from the Bayesian analysis followed by bootstrap (BS) values from the maximum likelihood analysis are added to the right of the nodes (PP/BS). Circles signalize highly supported nodes (PP = 1; BS ≥ 90), and triangles signalize well-supported nodes (PP ≥ 0.95; BS ≥ 70); values of PP < 0.5 and Bs < 50% are signalized by “-”. All sequences are labeled with the strain name and accession number. Each generic clade is shaded: pink tones for *Corollospora* and the new genera, blue tones for the other Halosphaeriaceae genera, green tones for the Microascaceae genera outside the Halosphaeriaceae, and yellow for the representatives of the Xylariales (outside group). The putative new genera are in non-highlighted clades. Names in bold signalize type strains. Current species comprising the putative new genera: pNGenus A: *C. maritima*; pNGenus B: *C. colossa*; pNGenus C: *C. colossa*; pNGenus D: *C. cinnamomea*; pNGenus E: *C. lacera*; pNGenus F: *C. pseudopulchella* (NBRC 106641 + NBRC 106642); pNGenus G: C. *quinqueseptata* PP3909.

A phylogenetic tree (Figure 4) generated from Bayesian and ML analyses based on a concatenated ITS/28S/18S MSA, included 73 sequences from the former sectorial alignments and 21 new sequences: 14 representatives of other Halosphaeriaceae genera, five representatives of genera belonging to another family (Microascaceae) of the same order (Microascales), and two representatives of a genus (*Xylaria*) belonging to another order: Xylariales (File S3; Figure 4). The minimum number of positions in this alignment is 408 (just the 28S gene) and the maximum number is 1682 (the three regions).

The genus *Corollospora*, eight new genera, and three pNGenera, cluster in distinct and highly supported clades (PP = 1; BS > 90), while the genus *Ajigaurospora* clusters in a well-supported clade (PP ≥ 0.97; Bs ≥ 69). The genus *Tokuratelia*, only represented by one member (the type of strain) and with a single valid sequence (28S; see Section 3.4.2) also clusters in a well-supported clade with the representatives of two pNGenera (B and C) which, as also seen in the ITS and 28S gene trees (Figure 1 and Figure 2), cluster together in a highly supported sub-clade (Figure 4). This result was previously discussed, evidencing the need for future studies directed at what may be three new genera hidden in a complex genus (former genus *C. colossa*).

Some general cluster together in highly supported clades, but only for PP values: *Keraliethelia*, *Nakagariella*, *Tokuratelia*, and the pNewGenera B, C; *Corollosporopsis* and pNGenus A; *Ajigaurospora*, *Corollosporella, Garethelia*, *Shirahamella*, and pNGenus D; *Paracorollospora*, pNGenus E, and the two morphs of a species belonging to another Halosphaeriaceae genus: *Halosphaeriopsis* (Figure 4).

Each outgroup Halosphaeriaceae genus cluster in highly supported clades, and some of these also group together. *Ascosacculus* and *Cucurbitinus* cluster together and with *Phaeonectriella* and *Tinhaudeus* (highly supported PP and Bs values), and all these clusters with *Cucullosporella* (highly supported PP value). In an identical situation the two genera of Microascaceae: *Petriella* and *Microascus* cluster together in a well-supported clade (PP ≥ 0.95; BS ≥ 70) (Figure 4).

As just described, the phylogenetic relationships between several new genera are identical or even less supported than those found for well-recognized genera belonging to the Microascales. These results highly support the establishment of the new halosphaeriacean genera proposed in the present study.

Globally, these results agree with the single-gene phylogenies and confirm the good resolution of the ITS phylogeny alone. They also evidence three Halosphereaceae genera (*Carbosphaerella*, *Trailia*, and *Oceanitis*) segregating from the others in an identical or even more distant mode as *Xylaria*, which belongs to a different order (Xylarialles).

## 4. Taxonmy

### 4.1. Corollospora sensu Stricto

*Corollospora* Werderm. Notizbl. Bot Gart Berlin-Dahlem 248. 1922.

**Type species:** *Corollospora maritima*.

Werderm., Notizbl. Bot. Gart. Berlin-Dahlem 8 (73): 248. 1922.

Index Fungorum number: IF 270595.

**Synonym:** *Peritrichospora integra* Linder, Farlowia 1(3): 414. 1944.

Host-Substratum/Locality: on maritime wood/Germany.

List of Corollospora sensu stricto:

# *Corollospora martima* Werderm., Notizbl. Bot. Gart. Berlin-Dahlem 8: 248. 1922.

# *Corollospora mediterranea* A. Poli, E. Bovio, G. C. Varese & V. Prigione, Appl. Microbiol. 11 (12, no 5452): 18. 2021.

# *Corollospora quinqueseptata* Nakagiri, In Nakagiri & Tokura, Trans. Mycol. Soc. Japan 28: 430. 1988.

***Corollospora sensu lato*** **:**

*Corollospora armoricana* Kohlm. & Volkm.-Kohlm. Can. J. Bot. 67: 1281. 1989.

*Corollospora baravispora* Steinke & E. B.G. Jones, Fungal Diver. 35: 88. 2009.

*Corollospora besarispora* Sundari, in Sundari, Vikineswary Yussoff & Jones, Mycol. Res 100: 1259. 1996.

*Corollospora borealis* S. Tibell, Svensk Mykologisk Tidskrift 37: 47. 2016.

*Corollospora californica* Kohlm. & Volkm.-Kohlm., Bot. Mar. 40: 225. 1997.

## *Corollospora cinnamomea* Jorg. Koch, Nordic Jl. Bot. 6: 49. 1986.

*Corollospora indica* Prasannarai, Ananda & K. R. Sridhar, J. Environ. Biol. 21: 235. 2000.

# *Corollospora intermedia* I. Schmidt, Natur, Naturschutz Mecklenberg 7: 6. 1970.

## *Corollospora lacera* (Linder) Kohlm., Ber. dt. Bot. Ges 75: 126. 1962.

*Corollospora mesopotamica* Al- Saadoon, Marsch Bulletin 2: 135. 2006.

*Corollospora novofusca* Kohlm. & Volkm.-Kohlm., Bot. Mar. 34: 34. 1991.

#-molecular data (not from type material).

##-pNGenus (present study).

**Figure 5:**
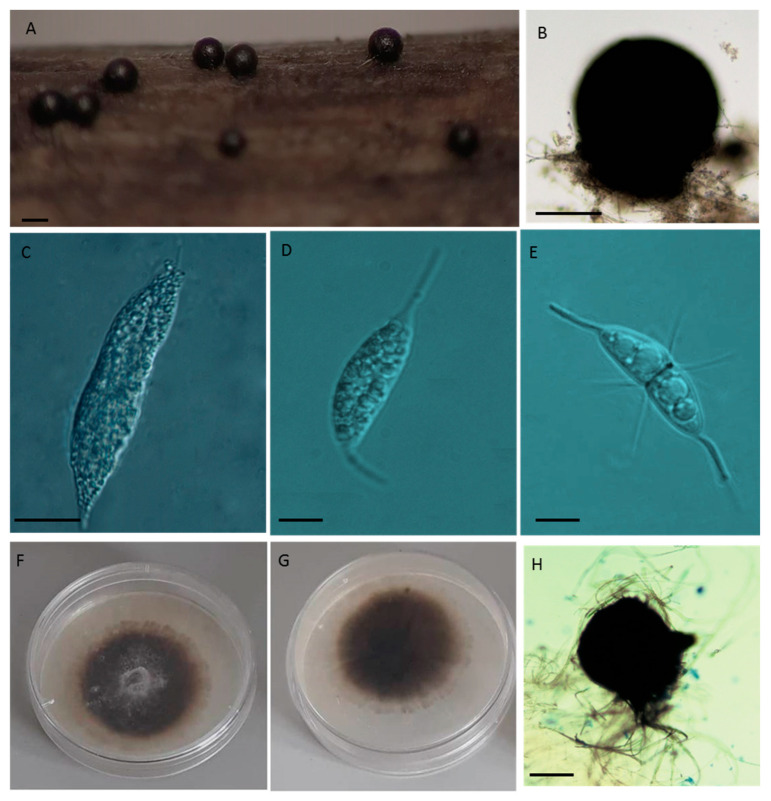
Morphological features of *Corollospora maritima*: (**A**) *Ascomata* on substrate; (**B**) *Ascoma;* (**C**) Ascus; (**D**) Immature ascospore; (**E**) Mature ascospore; (**F**,**G**) Colony on 50% seawater Corn Meal Agar, after 15 days of incubation: (**F**) Obverse, (**G**) Reverse; (**H**) *Ascoma* in 50% seawater Water Agar. Bars: (**A**) = 300 µm; (**B**) = 150 µm; (**C**)= 40 µm; (**D**,**E**)= 10 µm; (**H**) = 100 µm.

**Material examined: Portuguese isolates of *Corollospora maritima* (present study)** Figure 5.

Saprobic. **Sexual morph**: *Ascomata* 152–400 μm (276 ± 54.1 μm, n = 100), globose, carbonaceous, superficial, metallic black, solitary, with a short conic papilla. *Asci* 59–124 × 18−81 μm (93 ± 17.6 × 42.6 ± 11.2 μm, n = 75), 8-spored, fusiform to subclavate, unitunicate, early deliquescent. *Ascospores* 22–32.5 × 6–12.5 μm (27.5 ± 2.11 × 9.1 ± 1.06 μm, n = 600) hyaline, fusiform to sub-ellipsoidal, 1-septate constricted at the central septum. *Appendages:* primary polar single spines 8–19 × 1.25 µm (13.6 ± 2.3 × 1.6 ± 0.6 μm, n = 220); secondary equatorial 4–13.5 × 1.25 μm (9.3 ± 2.9 × 1.2 ± 0.01 μm, n = 110) around the central septum and apical 5–12.5 × 1.25 μm (8.5 ± 3.2 × 1.25 ± 0.01 μm, n = 110) (Figure 5; Table 1). **Asexual morph**: Undetermined.

**Note:** *Corollospora maritima* was phylogenetically based on 104 isolates (11 from the present study) (Table 1; Table S1). Phylogenetic analyses including pairwise distance assessments based on ITS and/or 28S sequences fall within the criteria set for the delineation of species and genera within the Halosphaeriaceae (Files S1–S4; Table 2 and Table 3). The closest relatives of *C. maritima* are the other *Corollospora* species, segregating by p-distances of 4–9%/2–4% in the ITS/28S regions (File S4; Figure 1 and Figure 2). The genus *Corollospora* was placed in a highly supported clade (A), segregating from the other genera by p-distances ≥14% in the ITS region or ≥5% in the 28S gene [Figure 1 and Figure 2 (clade A); File S4].

### 4.2. New Genera and New Combinations

***Ajigaurospora* E. Azevedo, P. Correia & M.F. Caeiro gen. nov.**

**MycoBank MB** **848509.**

**Etymology**: referring to the geographical origin of this species: Ajigaura [12].

Saprobic. **Sexual morph:** *Ascomata* solitary, superficial, globose to subglobose, ostiolate, papillate, black, carbonaceous. Pseudoparenchyma thin-walled of polygonal or rounded cells that fill the center of young ascocarps. Two layered *peridium:* outer layer formed by roundish cells; inner layer made by elongated cells. Paraphyses absent. *Asci* 8-spored, fusiform to ellipsoidal, unitunicate, early deliquescing. *Ascospores* fusiform, slender, hyaline, 7–11 septate, attenuate apices towards both ends, without polar spines; secondary appendages developed by the fragmentation of the exospore, peritrichous polar and equatorial around the central septum [12]. **Asexual morph**–Undetermined.

**Type species**: *Ajigaurospora pseudopulchella* (Nagakiri and Tokura) E. Azevedo, P. Correia & M.F. Caeiro.

**New combination:**

***Ajigaurospora pseudopulchella* (Nakagiri & Tokura) E. Azevedo, P. Correia & M.F. Caeiro comb. nov. MycoBank MB848986.**

**Basiomym:** *Corollospora pseudopulchella* Nakagiri & Tokura, Trans. Mycol. Soc. Japan 28: 418. 1987. 

**Distribution:** Japan [12], India [62]; South Africa [63]; Mexico [64]; Cuba [65]; Thailand [66]; Denmark [67].

**Holotype**: IFO 32112 (=TKB-C-1458 = AN-841 = NBRC 32112). 

**Notes:**

*Ajigaurospora pseudopulchella* is distinguished from *Corollospora* species by having ascomata with smooth surfaces and ascospores 65–97.5 × 8–12 µm, fusiform, 7–11 septate with attenuate apices (Table 3); [12].

Phylogenetically based on two strains of *Ajigaurospora pseudopulchella* including the type species NBRC 32112. Phylogenetic analyses including pairwise distance assessments based on ITS and/or 28S sequences fall within the criteria set for the delineation of genera within the Halosphaeriaceae (Files S3 and S4; Table 3). *Ajigaurospora* was placed in a distinct clade (G), and segregated by 15–16%/6% in the ITS/28S regions from *C. maritima*; the closest relatives are *Garethelia parvula* (p-distance = 8%) in the ITS region and the member of a putative new genus (pNGenus G) (p-distance = 2%) in the 28S gene [Figure 1 and Figure 2 (clade G); File S4].

 

***Corollosporella*** **E. Azevedo, P. Correia & M.F. Caeiro gen. nov.**

**MycoBank MB851370.**

**Etymology:** Morphological characters resembling *Corollospora.*

Saprobic. **Sexual morph**: *Ascomata* solitary or gregarious, superficial, ostiolate, papillate, black, coriaceous, or carbonaceous. The venter of immature ascomata is filled by thick-walled, hyaline roundish pseudoparenchymatous cells deliquescent, with pitted walls, deliquescent. *Peridium* with thick-walled cells, brown, composed of two layers: polygonal, roundish cells on the outer layer and flat cells on the inner layer. *Asci* 8-spored, ellipsoidal, unitunicate, early deliquescing. *Ascospores* fusiform to ellipsoidal, 1-septate, constricted at the central septum, hyaline. *Primary appendages* present at each end of the spore; secondary appendages are apical and equatorial (peritrichous, around the central septa), formed by fragmentation and peeling of the exospore [12,13,14] **Asexual morph**: *Conidiophores* simple or branched, multiseptate, hyaline. *Conidiogenous cells* proliferating, sympodial at the apex or monoblastic. *Conidia* septate, hyaline, branched, filamentous, which disarticulate into small segments; or conidia consisting in a system of axes; a main axis with two, rarely three side branches, each side branch arising from the previously developed branch [13,68].

**Type species**: *Corollosporella anglusa* (Abdel-Wahab & Nagah.) E. Azevedo, P. Correia & M.F. Caeiro comb. nov. 

**New combinations:**

1. ***Corollosporella anglusa* (Abdel-Wahab & Nagah.) E. Azevedo, P. Correia & M.F. Caeiro comb. nov. MycoBank MB851371.**

**Basionym:** *Corollospora anglusa* Abdel-Wahab & Nagah., Mycoscience 50(3): 149. 2009.

**Synonyms:** 

*Varicosporina anglusa* Abdel-Wahab & Nagah., Mycoscience 50(3): 150. 2009.

*Corollosporella anglusa* (Abdel Wahab & Nagah.) E. Azevedo, P. Correia & M.F. Caeiro, J. Fungi 9 (8, no. 841): 26 (2023), nom. inval., Art. 41.5 (Shenzhen).

**Distribution:** Egypt (Abdel-Wahab et al. 2009).

**Holotype**: IMI 395681, **Type strain**: MF 827 (=NBRC 104919).

2. ***Corollosporella ramulosa* (Meyers & Kohlm.) E. Azevedo, P. Correia & M.F. Caeiro comb. nov. MycoBank MB851372.**

**Basionym:** *Varicosporina ramulosa* Meyers & Kohlm., Can. J. Bot. 43: 916. 1965.

**Synonyms:** 

*Corollospora ramulosa* (Meyers & Kohlm.) E.B.G. Jones & Abdel-Wahab, in Réblová et al., IMA Fungus 7(1): 137. 2016.

*Corollosporella ramulosa* (Meyers & Kohlm.) E. Azevedo, P. Correia & M.F. Caeiro, J. Fungi 9 (8, no. 841): 26 (2023), nom. inval., Art. 35.1 (Shenzhen).

**Holotype**: F-521 B, **Type strain**: CBS 398.65.

**Notes:** *Corollosporella* gen. nov. is proposed to accommodate the species *Corollospora anglusa* and *C. ramulosa*. *Corollosporella anglusa* (type species) and *Corollosporella ramulosa* cluster together and apart from the type species of *Corollospora sensu stricto* (Figure 1 and Figure 2; Table S1). The morphological and molecular bases for the new combinations are presented in Table 3 and Table 4 and File S4.

*Corollosporella anglusa* differs from *Corollospora* species by having coriaceous smaller ascomata 60–110 µm, narrower 1-septate ascospores [18–30 × 3–4(−5) µm], with shorter polar spines (4–7 µm). The asexual morph *Variscosporina anglusa,* is characterized by the production of conidia formed of rectangularly branching filaments, 300 µm long, disarticulate to give bi-celled or rarely one-celled segments, 16–42 × 2–5 µm, hyaline, cylindrical, septate, constricted at the septa; and producing abundant chlamydospores in culture (Table 3); [13]. 

The morphological features described above, as well as the distinctive molecular data described below, supported the introduction of *Corollosporella* gen. nov. to accommodate the two species (Table 3 and Table 4). 

Phylogenetically based on type of species *Corollosporella anglusa* and four strains of *Corollosporella ramulosa*. Phylogenetic analyses including pairwise distance assessments based on ITS and/or 28S sequences fall within the criteria set for the delineation of genera within the Halosphaeriaceae (Files S3 and S4; Table 3). The two species of *Corollosporella* cluster in a highly supported clade (D) (only in the ITS region), and segregated by 16–17%/6–7% in the ITS/28S regions from *C. maritima;* the closest relatives are *Ajigaurospora pseudopulchella* (p-distance = 9% in the ITS region) and a putative new genus (pNGenus D) (p-distance = 2% in the 28S gene) [Figure 1 and Figure 2 (sub-clades D1, D2); File S4].

 

***Corollosporopsis*** **M.F. Caeiro, P. Correia & E. Azevedo gen. nov.**

**MycoBank MB851352.**

**Etymology**: Reference to the morphological similarity with *Corollospora.*

Saprobic. **Sexual morph:** *Ascomata* solitary, superficial, globose, ostiolate, papillate, black, carbonaceous. Pseudoparenchymatous thick-walled cells, polygonal, with pit connections in their walls, fill the centrum of the immature fruit body, deliquescing. *Asci* 8-spored, broadly fusoid, unitunicate, early deliquescing. *Ascospores* 1-septate, fusiform, hyaline or brown, smooth-walled, one-septate, constricted at the central septum. Primary appendages are single, terminal at each end of the spore, spine, or thorn-like; secondary appendages developed by the fragmentation and peeling of the exospore, equatorial double frill or ribbon-like, polar forming a tube or sheets (adapted from [13,15,18,25]). **Asexual morph:** Undetermined.

**Type species**: *Corollosporopsis portsaidica* (Abdel-Wahab & Nagah.) M.F. Caeiro, P. Correia & E. Azevedo comb. nov.

New combination:

***Corollosporopsis portsaidica* (Abdel-Wahab & Nagah.) M.F. Caeiro, P. Correia & E. Azevedo comb. nov. MycoBank MB851353.**

**Basionym**: *Corollospora portsaidica* Abdel-Wahab & Nagah., Mycoscience 50: 152. 2009.

**Synonym:** *Corollosporopsis portsaidica* (Abdel-Wahab & Nagah.) M.F. Caeiro, P. Correia & E. Azevedo, J. Fungi 9 (8, no. 841): 27 (2023), nom. inval., Art. 41.5 (Shenzhen).

**Holotype**: IMI 395684; **Type strain:** MF 832 (=NBRC 105265).

**Notes:**

*Corollosporopsis portsaidica* differs from *Corollospora* species by having black, smaller ascocarps with thin (4–6 μm) pericardial walls, composed of one layer of cells. Ascospores ranging from 27–2 × 8–10 µm (type species) to 19–25 × 11–16 µm (Saudi strain), one-septate, constricted at the central septum; the two cells are similar in size and shape or slightly different, hyaline when immature and becoming brown at maturity (Table 3); [13,15]. These distinctive morphological features and the molecular data presented below supported the transfer to *Corollosporopsis* gen. nov. (Table 2, Table 3 and Table 4).

**Distribution:** Egypt, Saudi Arabia.

**Other species:**

Moreover, group in *Corollosporopsis* 59 isolates currently named *Corollospora maritima*: the strains NBRC 106651, NBRC 32118, and 57 (5 + 52) isolates previously identified as lineages 4 and 5 [18,25]. Highly supported molecular data indicate that they may constitute five species within the genus *Corollosporopsis* (sp. 1 to sp. 5), but they could not be defined due to the absence of published specific morphological features and the inability to designate type strains (Table S1).

The complete data concerning the p-distances within the genus *Corollosporopsis* (60 strains, including but not limited to the lineages 4 and 5 [18,25] may be found in Table 2, Files S1 and S2, and in Section 3.4.1 (under de-designation of Group 2). The final molecular evaluations only included representative sequences from each species/putative species. 

Phylogenetically based on three strains of *Corollosporopsis portsaidica* including the type species *Corollosporopsis portsaidica* MF 832, and eight representatives of 57 isolates of the genus *Corollosporopsis.* Phylogenetic analyses including pairwise distance assessments based on ITS and/or 28S sequences fall within the criteria set for the delineation of genera within the Halosphaeriaceae (Files S3 and S4, Table 3). *Corollosporopsis* was placed in a well-supported clade (B), segregating from *C. maritima* by 16–18%/7–9% in the ITS/28S regions; closest relatives: members of a putative new genus (pNGenus A) only represented by ITS sequences (p-distance = 13–17%), and *Corollosporella anglusa* (p-distance = 5%) in the 28 S gene. The *Corollosporopsis* isolates (only three with 28S sequences), often split in the ITS phylogeny in highly or well-supported sub-clades, indicative of six species (intra-species p-distance = 1–2%) belonging to the same genus (inter-species p-distance = 3–11%) [Figure 1 and Figure 2 (sub-clades B1 to B6); File S4].

**In summary**, *Corollosporopsis* gen. nov. includes six species (listed below) but only *Corollosporopsis portsaidica* with morphological descriptions [13,15], was established as a new combination. Each species is genetically defined by low p-distance thresholds: 2% in the ITS region and 0% in the 28S gene.

List of *Corollosporopsis* species based on geographical distribution and SH access in the UNITE platform [40]:

*Corollosporopsis portsaidica* – strains MF 832, MD 1301, MUT 1941 [13,15]. Egypt, Saudi Arabia, Italy.

SH1287719.08FU (https://unite.ut.ee/bl_forw_sh.php?sh_name=SH1287719.08FU#fndtn-panel1, accessed on 20 December 2022) [69]. 

*Corollosporopsis* sp. 1—50 isolates from the lineage 5 [18,25]. Cuba, Equador, Japan, Mexico, Panama.

SH1287718.08FU (https://unite.ut.ee/bl_forw_sh.php?sh_name=SH1287719.08FU#fndtn-panel1 accessed on 20 December 2022) [69]. 

*Corollosporopsis* sp. 2—five (all) isolates from the lineage 4 [18,25]. Mexico.

SH1177361.08FU (https://unite.ut.ee/bl_forw_sh.php?sh_name=SH1287719.08FU#fndtn-panel1 accessed on 20 December 2022) [69]. 

*Corollosporopsis* sp. 3—strain NBRC 32118 and isolate DUN7 [18,25]. Japan. SH1287719.08FU (https://unite.ut.ee/bl_forw_sh.php?sh_name=SH1287719.08FU#fndtn-panel1 accessed on 20 December 2022) [69]. 

*Corollosporopsis* sp. 4—isolate CUB9 [18,25] (Cuba).

SH1287719.08FU (https://unite.ut.ee/bl_forw_sh.php?sh_name=SH1287719.08FU#fndtn-panel1 accessed on 20 December 2022) [69]. 

*Corollosporopsis* sp. 5—strain NBRC 106651 (Japan). Not evaluated in UNITE.

 

***Garethelia* E. Azevedo, P. Correia & M.F. Caeiro gen. nov.**

**Mycobank MB848507.**

**Etymology**: In honor of the marine mycologist E.B. Gareth Jones.

Saprobic. **Sexual morph:** Undetermined. **Asexual morph:** *Conidiophores* hyaline, pleurogenous in the mycelium, simple, usually 20 µm long, unbranched, or sparsely branched, smooth, and thin. *Conidiogenous cells* are holoblastic, terminal sympodial, or irregularly sympodial and denticulate at the apex, lacking cytoplasmatic content. *Conidia* abundant, solitary or in groups, septate, typically slightly constricted at the septa, thin-walled, hyaline, filiform curved, C or U shaped, developed from the blowout end of *conidiogenous cells* or hyphal branch.

**Type species**: *Garethelia parvula* (Zuccaro, J.I.Mitch. & Nakagiri) E. Azevedo, P. Correia & M.F. Caeiro comb. nov.

**New combination:**

***Garethelia parvula*** **(Zuccaro, J.I.Mitch. & Nakagiri) E. Azevedo, P. Correia & M.F. Caeiro comb. nov. MycoBank MB848508.**

**Basiomym:** *Corollospora parvula* (Zuccaro, J.I.Mitch. & Nakagiri) E.B.G. Jones, K.L. Pang & Abdel-Wahab.

**Synonym:** *Halosigmoidea parvula* Zuccaro, J.I. Mitch. & Nakagiri, Bot. Mar. 52: 355. 2009.

**Distribution**: Germany, Japan.

**Holotype**: TUB 6989, **Type strain**: CBS 116644. 

**Notes:**

*Garethelia parvula* produces conidia (32−)85–125(−140) × 3–5 µm (including ending without cytoplasmatic content), solitary or in groups, filiform, curved, C-U shape, 4–8(−10) septate, typically with five middle cells slightly constricted at the septa. Terminal cells of mature conidia are empty of cytoplasm; proximal cells are mostly obtuse, and the distal end is filiform (rarely both are obtuse or filiform) [17]. These conidial characters are distinct from other asexual morphs of *Corollospora*, supporting their transfer to *Garethelia* gen. nov.

Phylogenetically based on 11 strains including the type species *Garethelia parvula* CBS 116644. Phylogenetic analyses including pairwise distance assessments based on ITS and/or 28S sequences fall within the criteria set for the delineation of genera within the Halosphaeriaceae (Files S3 and S4; Table 3). *Garethelia* was placed in a highly supported clade (F), and segregated from *Corollospora maritima* by 14–16%/6% in the ITS/28S regions; the closest relative is *Ajigaurospora pseudopulchella* (p-distance of 8%/3% in the ITS/28S regions [Figure 1 and Figure 2 (clade F); File S4].

 

***Honshuriella*** **P. Correia, E. Azevedo & M.F. Caeiro gen. nov.**

**MycoBank MB848324.**

**Etymology:** Referring to the locality where the fungus was collected (Honshu).

Saprobic. **Sexual morph**: *Ascomata* superficial, solitary, globose to subglobose, papillate, large, carbonaceous, black. *Peridium* is wall-thick, comprising three layers: an outer layer of *texture angularis*, a middle layer with polygonal or roundish cells, and inner layer with flattened cells. *Pseudoparenchyma* with large, thin-walled cells filling the center of the immature fruit body, deliquescing. *Asci* 8-spored, fusiform or clavate, thin-walled, unitunicate, early deliquescing. *Ascospores* large, muriform, dark brown. Primary appendages are hyaline, torn-like at each end of the spore and formed by outgrowths of mesoporium and episporium; secondary appendages are formed by the fragmentation of exospore, peritrichous around the central septum and terminal, forming a sheet around the polar spine [12,14]. **Asexual morph**: Undetermined.

**Type species:** *Honshuriella fusca* (Nakagiri & Tokura) P. Correia, E. Azevedo & M.F. Caeiro comb. nov.

**New combination:**

***Honshuriella fusca* (Nakagiri & Tokura) P. Correia, E. Azevedo & M.F. Caeiro comb. nov. MycoBank MB848987.**

**Basionym:** *Corollospora fusca* Nakagiri & Tokura, Trans. Mycol. Soc. Japan 28: 424. 1987.

**Distribution:** Japan [12]; South Africa [64], Hawaii [70].

**Holotype:** TKB-F-5056; **Type strain**: NBRC 32107; **Culture**: TKB-C-1456 (=AN 724). 

**Notes:**

*Honshuriella* gen. nov. is proposed to accommodate *Corollospora fusca*, based on morphological and molecular data (Table 3 and Table 4). 

This species differs from other *Corollospora* species by having larger *ascomata* (264–440 µm in diameter) and a peridium wall formed by three layers (outer layer of *textura angularis*, middle layer with polygonal and roundish cells, and inner layer with flat cells), different from the two-layered peridium wall of *Corollospora*. Ascospores are large (63–220 × 20–28), fusiform, muriform, dark brown, and possess longitudinal striae of melanin in the mesosporial layer; characters not typical of the Halosphaeriaceae, and not included in the former generic description [12,71], and characters unique to the new genus *Honshuriella*.

Phylogenetically based on three strains including the type species *Honshuriella fusca* NBRC 32107. Phylogenetic analyses including pairwise distance assessments based on ITS and/or 28S sequences fall within the criteria set for the delineation of genera within the Halosphaeriaceae (Files S3 and S4, Table 3). *Honshuriella* was placed in a highly supported clade (K), and segregated from *C. maritima* by 22–24%/8–9% in the ITS/28S regions and from the closest species by 17–26%/6–9% in the ITS/28S regions. Considering the p-distances to *Honshuriella fusca* (7%/2% in the ITS/28S regions), the strain NBRC 32109 is proposed as *Honshuriella* sp. [Figure 2 and Figure 3 (sub-clades K1, K2); File S4].

 

***Keraliethelia* P. Correia, E. Azevedo & M.F. Caeiro gen. nov.**

**Mycobank MB848496.**

**Etymology**: In reference to the provenance of the type species (Kerala).

Saprobic. **Sexual morph:** *Ascomata* solitary or gregarious, superficial, or immersed, globose to subglobose with rough and tuberculate surface, ostiolate, papillate, carbonaceous, black. Two layered *peridium*: outer layer formed by roundish cells; inner layer made by elongated cells. *Pseudoparenchyma* thin walled constituted by polygonal or rounded cells that fill the center of young ascocarps. *Paraphyses* absent. *Asci* 8-spored, cylindrical to fusiform, short pedunculated, unitunicate, early deliquescing. *Ascospores* are fusiform, slightly curved, hyaline, usually 7-septate, constricted at the septa, with rounded apices, without primary appendages namely polar spines; secondary appendages developed by the fragmentation of the exospore, attached in a tuft to a conical papilla at the end, peritrichous around the central [16]. 

**Asexual morph:** *Conidiophores* cylindrical, hyaline, simple or branched, septate; conidia hyaline, light brown to brown, tetraradiate, several celled, slightly constricted at the septa, composed of one basal, bulbous, dark cell and with one or two crowns of radiating non-deciduous arms [16,50].

**Type species**: *Keraliethelia pulchella* (Kohlm., I. Schmidt & Nair) P. Correia, E. Azevedo & M.F. Caeiro comb. nov. 

**New combination:**

***Keraliethelia pulchella* (Kohlm., I. Schmidt and Nair) P. Correia, E. Azevedo & M.F. Caeiro comb. nov. MycoBank MB 848497.**

**Basionym:** *Corollospora pulchella* Kohlm., I. Schmidt et N.B. Nair, Ber. Dt. Bot. Ges. 80: 08. 1967, [MB#328947]. 

*Clavatospora bulbosa bulbosa* (Anastasiou) Nakagiri & Tubaki, Bot. Mar. 28 (11): 489. 1985.

**Synnonym:** *Clavariopsis bulbosa* Anastasiou 1962, Mycologia. 53(1): 11. 1962.

**Mode of life:** Saprobic on wood or on sand. 

**Distribution:** United States of America [16]; Australia [72]; India [73]; Baltic Sea [74,75]; Denmark [67]; Mexico [64]; Cuba [65]; Taiwan [66]; Thailand [76]; Portugal [31].

**Holotype:** Herb. J. K. No. 1975a (NY Barcode: 00966741); (CJ Anastasiou, USA 19). 

**Type strain**: *Clavatospora bulbosa* NBRC 31323 (=IFO 31323, =TKB-C-1246, =ATCC 14677, =CBS 179.62, =IMI 92610). 

**Notes:**

The transfer of *Corollospora pulchella* to *Keraliethelia* gen. nov, is based on morphological and molecular data from representatives of both morph types (Table S1, Table 2 and Table 3). 

The asexual morph of *Keraliethelia pulchella* differs from other asexual morphs of *Corollospora* by having conidia 20–70 × 4–6 µm, light brown, tetraradiate, slightly constricted at the septa, developed by the transformation of the swollen apex, formed of one basal bulbous, light brown, small cell (64–20 µm), with 14–2 crowns of radiating non-deciduous arms [16,50,61]. These morphological characters are distinct from the asexual states described for other *Corollospora* species. 

The sexual morph of *Keraliethelia pulchella* differs from other species of *Corollospora* in a number of morphological features: the tuberculate surface of the ascomata; ascospores 7-septate (rarely 9–13) with rounded apices, primary appendages (polar spines) absent*,* secondary polar appendages (about seven) attached in a tuft to the conical papilla and lateral appendages (about 15) placed around the central septa (Table 4); [16,50].

Phylogenetically based on 13 strains of *Keraliethelia* including the type species *Keraliethelia pulchella* (=*Clavatospora bulbosa*) NBRC 31323. Phylogenetic analyses including pairwise distance assessments based on ITS and 28S sequences (two and 13 sequences, respectively) fall within the criteria set for the delineation of genera within the Halosphaeriaceae (Files S3 and S4; Table 3). *Keraliethelia* was placed in a highly supported clade (L), and segregated from *C. maritima* by 24–26%/9–11% in the ITS/28S regions; the closest relatives are *Corollosporella anglusa* (p-distance = 20% in the ITS region) and *Corollospora maritima* in the 28S gene. Considering the p-distance (=4%) to the other members in the 28S gene, four species (without ITS sequences) were placed as *Keraliethelia* sp.; to notice the p-distances between the representatives of *Keraliethelia pulchella*: 3%/0–1% in the ITS/28S regions) [Figure 1 and Figure 2 (sub-clades L1, L2); File S4].

 

***Nakagariella*** **P. Correia, E. Azevedo & M.F. Caeiro gen. nov.**

**MycoBank MB848498.**

**Etymology:** In honor of the mycologist Akira Nakagiris, who described the species.

Saprobic. **Sexual morph:** *Ascomata* solitary, superficial, globose to sub-globose, black, carbonaceous, peridium with three layers, dark thick-walled cells: large, polygonal, paliform cells on the outside, flattened cells on the inside. *Pseudoparenchyma* with hyaline, thin-walled, polygonal or rounded cells filling the centrum of the ascomata; cell walls with pit-like thickenings connecting plasmatic strands of neighboring cells. *Paraphyses* absent. *Asci* eight-spored, early deliquescing. *Ascospores* are filiform, multi-septate, hyaline, without polar spines, secondary appendages equatorial, and polar formed by exospore fragmentation [12]. **Asexual morph**: Undetermined.

**Type species**: *Nakagariella filiformis* (Nakagari) P. Correia, E. Azevedo & M.F. Caeiro comb. nov.

**New combination:**

***Nakagariella filiformis* (Nakagari) P. Correia, E. Azevedo & M.F. Caeiro comb. nov. MycoBank MB848499.**

**Basiomyn**: *Corollospora filiformis* Nakagiri. Trans. Mycol. Soc. Japan 28: 418. 1987. 

**Distribution:** Japan [77], India [63,73], Thailand [66], Taiwan [76], South Africa [63], Sweden [78]. 

**Holotype**: TKB-F-5055. **Culture** derived from the type TKB-C-1455 (=AN-802) [12], with two representatives: CBS 125146 and NBRC 32106.

**Notes:**

*Nakagariella* gen. nov. is proposed to accommodate *Corollospora filiformis*, based on morphological and molecular data (Table 2, Table 3 and Table 4). *Nakagariella filiformis* is characterized by having large carbonaceous ascomata (243–418 µm), with a three-layered peridium with a distinctive paliform outermost layer of thick-walled large cells, heavily carbonaceous ascomata resistant to the sandy beach environment; ascospores are filiform, hyaline with 13(9–17) multi transverse septa, without polar spines. *Corollospora* species have smaller ascomata with a basic two-layered peridium, ascospores are fusiform or sub-ellipsoidal, presenting primary appendages (polar spines) and secondary appendages (polar and equatorial). These distinguishable morphological characters support the transfer to *Nakagariella* (Table 4); [12,79].

Phylogenetically based on five strains including two recognized as type species *Nakagariella filiformis* CBS 125146 and *Nakagariella filiformis* NBRC 32106. Phylogenetic analyses including pairwise distance assessments based on 28S and ITS sequences (restricted to *Nakagariella filiformis* CBS 125146), fall within the criteria set for the delineation of genera within the Halosphaeriaceae (Files S3 and S4; Table 3). *Nakagariella* was placed in a highly supported clade (M), and segregated from *C. maritima* by 19–20%/9–11% in the ITS/28S regions; the closest relatives are *Shirahamella gracilis* (p-distance = 16% in the ITS region) and members of a putative new genus (pNGenus D) (p-distance = 9% in the 28S gene) (File S4). The relationships just indicated are not evidenced in the phylogenetic trees, where *Nakagariella* (sub-clade M) and *Keraliethelia* (sub-clade L) cluster in a well-supported main clade, regardless the high p-distances between these two genera: 19–20%/8–11% in the ITS/28S regions) [Figure 1 and Figure 2; File S4].

 

***Paracorollospora*** **E. Azevedo, P. Correia & M.F. Caeiro gen. nov.**

**MycoBank MB848517**

**Etymology: Similarity to *Corollospora***

Saprobic. **Sexual morph:** *Ascomata* superficial, solitary, or gregarious, globose or sub-globose, carbonaceous, black, papillated. *Peridium* comprises two cell layers: the outer layer with dark, polygonal, or roundish cells, and the inner layer with flattened cells. *Paraphyses* absent. *Asci* eight-spored, fusiform or subcylindrical, unitunicate, early deliquescing. *Ascospores* fusiform, septate, hyaline. Primary appendages are short, spiniform at each apex, secondary appendages polar long, and equatorial around the central septa, peritrichous (Nakagiri and Tokura, 1987). **Asexual morph**: *Conidiophores* hyaline, pleurogenous on the mycelium, primarily short and simple becoming long and septate, thin and smooth-walled. *Conidiogenous cells* are holoblastic, terminal, sympodial or irregularly sympodial, and denticulate. *Conidia* solitary, C-shaped to slightly sigmoid, multiseptated, strongly constricted at septa, terminal and basal cells of mature conidia empty of cytoplasmatic content [62]. 

**Type species:** *Paracorollospora angusta* (Nagakari & Tokura) E. Azevedo, P. Correia & M.F. Caeiro comb. nov.

**New combinations:**

1. ***Paracorollospora angusta* (Nagakari & Tokura) E. Azevedo, P. Correia & M.F. Caeiro comb. nov. MycoBank MB848518.**

**Basiomyn:** *Corollospora angusta* Nakagiri and Tokura. Trans. Mycol. Soc. Japan 28: 418.1987.

**Distribution**: Sweden [79]; Denmark [67]; Portugal [31].

Holotype: TBB-F-5053; Type strain: NBRC 32101 (=TKB-C-1453 = AN-421) Ex-holotype.

2. ***Paracorollospora marina* (Haythorn & E.B.G. Jones) E. Azevedo, P. Correia & M.F. Caeiro comb. nov. MycoBank MB848519.**

**Basionym:** *Sigmoidea marina* Haythorn & E.B.G. Jones. Trans. Mycol. Soc. Japan 74: 620. 1980.

**Synonymy:** *Corollospora marina* (Haythorn & E.B.G. Jones) E.B.G. Jones, K.L. Pang and Abdel-Wahab. IMA 7: 131–153. 2016. 

*Halosigmoidea marina* (Haythorn et E.B.G. Jones) Nakagiri, K.L. Pang and E.B.G. Jones. Botanica Marina 52: 349–359. 2009. 

**Distribution**: Great Britain (Haythron et al. 1980).

Type strain: NBRC 103271 (Ex-type of *Sigmoidea marina*) (=AN-690), Portsmouth Polytechnic (EBG Jones, PP0423)

3. *Paracorollospora luteola* (Nakagiri & Tubaki) E. Azevedo, P. Correia & M.F. Caeiro comb. nov. Mycobank MB848520.

Basiomyn: *Corollospora luteola* Nakagiri & Tubaki. Trans Mycol. Soc. Japan 23: 102. 1982.

**Synonymy:** *Halosigmoidea luteola* (Nakagiri & Tubaki.). Nakagiri, K.L. Pang & E.B. Gareth Jones; Bot. Mar. 52: 355. 2009; *Sigmoidea luteola* (Nakagiri & Tubaki.) Trans. Mycol. Soc. Japan 23: 102. 1982.

**Distribution**: Japan [12]; India [62]; Sweden [78]. 

**Notes:**

*Paracorollospora* gen. nov. is proposed to accommodate *Corollospora angusta*, *Corollospora marina*, and *Corollospora luteola*, based on morphological and molecular data. 

*Paracorollospora angusta* differs from *Corollospora* species by having narrower ascospores (3–8 µm), 3(−5) septate with short polar spines (Table 4); [12,50]. *Paracorollospora luteola* also has narrower ascospores (5–8 µm), 5-septate [50]. The conidia of *Paracorollospora marina* are (80)110–180(−231) long, 4–7 μm wide near the middle, curved, C-shaped to slightly sigmoid, 7–11 septate, different from those produced by other two asexual states (*Paracorollospora luteola* and *Garethelia parvula* in the present study), formerly placed together in *Halosigmoidea* [61] and later transferred to *Corollospora* [6].

These morphological characters and molecular data support the transfer of these species to *Paracorollospora* gen. nov. (Table S1, Table 2 and Table 3).

Phylogenetically based on the type strains *Paracorollospora angusta* NBRC 32101 and *Paracorollospora marina* NBRC 103271, *Paracorollospora luteola* TKBC-1250 (=NBRC 32115) and other 20 strains from the three species. Phylogenetic analyses including pairwise distance assessments based on ITS and/or 28S sequences (*Paracorollospora luteola* only represented by 28S sequences) fall within the criteria set for the delineation of genera within the Halosphaeriaceae (Files S3 and S4; Table 3). *Paracorollospora* species group in a highly supported clade (E), and segregated from *C. maritima* by p-distances of 18–19%/7–8% in the ITS/28S regions; the closest species are *Garethelia parvula* (p-distance = 16–17% in the ITS region) and members of a putative new genus (pNGenus D) (p-distance = 4–5% in the 28S gene). As expected for species from the same genus, *Paracorollospora angusta* and *Paracorollospora marina* cluster apart from each other, and are segregated by 11% in the ITS region, The three species (including *Paracorollospora luteola*) are segregated by 1–3% in the 28S gene (1% between *Paracorollospora luteola* and *Paracorollospora marina*) [Figure 1 and Figure 2 (sub-clades E1, E2, E3); File S4].

 

***Shirahamella*** **E. Azevedo, P. Correia & M.F. Caeiro gen. nov.**

**MycoBank MB848514**

**Etymology**: referring to the geographical location of collection (Shirahama).

*Saprobic.* **Sexual morph:** *Ascomata* solitary or gregarious, superficial, globose to subglobose, ostiolate, papillate, black, carbonaceous. *Peridium* is thick, two-layered: the outer layer is composed of polygonal or roundish cells; the inner layer is composed of flat cells. *Pseudoparenchymatous* thick-walled cells, polygonal, with pit connections in their walls, fill the centrum of the immature fruit body, deliquescing. *Asci* 8-spored, fusiform to ellipsoidal, unitunicate, early deliquescing. *Ascospores* fusiform to hyaline, 1-septate. Primary appendages are single, terminal at each end of the spore, spine, or thorn-like; secondary appendages are fibrous or peritrichous, polar, and equatorial, developed by the fragmentation and peeling of the exospore. **Asexual morph**: Undetermined.

Type species-*Shirahamella gracilis* (Nakagiri & Tokura) E. Azevedo, P. Correia & M.F. Caeiro comb. nov.

**New combination:**

***Shirahamella gracilis* (Nakagiri & Tokura) E. Azevedo, P. Correia & M.F. Caeiro comb. nov. MycoBank MB848516.**

**Basiomym:** *Corollospora gracilis* Nakagiri & Tokura, Trans. Mycol. Soc. Jap. 28: 418. 1987.

Mode of life: saprobic

**Distribution:** Japan [12], Australia [72]; India [62]; South Africa [63]; Mexico [64]; Cuba [65]; Thailand [66].

**Holotype**: TKB-F-5057, **Type strain**: TKB-C-1457 (=NBRC 32110)

**Notes:**

*Shirahamella gracilis* differs from *Corollospora* species by having narrower one-septate ascospores [26–45 × 3–5.5(−7) µm]. This species produces ascomata readily and abundantly in single spore cultures under laboratory conditions (Table 4); [12].

Phylogenetically based on 18 strains including the type species *Shirahamella gracilis* TKB-C-1457 (=NBRC 32110). Phylogenetic analyses including pairwise distance assessments based on ITS and/or 28S sequences fall within the criteria set for the delineation of genera within the Halosphaeriaceae (Files S3 and S4; Table 3). *Shirahamella* was placed in a distinct and highly supported clade (H), and segregated from *C. maritima* by 14–16%/6% in the ITS/28S regions; the closest species is *Ajigaurospora pseudopulchella* (p-distances of 9%/2% in the ITS/28S regions); due to a p-distance of 4–5% (ITS region) to the other members, the strain MD 828 is proposed as *Shirahamella* sp. [Figure 1 and Figure 2 (sub-clades H1, H2); File S4].

 

***Tokurathelia*** **M.F. Caeiro, E. Azevedo & P. Correia gen. nov.**

**MycoBank MB848512.**

Etymology: In honor of the mycologist Tokura, who described the species.

Saprobic. **Sexual morph:** *Ascomata* superficial, solitary, or gregarious, settled with a subiculum on sand grains, globose to subglobose, black, carbonaceous. *Peridium* three-layered: an outer layer of hyaline, thick-walled, roundish large cells; a medium layer of polygonal or roundish cells; and an inner layer with flattened cells. The center of immature ascomata consists of polygonal or roundish, thin-walled, hyaline, pseudoparenchymatous cells. *Paraphyses* absent. *Asci* 8-spored, fusiform, thin-walled, unitunicate, early deliquescing. *Ascospores* fusiform or ellipsoidal, hyaline, multi-septate. Without polar spines, secondary appendages are formed by the fragmentation of exospore, long, fibrous, and peritrichous at each end of the spore and around the central septum [12]. **Asexual morph**: Undetermined.

**Type species: *Tokurathelia colossa*** (Nakagiri and Tokura) M.F. Caeiro, E. Azevedo & P. Correia comb. nov.

**New combination:**

***Tokurathelia colossa* (Nakagiri and Tokura) M.F. Caeiro, E. Azevedo & P. Correia** comb. nov. **MycoBank MB848513.**

**Basiomyn:** *Corollospora colossa* Nakagiri and Tokura, Trans. Mycol. Soc. Jap. 28: 418. 1987. 

**Distribution:** Japan [12], South Africa [63], Thailand [66], Malasia [80]. Taiwan [76], India [62]. 

**Holotype: TKB-F-5054 type strain TKB-C-1454 (=NBRC 32103)**

**Notes:**

*Corollospora colossa* is removed from *Corollospora* and placed in *Tokurathelia* gen. nov. based on morphological and molecular data (Table 2, Table 3 and Table 4). 

*Tokurathelia colossa* presents large ascomata (318–515 µm), with long papilla basal in position. The peridium differs from the basic two-layered peridium of *Corollospora*, and consists of three layers: the outer layer is composed of roundish, large paliform cells (absent in *Corollospora*), the middle layer is composed of polygonal or roundish cells, and the inner layer with flattened cells. Ascospores are characterized by large dimensions, 60–108 × 13–26 µm, 7-septate, without polar spines; secondary appendages long, fibrous, thick, peritrichous, equatorial, and terminal at each end of the spore, developed by fragmentation and peeling of the exospore. These morphological characters in *Tokurathelia colossa* distinguish it from *Corollospora* species (Table 4); [7,12,50].

Phylogenetically based on the type species *Tokurathelia colossa* NBRC 32103. Phylogenetic analyses including pairwise distance assessments based on 28S sequences fall within the criteria set for the delineation of genera within the Halosphaeriaceae (Files S3 and S4; Table 3). *Tokurathelia* was placed in a highly supported clade (N), and segregated from *C. maritima* by a p-distance of 12% (28S gene). The closest species (p-distance = 8–9%), grouping in a highly supported sub-clade (I/J), are two putative new species (strains NBRC 32105 and NBRC 106650, currently identified as *C. colossa*) [Figure 2 (clade N, sub-clade I/J); File S4].

The two pNSpecies referred to above appear to belong to another genus, or to other two genera (pNew genera B, C) considering that they segregate by 15%/3% in the ITS/28S regions [Figure 1 and Figure 2 (sub-clade I/J); File S4].

More morphological studies (involving new collections and type strains to be designated) and additional molecular evaluations (targeting the ITS region and genes codifying protein) are required to confirm the current molecular data and provide the eventual reassessment of these taxa.

### 4.3. Summary of Taxonomic Placements

**Figure 6:**
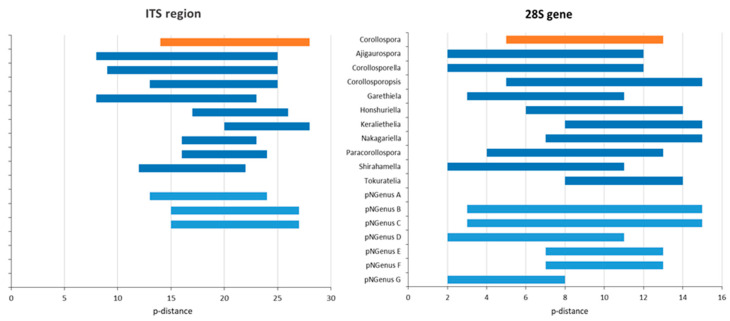
Range of p-distances evidenced between species of the genera defined in the present study.

To clarify the generic placements recognized in the present study, we provide the key to the new halosphaeriacean genera created from the former genus *Corollospora*. The p-distance ranges evidenced from the revised genus *Corollospora* and between them are summarized in Figure 6.

**Dichotomous Key to *Corollospora* and new genera within the Halosphaeriaceae**
**1.** Asexual morph with conidia………………………………………………………….…………………….………….……...…..…….……...…...**2****1.** Sexual morph with ellipsoidal to fusiform ascospores ……………………………………………..…….........……...…..……...…...…….......**5**
**2.** Conidia unbranched, sparsely branched or tetraradiate …........................................................…..................................................................**3****2.** Conidia rectangularly branched filamentous, which disarticulate into small segments or into a system of axes, *Variscosporina* asexual morph…………………………………..……………………………...……..………………………………….………………………***Corollosporella***
3. Conidia hyaline ………………………………………………...………………………………………..………….……………………..…………**4****3.** Conidia light brown, with bulbous dark cells and with one or two crowns of radiating non deciduous arms......................................................................................……..........................................................................................................***Keraliethelia***
**4.** Conidia filiform curved, C or U shaped………………………………….…….…………………...……………………..…...………***Garethelia*****4.** Conidia C-shaped to slightly sigmoid, multi-septate; terminal and basal cells of mature conidia empty of cytoplasmatic content…… ……………………………...……………………………………………….…………………………………………………………***Paracorollospora***
**5.** Ascospores mostly with polar spines ……………..………………………………….………………………..…....…………..….…………....**6**
**5.** Ascospores without polar spines ………………………………………………………………….……….…….……….…….……….…..........**13**

**6.** Ascospores brown, muriform, 63−220 µm in length ……………………………………………...…………………………....…***Honshuriella***6. Ascospores hyaline to brown, predominantly with transverse septa …………………….………..........................................................……**7**
**7.** Polar spines short (<8 µm) ………………………………………………………………………….……..........………….…………....................**8****7.** Polar spines > 8 µm ………………………………...............................................................................................................................................**10**

8. Ascospores 1-septate ……………………………………..………………...………………………………………………………………………...**9****8.** Ascospores 3–5 septate………..………………………………….……………………….………………….………..……….…***Paracorollospora***
**9.** Ascospores diameter mainly less than 8 µm, lacking asexual morph, abundant production of ascomata in culture …………………………………..............................................................................................................................................................***Shirahamella*****9.** Ascospore diameter 3−4(−5) µm with *Varicosporina* anamorph………………………..…………………….…………………***Corollosporella***
**10.** Ascospores 1-septate ……………………………………………………………………………….……………......……..……***Corollosporopsis*****10.** Ascospores 1-septate or more, with conidial chlamydospores…………………...………...……………………...……………***Corollospora***
**13.** Ascospores diameter wider than 12 µm ………………………………..………….…………………..…………………....…………………..**14****13.** Ascospores diameter narrower than 12 µm …………………………… ……………………………….………………………...…………....**15**
**14.** Ascospores mostly 7-septate, 52–112 µm with rounded apices, ascoma surface tuberculate............................................…***Keraliathelia*****14.** Ascospores 7–11 septate, 65–98 µm with attenuate apices; ascoma surface smooth.………...........................................…..***Ajigaurospora***
**15.** Ascospores fusiform or ellipsoidal, usually 7 septate ………………..………………....…………….........................................***Tokurathelia*****15.** Ascospores filiform, with 13(9−17) septa …………………………………………………...……...................................................***Nakagariela***

The present study evidenced *Corollospora* as a genus with only three species with morphological and/or molecular data: *C. mediterranea* morphologically characterized by chlamydospores in culture, *C. quinqueseptata* without published ITS sequences, and *C. maritima* with many isolates morphologically indistinct from species of other genera, which made it the former subject of the present study. Still to add *C. intermedia*/*Varicosporina prolifera* with a controversial placement, and two species not represented by type strains (*C. cinnamomea*, *C. lacera*) suggested as pNGenera (E and F).

The former genus *Corollospora*, in addition to the three species referred to above, also included the 13 species recognized as new combinations belonging to the 10 new genera established in the present study (Table 3 and Table 4; Section 4.2), and eight species without molecular data, which could not be subjected to the present evaluation. These species (listed in Section 4.1) remain in the genus *Corollospora* until molecular data may help to clarify the taxonomy.

From the phylogenetic and morphological data that distinguished different species within the genus *Corollospora*, this study established the 10 new genera defined in the present Dichotomous key, and seven putative new genera (pNGenus A to pNGenus G) (Figure 6). The pNGenera comprise species phylogenetically distinct either from the genus *Corollospora* or from the new genera to which they are morphologically related (Table 3).

Although several new genera (and pNGenera) segregate from each other by the lowest p-distances accepted in the present study (8%/2%, in the ITS/28S regions), the revised genus *Corollospora* segregated from the others by considerably higher p-distances: 14%/5%, in the ITS/28S regions (File S4, Table 3, Figure 6). These results are in accordance with the present taxonomic key, at the same time highlighting the need for molecular data for the resolution of morphological uncertainties.

## 5. Conclusions

This study proposes a set of phylogenetic and morphological criteria for the delineation of genera and species within the former genus *Corollospora*. The starting point was the disentangling of the *Corollospora maritima* complex.

Taking the reassessment of the *Corollospora* species as a model, a new phylogenetic approach was tested, starting with p-distance assessments to define appropriate thresholds for delineation of new taxa i.e., in accordance with currently accepted values [40,41,42]. The phylogenetic studies targeted separately each region of the nuclear rRNA cistron, and firstly each taxon was distinctly evaluated to select representative sequences (at a maximum p-distance and including type strains, whenever possible) to be subjected to final global assessments based on the selected sequences.

Morphological characterization of the species analyzed was based on the original descriptions, mainly focused on ascomatal wall structure, ascospore septation, and presence/absence of polar spines [7,12,19]. Based on these parameters, 10 new genera are introduced and seven putative new genera (most may belong to current cryptic species), were left for further evaluation.

Concerning the molecular data, to avoid the proposal of an inflated number of taxa, we based the segregation of species and genera in the ITS/28S regions on 3%/1% (highest p-distance values) and 12%/5% (lowest p-distance values), for species and generic segregation, respectively.

However, to keep apart morphologically distinct taxa, lower values were accepted for generic segregation (8%/2% in the ITS/28S regions), as for the species in a set of four new genera (*Corollosporella*, *Garethelia*, *Ajigaurospora*, *Shirahamella*). These values are still acceptable taking into account threshold values from large-scale evaluations [40].

With respect to segregation at the species level, 3% in the ITS region is the maximum p-distance accepted in UNITE for SH [40,41]), and is intended to prevent unnecessary new species. This value, and 1% for the 28S gene, were then accepted, except when morphological differences suggested lower values. This was found within the new genus *Corollosporopsis*, where 3% in the ITS region would gather morphologically distinct taxa (in ascospore color); a p-distance of 2% (the highest between two strains of *Corollosporopsis portsaidica*) was then established to segregate species of this genus.

Although the situations just referred to derive from effective morphological discriminations, the purpose and results of this study globally agree with the statement that fungi have limited morphological characters to differentiate them. Assuming large numbers of cryptic species in most genera, extensive molecular studies of fungal species have been considered necessary for the estimation of fungal diversity [53].

The *C. maritima* complex exemplifies the above considerations. Indeed, this study, initially targeting the disentangling of the cryptic species *Corollospora maritima,* ended up revealing a complex of three genera. Obviously, one of these is the genus *Corollopora*, encompassing the type species *Corollospora maritima* with more than 100 isolates assessed in the present study. Another is the new genus *Corollosporopsis* with a type species formerly established as *Corollospora portsaidica* [13] and putative new species, most genetically very close to the type species. Furthermore, a putative new genus (pNGenus A) was recognized, encompassing two species associated with two different geographical locations (Cuba and Hawaii) [18,25]; only one of these regions was also the collection site of another isolate of the former *C. maritima* complex: *Corollosporopsis* sp. 4, from Cuba.

From the phylogenetic results, in accordance with morphological data that distinguished different species within the former genus *Corollospora* (Section 4.3), 10 new genera with the following 13 new combinations of species, were established: *Ajigaurospora pseudopulchella*, *Corollosporella anglusa*, *Corollosporella ramulosa*, *Corollosporopsis portsaidica*, *Garethelia parvula*, *Honshuriella fusca*, *Keraliethelia pulchella*, *Nakagariella filiformis*, *Paracorollospora angusta*, *Paracorollospora luteola*, *Paracorollospora marina*, *Shirahamella gracilis*, and *Tokurathelia colossa*. 

Other members recognized as unidentified species were attributed to seven putative new genera (like the pNGenus A referred to above). So, new taxa are expected, although needing morphological characterization of type material and additional molecular data, to be assigned. Further studies on these species, the inclusion of members currently recognized as *Corollospora* spp., and new samplings, will aid other marine mycologists and provide a more comprehensive and accurate delineation of the revised genus *Corollospora* and support for the creation of new taxa.

To stress that these results were confirmed (Figure 4), with identical resolution, in more comprehensive phylogenies based on a concatenated MSA of the three regions of the nuclear rRNA cistron, involving the strains from the previous analyses and additional representatives from the same family (Halosphaeriaceae), other families from the same order (Microascales) and from a different order (Xylariales). 

The goals directed to the former genus *Corollospora* were achieved, validating the adopted phylogenetic methodology. We propose that this methodology be followed in assessments focused on identifications and/or establishment of new species and genera. It might also be very useful as a preliminary approach in studies needing more inclusive methodologies, as are the cases involving higher taxonomic ranges and/or diversity, and recognized problematic taxa.

## Figures and Tables

**Table 1:** Global data from the *Corollospora maritima* isolates identified in the present study.

Isolate	Source (Location)	Morphological Characters						Sequence ID	
		Ascocarp Diameter (µm)	Asci Dimensions * (µm)	Ascospores Dimensions * (µm)	Appendages Dimensions * (µm)			(GenBank)	
					Primary	Secondary		ITS Region	28S Gene
					Polar Spines	Equatorial	Polar		
FCUL CaC1	drift culm (a)	163	n.d.	22–32 × 6–10	8–14	3–6	3–6	OQ679052	OQ677997
FCUL CaC2	Sand (a)	152	n.d.	22–32 × 6–10	8–14	3–6	3–6	OQ679053	OQ677998
FCUL280207CF8	*Fagus sylvatica* bait (b)	212–307	59–113 × 25–43	25–32.50 × 7.50–10	10–15 × 1.25–2.50	7.5–10 × 1.25	5–7.5 × 1.25	OQ679054	OQ677999
FCUL280207CF9	*Fagus sylvatica* bait (b)	200–240	75–103 × 30–78	25–31.25 × 7.50–10	10–17.50 × 1.25	8.75–10 × 1.25	6.25–7.50 × 1.25	OQ679055	n.d.
FCUL280207CP10	*Pinus pinaster* bait (b)	180–283	96–124 × 30–40	23.75–30 × 7.50–11.25	12.50–17.50 × 1.25–2.5	8.75–10 × 1.25	6.25–8.75 × 1.25	OQ679056	n.d.
FCUL090707CP6	*Pinus pinaster* bait (b)	270–307	96–124 × 35–40	25–28.75 × 8.75–11.25	12.5–17.5 × 1.25	8.75–10 × 1.25	6.25 –7.50 × 1.25	OQ679057	n.d.
FCUL170907CP11	*Pinus pinaster* bait (b)	248.–400	59–124 × 25–59	20–30 × 7.5–10	7.50–15.00 × 1.25	7.5–12.50 × 1.25	5.0–7.50 × 1.25	OQ679058	OQ678000
FCUL010407SP8	*Pinus pinaster* bait (c)	249–378	70–85 × 19–35	25–32 × 7.50–10	8.75–18.75 × 1.25	8.75–13.75 × 1.25	8.75–10 × 1.25	OQ679059	n.d.
FCUL010407SP10	*Pinus pinaster* bait (c)	212–330	71–81 × 18–35	25–31.25 × 7.50–12.50	8.75–18.75 × 1.25	7.5–12.50 × 1.25	7.50–8.75 × 1.25	OQ679060	n.d.
FCUL130607SP8	*Pinus pinaster* bait (c)	212–340	70–124 × 30–78	26–31.25 × 7.50–10	8.75–18.75 × 1.25	8.75–13.75 × 1.25	7.50–10 × 1.25	n.d.	OQ678001
FCUL130607SP10	*Pinus pinaster* bait (c)	200–330	65–113 × 25–43	25–31.25 × 7.50–10	8.75–18.75 × 1.25	8.75–13.75 × 1.25	6.25–8.75 × 1.25	n.d.	OQ678002

(a): Cascais beach; (b): Cascais marina; (c): Sesimbra marina; *: lenght or lenght × width; n.d.: not determined.

**Table 2:** Summary of the data retrieved from the sectorial assessments directed to the current species of the genus *Corollospora*.

	Number of Members with Molecular Data		Number of Sequences		Summary of the Sectorial MSAs (File S1)			
TAXON Current Designation			A―Used		Number of Positions		p-Distance to *C. maritima sensu stricto*	
			B―Total (Retrieved)		p-Distance Values (File S2) (Notes in the Table S2)			
			ITS Region	28S Gene	ITS Region	28S Gene	ITS Region	28S
*Corollospora maritima* (includes the lineage 1 [18,25])	104 + 1● + 1 excluded	A	104 + 1(●)	21 + 1(●)	451 */436	524	n.a.	n.a.
					See notes and Section 3.5.1	See notes and Section 3.5.1		
		B	104 + 1(●)	21 + 1(●)				
*Corollospora portsaidica* + *Corollospora* *maritima* (strain NBRC 32118 + lineages 4 and 5 [18,25]) + strain NBRC 106651 (●)	60 (3 + 57) + 1●	A	2 + 58 + 1(●)	2 + 1 + 1(●)	451 */474	536	15–18%	7%
					See notes and Section 3.5.1	See notes and Section 3.5.1		
		B	2 + 58 + 1(●)	2 + 1 + 1(●)				
*Corollospora maritima* (lineages 2 + 3 [18,25])	11 + 1●	A	11 + 1(●)	0	451 */489	na	16–17%	―
					See notes and Section 3.5.1	―		
		B	11 + 1(●)	0				
*Corollospora* *anglusa*	1	A	1	1	601	535	16–18%	6%
					See notes	―		
		B	1	1				
*Corollospora* *angusta*	18	A	17	18	552	406/526	19–20%	6–7%
					0–1%; See notes	0–1%; See notes		
		B	17	18				
*Corollospora cinnamomea*	4	A	1	4	414	798	14%	5%
					See notes	0%		
		B	1	4				
*Corollospora colossa*	3 + 1 excluded	A	2	3	455	538	23–24%	11%
					Two strains: 12%	Three strains: 3% between two strains; ≥7% to the other strain. See notes		
		B	2	3				
*Corollospora filiformis*	5	A	1	5	613	507	18–19%	8–10%
					See notes	One group (4 strains) + one strain: 0–1% (within the group); 2% to the other strain; See notes		
		B	1	5				
*Corollospora fusca*	4	A	3	3	534	638	19–20%	7–8%
					One group (two strains) + one strain: 0% (within the group); 5% to the other strain; See notes	One group (two strains) + one strain: 0% (within the group); 1% to the other strain; See notes		
		B	3	4				
*Corollospora gracilis*	19	A	18	3	546	797	14–16%	4%
					17 strains (A) + one strain (B): 0–2% (within A); 3–4% to B; See notes	0%; See notes		
		B	18	3				
*Corollospora intermedia* (**) + *Varicosporina prolifera* (***)	1 + 1; 2 excluded	A	1 + 1	1 + 1	601	431	(**): 11–13%; (***): 17–18%	(**): 2%; (***): 6%
					11%; See notes	4%. See notes		
		B	2 + 1	2 + 1				
*Corollospora lacera*	3	A	0	3	na	793	―	7–9%
					―	One group (two strains) + one strain: 0% (within the group); 3% to the other strain; See notes		
		B	0	2				
*Corollospora luteola* + *Corollospora marina*	3 + 4	A	0 + 1	3 + 3	601	535	17–18%	5–6%
					See notes	0% (within each taxon); 1% (between the two taxa): See notes		
		B	1 + 1	3 + 4				
*Corollospora medi* *terranea*	9	A	9	9	416	793	4–5%	1–2%
					0–1%; See notes	0–1%; See notes		
		B	2	2				
*Corollospora parvula*	10	A	6	7	518	517	16–17%	5–6%
					0%	0%		
		B	6	7				
*Corollospora pseudopulchella*	4	A	1	4	622	536	15–16%: Group 1; ―	5%/8%: Group 1/Group 2
					Remark: only one group has one ITS sequence	Two groups: 0% (within each group); 8% (between groups); See notes		
		B	1	4				
*Corollospora pulchella* + *Clavariopsis bulbosa*	10 + 3 (+2)	A	1 + 1	10 + 3	553	495	22–23%	7%
					1%	Two groups: 0–1% (within each group); 3% (between groups); See notes		
		B	1 + 1	10 + 3				
*Corollospora quinqueseptata*	3	A	0	3	na	703	―	2%
					―	One group + one strain: 0% (within the group); 8% to the other strain; See notes		
		B	0	3				
*Corollospora ramulosa*	5	A	1	5	575	536	17–19%	5–6%
					See notes	0%; See notes		
		B	2	5				
	283		241	114				

Abbreviations: *—Number of positions of the MSA only targeting the *C. maritima* complex. ●—*C. maritima* NBRC 106651. **— *Corollospora intermedia*. ***—*Varicosporina prolifera*. The notes for this table are gathered in Table S2.

**Table 3:** Data retrieved from the global assessments (Files S3 and S4) directed to species formerly assigned to the genus *Corollospora*.

Current Species	Genus/New Genus	New Combinations/(pNSpecies)	Minimum p-Distance				Members Evaluated (nº)	Figure 1, Figure 2 and Figure 3	
			To *C. maritima*		To Species of Other Genera/(*)				
	Name	Type Strain	ITS	28S	ITS	28S	ITS/28S	Clade	
***C. maritima***	*Corollospora*	*C. maritima* (n.d.)	n.a.	n.a.	14%	6%	104/21	A1	11 strains (present study); isolates from lineage 1 [18,25]; sp. 1: strain CMG 52
***C. mediterranea***		*C. mediterranea* MUT 1950	5%	2%	15%	7%	9/9	A2	
***C. quinqueseptata***		*C. quinqueseptata* NBRC 32115	n.d.	2%	n.d.	5%	0/2	A4	Excluding *C. quinqueseptata* PP3909 (present table)
***C. anglusa*** **+ *Varicosporina anglusa***	*Corollosporella* (*)	*Corollosporella anglusa* MF 827	17%	7%	16% (10%)	5% (2%)	1/1	D1	
***C. ramulosa***		*Corollosporella ramulosa* CBS 398.65	16%	7%	16% (9%)	5% (2%)	1/5	D2	Excluding the ITS sequence from the strain NBRC 31325
***C. gracilis***	*Shirahamella* (*)	*Shirahamella gracilis* TKB-C-1457	14%	6%	14% (10%)	5% (2%)	18/3	H	*Shirahamella* sp. 1: strain MD 828
***C. pseudopulchella***	*Ajigaurospora* (*)	*Ajigaurospora pseudopulchella* IFO 32112	15%	6%	15% (8%)	5% (2%)	1/2	G	Excluding the strains NBRC 106641 + NBRC106642 (in the present table)
***C. parvula***	*Garethelia* (*)	*Garethelia parvula* IFO 32112	14%	6%	15% (8%)	5% (3%)	6/7	F	
***C. portsaidica + C. maritima*** **(▪)**	*Corollosporopsis*	*Corollosporopsis portsaidica* MF 832	16%	7%	13%	5%	2 + 58/2 + 2	B1–B6	(▪): distributed by five *Corollosporopsis* spp. Encompasses the lineages 4 and 5 [8,25]
***C. fusca***	*Honshuriella*	*Honshuriella fusca* NBRC 32107	22%	9%	17%	6%	3/3	K1 + K2	*Honshuriella* sp. 1: strain NBRC 32109
***Clavariopsis*** ***bulbosa + C. pulchella***	*Keraliethelia*	*Keraliethelia pulchella* NBRC 31323	24%	9%	20%	10%	2/13	L	*Keraliethelia* sp. 1: four strains NTOU (5730/5732/5738/5741) [20]
***C. filiformis***	*Nakagariella*	*Nakagariella filiformis* CBS 125146 = NBRC 32106 (▪)	20%	9%	19%	8%	1/6	M	(▪): p-distance = 2% between two 28S sequences from two representatives of the type strain
***C. colossa***	*Tokurathelia*	*Tokurathelia colossa* NBRC 32103	n.d.	12%	n.d.	8%	0/1	N	Without ITS data. Assignment due to very high p-distance values in the 28S gene
***C. angusta***	*Paracorollospora*	*Paracorollospora angusta* NBRC 32101	18%	8%	17%	5%	17/18	E1	*Paracorollospora luteola* was included in *Paracorollospora*, based on the type strain and other two strains without ITS sequences. ITS data required to be confirmed genetically different from *Pacorollospora. marina*. Morphologically were accepted as distinct species.
***C. marina***		*Paracorollospora marina* NBRC 103271	18%	6%	17%	4%	1/3	E2	
***C. luteola***		*Paracorollospora luteola* TKBC-1250 = NBRC 31315	n.d..	7%	n.a.	1%	0/3	E3	
***C. maritima* (lineages 2 + 3** [18,25]**)**	pNGenus A	(two pNSpecies)	14%	n.d.	13%	n.d.	11/0	C1 + C2	Only ITS data: sp. 1 and sp. 2 segregating by p-distance = 4%.
***C. colossa***	pNGenus B	(strain NBRC 32105)	23%	11%	18%	8%▪	1/1	J	▪: p-distance from *Tokurathelia colossa*.
***C. colossa***	pNGenus C	(strain NBRC 106650)	24%	11%	19%	9%▪	1/1	I	▪: p-distance from *Tokurathelia colossa*.
***C. cinnamomea***	pNGenus D	(one pNSpecies)	14% ^S^	6%	n.d.	2%	1^S^/4	P	^s^: Short ITS sequence
***C. lacera***	pNGenus E	(one pNSpecies)	n.d	10%	n.d.	7%	0/2	Q	Excluding the strain PP2509 (p-distance = 7% from the other two strains).
***C. pseudopulchella*** **(NBRC 106641/106642)**	pNGenus F	(one pNSpecies)	n.d.	9%	n.d.	7%	0/2	R	p-distance from *Ajigaurospora pseudopulchella*: 9%
***C. quinqueseptata*** **PP3909**	pNGenus G	(one pNSpecies)	n.d.	7%	n.d.	2%	0/1	S	p-distance of 7% from *C. quinqueseptata* and 2% from *Ajigaurospora pseudopulchella*
***C. intermedia*** **NBRC 104402**	controversial placement	*C. intermedia*?	11%	3%	n.d.	4%	1/1	A?	▪: p-distance from *Corollosporella anglusa*.
***Varicosporina prolifera*** **NBRC 32120**	controversial placement	*Corollosporella prolifera*?	17%	6%	n.d.	1%▪	1/1	D?	

pNGenus: putative new genus; pNSpecies: putative new species; between parenthesis in columns 6 and 7: p-distances between species of the four genera flagged with (*); n.a.: not applicable; n.d.: not determined. Note: other flags (e.g.: ▪, ^S^) are explained in the Remarks concerning the specific situation.

**Table 4:** Morphological data and type species ^(1)^ from the new genera assigned in the present study.

					Ascospores Septa				Appendages Primary		Secondary		
		Asci Diameter (µm)	Texture	Peridium (nº of Layers)	Type	Number	Dimension * (µm)	Pigmentation	Polar Spines	Dimension * (µm)	Equatorial	Polar	Reference
Genus	Type Species										Dimension * (µm)	Dimension * (µm)	
*Ajigaurospora*	*Ajigaurospora pseudopulchella*	180–245	Carbonaceous	2	Transverse	7–11	65–97.5 × 7.5–11.5	Hyaline	Absent/attenuate apices	–	18–31	7.5–12.5	[4,6]
*Corollospora*	*Corollospora maritima*	90–400	Carbonaceous	2	Transverse	1	20–34(53) × (4−)6–11 (−14)	Hyaline	Present	7–17.5 (23) × 1–1.5	5–16 (−20) × 1	8–10	[1,2,3,4],
*Corollosporella*	*Corollosporella anglusa*	60–110	Coriaceous	2	Transverse	1	18–30 × 3–4 (−5)	Hyaline	Present	4–7 × 1–2	10–12	4–6	[5]
*Corollosporopsis*	*Corollosporopsis portsaidica*	80–130	Carbonaceous	1	Transverse	1	27–32 × 8–9	Hyaline to brown	Present	14–18 × 1.5–2	10–12	7–10	[5]
*Honshuriella*	*Honshuriella fusca*	264–440	Carbonaceous	3	Transverse and Longitudinal	(5−) 12–21	63–220 × 20–38	Dark brown	Present	28.5–65	25–75	28–54	[6]
*Keraliethelia*	*Keraliethelia* *pulchella*	80–325	Carbonaceous	2	Transverse	7 (9–13)	52.5–102.5 × 7–12(−16)	Hyaline	Absent/conical papilla at each end	−	15–29 × 1	12.5–20 (−24) × 1	[1,2,3,4]
*Nakagiriella*	*Nakagiriella* *filiformis*	243–418	Carbonaceous	3	Transverse	13 (9–17)	(73–) 87–120 × 5–8(−10)	Hyaline	Absent	−	13–22	18–25	[6]
*Paracorollospora*	*Paracorollospora angusta*	60–245	Carbonaceous	2	Transverse	3(–5)	35–57 × 3–7.5	Hyaline	Present	2–8	18–24.5	5.8–12.5	[6]
*Shirahamella*	*Shirahamella gracilis*	88–185	Carbonaceous	2	Transverse	1	26–45 × 3–5.5(−7)	Hyaline	Present	6.5–12	12–20	4–8	[6]
*Tokurathelia*	*Tokurathelia colossa*	318–515	Carbonaceous	3	Transverse	(6–)7 (−8)	60–108 × 13–26	Hyaline	Absent	−	20–28	20–27	[6]

^(1)^: for type species based on the sexual morph; *: length or length × width; –: information not available.

## Data Availability

The Sanger sequences generated in this study are available in GenBank, NCBI. The MSAs and pairwise distance data are available in Files S1–S4.

## Supplementary Materials

The following supporting information can be downloaded at: https://www.mdpi.com/2309-608X/9/8/841/s1, Table S1: Annotated sequence data from the sequences retrieved for the present study. Table S2: Notes to Table 2 and Table S1. File S1: Sectorial (one taxon) MSAs of ITS and 28S sequences to separately assess the members of each species, together with representatives of *Corollospora maritima sensu stricto*. File S2: Annotated pairwise distance matrices from the ITS and 28S sectorial MSAs to separately assess the members of each species, together with representatives of *Corollospora maritima sensu stricto*. File S3: Global MSAs from the ITS, 28S and 18S regions and concatenated 18S/ITS/28S MSA, to assess taxonomic placements of selected species formerly assigned to the genus *Corollospora*. File S4: Annotated pairwise distance matrices from global MSAs involving the ITS, 28S, and 18S regions, to assess taxonomic placements of selected species formerly assigned to the genus *Corollospora*.

## Abbreviations

Table S1: NBRC—Biological Resource Centre, NITE (National Institute of Technology and Evaluation); NCBI—National Center for Biotechnology Information (National Institutes of Health); UNITE—https://unite.ut.ee/; *—Type strain; (a)—sequence size under the minimum accepted length; (b)—issues that reduce the length of MSAs; (c)—Sequence that possibly does not fully match with the registered region; (d)—sequence possibly not representative of the species recorded; (e)—Sequence without updated taxon in NCBI; FBC—Sequence validated by the Fungal Barcoding Consortium; AFToL—Sequence validated by AFToL (Assembling the Fungal Tree of Life); UNITE—sequence retrieved from the UNITE database; Notes: Dash (–) indicates missing data. Access numbers and isolate names underlined indicate participation in the global evaluation of p-distance and phylogenetic analyses.
