# Correction: Correia et al. Redefining the Genus *Corollospora* Based on Morphological and Phylogenetic Approaches. *J. Fungi* 2023, *9*, 841

**DOI:** 10.3390/jof10010039

**Published:** 2024-01-04

**Authors:** Pedro Correia, Egídia Azevedo, Maria F. Caeiro

**Affiliations:** 1 Centro de Ecologia, Evolução e Alterações Climáticas (ce3c), Faculdade de Ciências da Universidade de Lisboa (FCUL), DBV, C2, Campo Grande, 1749-016 Lisboa, Portugal; 2 Centro de Estudos do Ambiente e do Mar (CESAM Lisboa), Faculdade de Ciências da Universidade de Lisboa (FCUL), DBV, C2, Campo Grande, 1749-016 Lisboa, Portugal

This correction refers to two new genera (*Corollosporella* and *Corollosporopsis*) and three new species combinations published in *Journal of Fungi* [1], which are invalid in MycoBank due to incorrect indication of the pages where the basionyms of the corresponding type species were validly published. Therefore, new submissions to MycoBank had to be performed since the originally issued MB numbers must remain with the invalid names.

The correction below presents the new MycoBank numbers issued to these new taxa and the rectifications introduced in their descriptions. This correction has no other implications in the results and detailed descriptions contained in the previous publication [1].

In Section 4.2. New Genera and New Combinations, from “***Corollosporella* E. Azevedo, P. Correia & M.F. Caeiro gen. nov.**” to “**Synnonym:** *Varicosporina ramulosa* Meyers & Kohlm., Can. J. Bot. 43: 916. 1965.” should be corrected to read as follows:

***Corollosporella* E. Azevedo, P. Correia & M.F. Caeiro gen. nov.**

**MycoBank MB851370.**

**Etymology:** Morphological characters resembling *Corollospora*.

Saprobic. **Sexual morph:** *Ascomata* solitary or gregarious, superficial, ostiolate, papillate, black, coriaceous, or carbonaceous. The venter of immature ascomata is filled by thick-walled, hyaline roundish pseudoparenchymatous cells deliquescent, with pitted walls, deliquescent. *Peridium* with thick-walled cells, brown, composed of two layers: polygonal, roundish cells on the outer layer and flat cells on the inner layer. Asci 8-spored, ellipsoidal, unitunicate, early deliquescing. *Ascospores* fusiform to ellipsoidal, 1-septate, constricted at the central septum, hyaline. *Primary appendages* present at each end of the spore; secondary appendages are apical and equatorial (peritrichous, around the central septa), formed by fragmentation and peeling of the exospore [12–14] **Asexual morph**: *Conidiophores* simple or branched, multiseptate, hyaline. *Conidiogenous cells* proliferating, sympodial at the apex or monoblastic. *Conidia* septate, hyaline, branched, filamentous, which disarticulate into small segments; or conidia consisting in a system of axes; a main axis with two, rarely three side branches, each side branch arising from the previously developed branch [13,68].

**Type species**: *Corollosporella anglusa* (Abdel-Wahab & Nagah.) E. Azevedo, P. Correia & M.F. Caeiro comb. nov.

**New combinations:**

1. ***Corollosporella anglusa*** **(Abdel-Wahab & Nagah.) E. Azevedo, P. Correia & M.F. Caeiro comb. nov. MycoBank MB851371.**

**Basionym:** *Corollospora anglusa* Abdel-Wahab & Nagah., Mycoscience 50(3): 149. 2009.

**Synonyms:**

*Varicosporina anglusa* Abdel-Wahab & Nagah., Mycoscience 50(3): 150. 2009.

*Corollosporella anglusa* (Abdel Wahab & Nagah.) E. Azevedo, P. Correia & M.F. Caeiro, J. Fungi 9 (8, no. 841): 26 (2023), nom. inval., Art. 41.5 (Shenzhen).

**Distribution:** Egypt (Abdel-Wahab et al. 2009).

**Holotype:** IMI 395681, **Type strain:** MF 827 (=NBRC 104919).

2. ***Corollosporella ramulosa*** **(Meyers & Kohlm.) E. Azevedo, P. Correia & M.F. Caeiro comb. nov. MycoBank MB851372.**

**Basionym:** *Varicosporina ramulosa* Meyers & Kohlm., Can. J. Bot. 43: 916. 1965.

**Synonyms:**

*Corollospora ramulosa* (Meyers & Kohlm.) E.B.G. Jones & Abdel-Wahab, in Réblová et al., IMA Fungus 7(1): 137. 2016.

*Corollosporella ramulosa* (Meyers & Kohlm.) E. Azevedo, P. Correia & M.F. Caeiro, J. Fungi 9 (8, no. 841): 26 (2023), nom. inval., Art. 35.1 (Shenzhen).

In Section 4.2. New Genera and New Combinations, from “***Corollosporopsis* M.F. Caeiro, P. Correia & E. Azevedo gen. nov.**” to “**Basiomyn:** *Corollospora portsaidica* Abdel-Wahab and Nagahama, Mycoscience 50: 147–155. 2009.” should be corrected to read as follows:

***Corollosporopsis* M.F. Caeiro, P. Correia & E. Azevedo gen. nov.**

**MycoBank MB851352.**

**Etymology:** Reference to the morphological similarity with Corollospora.

Saprobic. **Sexual morph:** *Ascomata* solitary, superficial, globose, ostiolate, papillate, black, carbonaceous. Pseudoparenchymatous thick-walled cells, polygonal, with pit connections in their walls, fill the centrum of the immature fruit body, deliquescing. *Asci* 8-spored, broadly fusoid, unitunicate, early deliquescing. *Ascospores* 1-septate, fusiform, hyaline or brown, smooth-walled, one-septate, constricted at the central septum. Primary appendages are single, terminal at each end of the spore, spine, or thorn-like; secondary appendages developed by the fragmentation and peeling of the exospore, equatorial double frill or ribbon-like, polar forming a tube or sheets (adapted from [13,15,18,25]). **Asexual morph:** Undetermined.

**Type species**: *Corollosporopsis portsaidica* (Abdel-Wahab & Nagah.) M.F. Caeiro, P. Correia & E. Azevedo comb. nov.

New combination:

***Corollosporopsis portsaidica* (Abdel-Wahab & Nagah.) M.F. Caeiro, P. Correia & E. Azevedo comb. nov. MycoBank MB851353.**

**Basionym**: *Corollospora portsaidica* Abdel-Wahab & Nagah., Mycoscience 50: 152. 2009.

**Synonym:** *Corollosporopsis portsaidica* (Abdel-Wahab & Nagah.) M.F. Caeiro, P. Correia & E. Azevedo, J. Fungi 9 (8, no. 841): 27 (2023), nom. inval., Art. 41.5 (Shenzhen).

Additional corrections: in the Abstract and in the fourth-to-last paragraph of the Conclusions, the names of two new combinations with typos should be corrected to read as *Keraliethelia pulchella* and *Tokurathelia colossa*.

All co-authors agree with the content of this correction and wish to apologize to readers for any inconvenience resulting from this error.

The authors state that the scientific conclusions are unaffected. This correction was approved by the Academic Editor. The original publication has also been updated.

## References

[1] Correia P., Azevedo E., Caeiro M.F. (2023). Redefining the Genus *Corollospora* based on morphological and phylogenetic approaches. J. Fungi.

