# Supplementary material for: Redefining the Genus Corollospora Based on Morphological and Phylogenetic Approaches
Source: J Fungi (Basel). 2023 Aug 11;9(8):841. doi: 10.3390/jof9080841 (PMC10455288; doi:10.3390/jof9080841)
Supplement: Supplementary file 1 [file File S4_(p-distances_28S gene).pdf › File S4_PDFs/File S4_(p-distances_28S gene).pdf]

This analysis involved 55 nucleotide sequences of the 28S gene.
There were a total of 503 positions in the final dataset.

p-distance thresholds:

same species: 0–1%. Exception (confliting result; 2%): two sequences of the same organism (the type strain of *Nakagariella filiformis*)*
same genus: ≤4%. Exception: *C. mediterranea* and *C. intermedia* (5%)
between genera: ≥5%. Exceptions: **2–4%**
to the outgroups: 8–17%; 23–31%
between the species of new genera and *C. maritima* : ≥6%
according to evaluations of the ITS region [yellow]
conflicting results [orange]

		2	3	4	5	6	7	8	9	10	11	12	13	14	15	16	17	18	19	20	21	22	23	24	25	26	27	28	29	30	31	32	33	34	35	36	37	38	39	40	41	42	43	44	45	46	47	48	49	50	51	52	53	54	55	56
2	*Corollospora _maritima* _FCUL280207CF8																																																							
3	*Corollospora _maritima* _FCUL_CaC1	0.00																																																					
4	*Corollospora _maritima* _NBRC 32117_(JN941492)	0.00	0.00																																																				
5	*Corollospora* _sp._CMG_52_(MT232383)	0.00	0.00	0.00																																																		
6	*Corollospora _mediterranea* _MUT 5040_(MW584958)	0.02	0.02	0.02	0.02																																																
7	*Corollospora _mediterranea* _MUT 5048_(MW584959)	0.02	0.02	0.02	0.02	0.00																																														
8	*Corollospora _quinqueseptata* _NBRC_32114_(JN941498)	0.02	0.03	0.02	0.02	0.04	0.04																																													
9	*Corollospora _quinqueseptata* _NBRC_32115_(JN941499)	0.02	0.03	0.02	0.02	0.04	0.04	0.00																																												
10	? *Corollospora _intermedia* _NBRC 104402	0.03	0.03	0.03	0.03	0.05	0.05	0.02	0.02	*	***Corollospora intermedia***																																													
11	*Corollosporopsis* _sp._3_NBRC_32118_(JN941493)	0.08	0.09	0.08	0.08	0.09	0.09	0.08	0.08	0.07																																										
12	*Corollosporopsis _portsaidica* _IMI 395684_(NG078629)	0.08	0.09	0.08	0.08	0.09	0.09	0.08	0.08	0.07	0.00																																									
13	*Corollosporopsis _portsaidica* _MD 1301_(KJ406564)	0.08	0.09	0.08	0.08	0.09	0.09	0.08	0.08	0.07	0.00	0.00																																								
14	*Corollosporopsis* _sp._5_NBRC_106651	0.07	0.07	0.07	0.07	0.08	0.08	0.06	0.06	0.06	0.03	0.03	0.03																																							
15	pNGenus D_NBRC_32125_(JN941479)	0.06	0.06	0.06	0.06	0.07	0.07	0.05	0.05	0.05	0.06	0.06	0.06	0.07		***Corollospora cinnamomea***																																								
16	pNGenus D_NBRC_32126_(JN941480)	0.06	0.06	0.06	0.06	0.07	0.07	0.05	0.05	0.05	0.06	0.06	0.06	0.07	0.00		***Corollospora cinnamomea***																																						
17	*Corollosporella _anglusa* _NBRC_104919	0.07	0.07	0.07	0.07	0.08	0.08	0.05	0.05	0.05	0.05	0.05	0.05	0.05	**0.02**	**0.02**																																								
18	*Corollosporella _ramulosa* _CBS:_398.65 (MH870274)	0.07	0.07	0.07	0.07	0.08	0.08	0.06	0.06	0.05	0.07	0.07	0.07	0.07	**0.02**	**0.02**	0.02																																						
19	? *Corollosporella prolifera* _NBRC 32120	0.06	0.06	0.06	0.06	0.07	0.07	0.05	0.05	**0.04**	0.06	0.06	0.06	0.06	**0.02**	**0.02**	**0.01**	**0.02**	*	***Varicosporina prolifera***																																				
20	*Shirahamella _gracilis* _NBRC_32111 (JN941487)	0.06	0.06	0.06	0.06	0.07	0.07	0.05	0.05	0.05	0.07	0.07	0.07	0.06	**0.02**	**0.02**	**0.02**	**0.03**	**0.02**																																				
21	*Shirahamella* _sp._MD_828_(AB361019)	0.06	0.06	0.06	0.06	0.07	0.07	0.05	0.05	0.05	0.07	0.07	0.07	0.06	**0.02**	**0.02**	**0.02**	**0.03**	**0.02**	0.00																																			
22	*Ajigaurospora _pseudopulchella* _NBRC_32112_(JN941494)	0.06	0.06	0.06	0.06	0.07	0.07	0.05	0.05	**0.04**	0.06	0.06	0.06	0.06	**0.02**	**0.02**	**0.03**	**0.03**	**0.03**	**0.02**	**0.02**																																		
23	*Ajigaurospora _pseudopulchell* a_NBRC_32113_(JN941495)	0.06	0.06	0.06	0.06	0.07	0.07	0.05	0.05	**0.04**	0.06	0.06	0.06	0.06	**0.02**	**0.02**	**0.03**	**0.03**	**0.03**	**0.02**	**0.02**	0.00																																	
24	pNGenus G_PP3909_(AF491262)	0.07	0.07	0.07	0.07	0.08	0.08	0.07	0.07	0.06	0.08	0.08	0.08	0.08	**0.04**	**0.04**	**0.04**	0.05	**0.04**	**0.04**	**0.04**	**0.02**	**0.02**		***Corollospora quinqueseptata***																															
25	*Garethelia _parvula* _IFO 32159_(DQ104810)	0.06	0.06	0.06	0.06	0.07	0.07	0.05	0.05	0.05	0.07	0.07	0.07	0.07	**0.03**	**0.03**	**0.03**	**0.04**	**0.03**	**0.03**	**0.03**	**0.03**	**0.03**	0.05																																
26	*Garethelia _parvula* _NBRC 32160	0.06	0.06	0.06	0.06	0.07	0.07	0.05	0.05	0.05	0.07	0.07	0.07	0.07	**0.03**	**0.03**	**0.03**	**0.04**	**0.03**	**0.03**	**0.03**	**0.03**	**0.03**	0.05	0.00																															
27	*Paracorollospora _angusta* _NBRC_32101 (JN941477)	0.08	0.08	0.08	0.08	0.10	0.10	0.08	0.08	0.06	0.08	0.08	0.08	0.09	0.05	0.05	0.06	0.06	0.06	0.06	0.06	0.06	0.06	0.07	0.07	0.07																														
28	*Paracorollospora _angusta* _NBRC_32102_(JN941478)	0.08	0.08	0.08	0.08	0.10	0.10	0.08	0.08	0.06	0.08	0.08	0.08	0.09	0.05	0.05	0.06	0.06	0.06	0.06	0.06	0.06	0.06	0.07	0.07	0.07	0.00																													
29	*Paracorollospora _marina* _NBRC 103271	0.07	0.07	0.07	0.07	0.08	0.08	0.07	0.07	0.06	0.08	0.08	0.08	0.08	**0.04**	**0.04**	0.06	0.05	0.05	0.05	0.05	0.05	0.05	0.07	0.06	0.06	0.02	0.02																												
30	*Paracorollospora _marina* _AFTOL-_ID_5008 (FJ176898)	0.07	0.07	0.07	0.07	0.07	0.08	0.07	0.07	0.06	0.07	0.07	0.07	0.07	**0.04**	**0.04**	**0.04**	0.05	0.05	**0.04**	**0.04**	0.05	0.05	0.06	0.05	0.05	0.02	0.02	0.00																											
31	?*Paracorollospora luteola* _NBRC_31315_(JN941490)	0.07	0.07	0.07	0.07	0.08	0.08	0.06	0.06	0.05	0.07	0.07	0.07	0.07	**0.04**	**0.04**	0.05	0.05	0.05	**0.04**	**0.04**	**0.04**	**0.04**	0.06	0.05	0.05	0.03	0.03	0.01	0.01		***Corollospora luteola***																								
32	?*Paracorollospora luteola* _NBRC_31316_(JN941491)	0.07	0.07	0.07	0.07	0.08	0.08	0.06	0.06	0.05	0.07	0.07	0.07	0.07	**0.04**	**0.04**	0.05	0.05	0.05	**0.04**	**0.04**	**0.04**	**0.04**	0.06	0.05	0.05	0.03	0.03	0.01	0.01	0.00		***Corollospora luteola***																							
33	*Keraliethelia _pulchella* _NBRC_32123 (JN941496)	0.10	0.10	0.10	0.10	0.11	0.11	0.11	0.11	0.11	0.14	0.14	0.14	0.14	0.11	0.11	0.11	0.11	0.10	0.11	0.11	0.11	0.11	0.12	0.11	0.11	0.13	0.13	0.12	0.10	0.11	0.11																								
34	*Keraliethelia _pulchella*_ NBRC 31323	0.10	0.10	0.10	0.10	0.11	0.11	0.11	0.11	0.11	0.14	0.14	0.14	0.14	0.11	0.11	0.11	0.11	0.10	0.11	0.11	0.11	0.11	0.12	0.11	0.11	0.13	0.13	0.12	0.10	0.11	0.11	0.00																							
35	*Keraliethelia _pulchella* _NTOU5725_(OQ130070)	0.09	0.09	0.09	0.09	0.10	0.11	0.11	0.11	0.11	0.14	0.14	0.14	0.13	0.10	0.10	0.11	0.11	0.10	0.10	0.10	0.11	0.11	0.12	0.11	0.11	0.12	0.12	0.11	0.10	0.11	0.11	0.01	0.01																						
36	*Keraliethelia_pulcehlla* _NTOU5727_(OQ130071)	0.09	0.10	0.09	0.09	0.11	0.11	0.11	0.11	0.11	0.14	0.14	0.14	0.13	0.11	0.11	0.11	0.11	0.10	0.10	0.10	0.11	0.11	0.12	0.11	0.11	0.13	0.13	0.12	0.10	0.11	0.11	0.01	0.01	0.00																					
37	*Keraliethelia* _sp.1_NTOU5732_(OQ130075)	0.10	0.10	0.10	0.10	0.11	0.12	0.12	0.12	0.11	0.15	0.15	0.15	0.14	0.11	0.11	0.12	0.12	0.11	0.11	0.11	0.12	0.12	0.13	0.12	0.12	0.13	0.13	0.12	0.11	0.12	0.12	0.04	0.04	0.03	0.03																				
38	*Keraliethelia* _sp.1_NTOU5730_(OQ130076)	0.10	0.10	0.10	0.10	0.11	0.12	0.12	0.12	0.11	0.15	0.15	0.15	0.14	0.11	0.11	0.12	0.12	0.11	0.11	0.11	0.12	0.12	0.13	0.12	0.12	0.13	0.13	0.12	0.11	0.12	0.12	0.04	0.04	0.03	0.03	0.00																			
39	*Honshuriella _fusca* _NBRC_32107_(JN941483)	0.08	0.09	0.08	0.08	0.10	0.10	0.08	0.08	0.07	0.09	0.09	0.09	0.08	0.07	0.07	0.07	0.07	0.07	0.06	0.06	0.07	0.07	0.09	0.08	0.08	0.08	0.08	0.07	0.07	0.06	0.06	0.13	0.13	0.13	0.13	0.13	0.13																		
40	*Honshuriella _fusca* _NBRC_32108_(JN941484)	0.08	0.09	0.08	0.08	0.10	0.10	0.08	0.08	0.07	0.09	0.09	0.09	0.08	0.07	0.07	0.07	0.07	0.07	0.06	0.06	0.07	0.07	0.09	0.08	0.08	0.08	0.08	0.07	0.07	0.06	0.06	0.13	0.13	0.13	0.13	0.13	0.13	0.00																	
41	*Honshuriella* _sp.1_NBRC_32109_(JN941485)	0.09	0.09	0.09	0.09	0.11	0.11	0.08	0.08	0.07	0.10	0.10	0.10	0.09	0.09	0.09	0.08	0.08	0.07	0.08	0.08	0.09	0.09	0.11	0.10	0.10	0.09	0.09	0.08	0.07	0.07	0.07	0.13	0.13	0.12	0.13	0.13	0.13	0.02	0.02																
42	*Nakagariella _filiformi* s_CBS_125146_(MH874958)*	0.10	0.11	0.10	0.10	0.10	0.10	0.11	0.11	0.12	0.15	0.15	0.15	0.14	0.09	0.09	0.10	0.10	0.10	0.10	0.10	0.11	0.11	0.12	0.11	0.11	0.14	0.14	0.13	0.12	0.12	0.12	0.11	0.11	0.10	0.10	0.11	0.11	0.14	0.14	0.15															
43	*Nakagariella _filiformis* _NTOU5714_(OQ130067)	0.09	0.09	0.09	0.09	0.09	0.09	0.10	0.10	0.10	0.13	0.13	0.13	0.12	0.07	0.07	0.09	0.08	0.08	0.08	0.08	0.09	0.09	0.10	0.09	0.09	0.12	0.12	0.11	0.10	0.10	0.10	0.09	0.09	0.08	0.09	0.09	0.09	0.12	0.12	0.13	0.02														
44	*Nakagariella _filiformis* _NTOU5712_(OQ130066)	0.09	0.09	0.09	0.09	0.09	0.09	0.10	0.10	0.10	0.13	0.13	0.13	0.12	0.07	0.07	0.09	0.08	0.08	0.08	0.08	0.09	0.09	0.10	0.09	0.09	0.12	0.12	0.11	0.10	0.10	0.10	0.09	0.09	0.08	0.09	0.09	0.09	0.12	0.12	0.13	0.02	0.00													
45	*Nakagariella _filiformis* _NBRC 32106*	0.09	0.09	0.09	0.09	0.09	0.08	0.10	0.10	0.10	0.13	0.13	0.13	0.12	0.08	0.08	0.09	0.08	0.09	0.09	0.09	0.09	0.09	0.10	0.09	0.09	0.12	0.12	0.11	0.10	0.10	0.10	0.09	0.09	0.09	0.09	0.09	0.09	0.12	0.12	0.13	0.02	0.01	0.01												
46	pNGenus_B_NBRC_32105_(JN941482)	0.11	0.12	0.11	0.11	0.13	0.13	0.11	0.11	0.10	0.14	0.14	0.14	0.13	0.10	0.10	0.11	0.11	0.10	0.09	0.09	0.10	0.10	0.11	0.10	0.10	0.11	0.11	0.10	0.09	0.10	0.10	0.11	0.11	0.11	0.11	0.11	0.11	0.11	0.11	0.10	0.15	0.12	0.12	0.13		***Corollospora colossa***									
47	pNGenus_C_NBRC_106650	0.11	0.11	0.11	0.11	0.13	0.13	0.12	0.12	0.11	0.15	0.15	0.15	0.14	0.11	0.11	0.12	0.12	0.11	0.11	0.11	0.12	0.12	0.13	0.11	0.11	0.12	0.12	0.10	0.10	0.11	0.11	0.12	0.12	0.11	0.12	0.12	0.12	0.12	0.12	0.11	0.15	0.13	0.13	0.13	0.03		***Corollospora colossa***								
48	*Tokuratelia _colossa* _NBRC_32103_(JN941481)	0.12	0.12	0.12	0.12	0.13	0.13	0.12	0.12	0.10	0.12	0.12	0.12	0.12	0.10	0.10	0.10	0.11	0.10	0.10	0.10	0.09	0.09	0.10	0.10	0.10	0.12	0.12	0.12	0.11	0.10	0.10	0.12	0.12	0.12	0.12	0.13	0.13	0.12	0.12	0.13	0.14	0.12	0.12	0.12	0.08	0.09									
49	pNGenus E_NBRC_32121_(JN941488)	0.10	0.10	0.10	0.10	0.11	0.12	0.10	0.10	0.09	0.11	0.11	0.11	0.10	0.09	0.09	0.09	0.09	0.09	0.09	0.09	0.10	0.10	0.12	0.10	0.10	0.10	0.10	0.08	0.08	0.07	0.07	0.11	0.11	0.10	0.11	0.09	0.09	0.09	0.09	0.10	0.13	0.11	0.11	0.11	0.14	0.14	0.13		***Corollospora lacera***						
50	pNGenus E_NBRC_32122_(JN941489)	0.10	0.10	0.10	0.10	0.11	0.12	0.10	0.10	0.09	0.11	0.11	0.11	0.10	0.09	0.09	0.09	0.09	0.09	0.09	0.09	0.10	0.10	0.12	0.10	0.10	0.10	0.10	0.08	0.08	0.07	0.07	0.11	0.11	0.10	0.11	0.09	0.09	0.09	0.09	0.10	0.13	0.11	0.11	0.11	0.14	0.14	0.13	0.00		***Corollospora lacera***					
51	pNGenus F_NBRC 106641	0.09	0.09	0.09	0.09	0.11	0.11	0.10	0.10	0.08	0.11	0.11	0.11	0.11	0.07	0.07	0.08	0.08	0.08	0.09	0.09	0.09	0.09	0.10	0.09	0.09	0.09	0.09	0.09	0.08	0.08	0.08	0.12	0.12	0.12	0.12	0.11	0.11	0.09	0.09	0.09	0.13	0.11	0.11	0.11	0.11	0.11	0.11	0.10	0.10		***Corollospora pseudopulchella***				
52	pNGenus F_NBRC 106642	0.09	0.09	0.09	0.09	0.11	0.11	0.10	0.10	0.08	0.11	0.11	0.11	0.11	0.07	0.07	0.08	0.08	0.08	0.09	0.09	0.09	0.09	0.10	0.09	0.09	0.09	0.09	0.09	0.08	0.08	0.08	0.12	0.12	0.12	0.12	0.11	0.11	0.09	0.09	0.09	0.13	0.11	0.11	0.11	0.11	0.11	0.11	0.10	0.10	0.00		***Corollospora pseudopulchella***			
53	*Cucullosporella _mangrovei* _NBRC_32477	0.10	0.11	0.10	0.10	0.11	0.11	0.10	0.10	0.09	0.10	0.10	0.10	0.09	0.11	0.11	0.10	0.10	0.10	0.10	0.10	0.11	0.11	0.11	0.11	0.11	0.12	0.12	0.10	0.10	0.10	0.10	0.15	0.15	0.15	0.15	0.15	0.15	0.11	0.11	0.12	0.15	0.13	0.13	0.14	0.17	0.17	0.15	0.12	0.12	0.14	0.14				
54	*Cucullosporella _mangrovei* _NBRC_105272	0.10	0.10	0.10	0.10	0.11	0.11	0.10	0.10	0.09	0.10	0.10	0.10	0.09	0.11	0.11	0.10	0.10	0.10	0.10	0.10	0.11	0.11	0.11	0.11	0.11	0.12	0.12	0.10	0.09	0.10	0.10	0.15	0.15	0.15	0.15	0.15	0.15	0.11	0.11	0.11	0.15	0.13	0.13	0.14	0.16	0.17	0.15	0.12	0.12	0.14	0.14	0.00			
55	*Trailia _ascophylli* _NBRC_106638	0.26	0.26	0.26	0.26	0.27	0.27	0.27	0.27	0.25	0.23	0.23	0.23	0.25	0.26	0.26	0.25	0.25	0.25	0.25	0.25	0.26	0.26	0.27	0.26	0.26	0.26	0.26	0.26	0.24	0.26	0.26	0.28	0.28	0.28	0.29	0.28	0.28	0.26	0.26	0.26	0.31	0.29	0.29	0.29	0.27	0.29	0.27	0.27	0.27	0.26	0.26	0.27	0.27		
56	*Trailia _ascophyll* i_NBRC_106637	0.26	0.26	0.26	0.26	0.27	0.27	0.27	0.27	0.25	0.23	0.23	0.23	0.25	0.26	0.26	0.25	0.25	0.25	0.25	0.25	0.26	0.26	0.27	0.26	0.26	0.26	0.26	0.26	0.23	0.26	0.26	0.28	0.28	0.28	0.29	0.28	0.28	0.25	0.25	0.26	0.31	0.29	0.29	0.29	0.27	0.29	0.27	0.27	0.27	0.26	0.26	0.26	0.26	0.00	

Legend:
species of Putative New genera — **current identification**
(not subjected to a MAS for ITS analysis)
