# Supplementary material for: Redefining the Genus Corollospora Based on Morphological and Phylogenetic Approaches
Source: J Fungi (Basel). 2023 Aug 11;9(8):841. doi: 10.3390/jof9080841 (PMC10455288; doi:10.3390/jof9080841)
Supplement: Supplementary file 1 [file jof-09-00841-s001.zip › File S4_PDFs/File S4_(p-distances_18S gene).pdf]

		2	3	4	5	6	7	8	9	10	11	12	13	14	15	16	17	18	19	20	21	22	23	24	25
2	*C._maritima* _NBRC_32117_(JN941654)																								
3	*C._mediterranea_* MUT_5040_(MW584967)	**0.00**																							
4	*C._mediterranea* _MUT_5048_(MW584968)	**0.00**	**0.00**																						
5	*C._quinqueseptata_* NBRC_32115_(JN941647)	0.02	0.02	0.02																					
6	C._quinqueseptata_NBRC_32114_(JN941648)	0.02	0.02	0.02	0.00																				
7	*C._intermedia* _NBRC_104402	0.01	0.01	0.01	0.01	0.01																			
8	*Corollosporopsis_* sp._3_NBRC_32118_(JN941653)	0.03	0.03	0.03	0.03	0.03	0.02																		
9	pNGenus_D_NBRC_32125_(JN941666)	0.02	0.02	0.02	0.02	0.02	0.01	0.02																	
10	pNGenus_D_NBRC_32126_(JN941665)	0.02	0.02	0.02	0.02	0.02	0.01	0.02	0.00																
11	*Shirahamella_gracilis* _NBRC_32111_(JN941659)	0.01	0.02	0.02	0.01	0.01	0.01	0.02	0.01	0.01															
12	*Shirahamella_gracilis* _NBRC_32110_(JN941660)	0.01	0.02	0.02	0.01	0.01	0.01	0.02	0.01	0.01	0.00														
13	*Ajigaurospora_pseudopulchella* _NBRC_32113_(JN941651)	0.02	0.02	0.02	0.02	0.02	0.01	0.02	**0.00**	**0.00**	0.01	0.01													
14	*Ajigaurospora_pseudopulchella* _NBRC_32112_(JN941652)	0.02	0.02	0.02	0.02	0.02	0.01	0.02	**0.00**	**0.00**	0.01	0.01	0.00												
15	*Garethelia_parvula* _IFO32159_(DQ104806)	0.02	0.02	0.02	0.02	0.02	0.01	0.02	0.01	0.01	**0.00**	**0.00**	0.01	0.01											
16	*Paracorollospora_angusta* _NBRC_32101_(JN941668)	0.02	0.02	0.02	0.02	0.02	0.02	0.03	0.01	0.01	0.01	0.01	0.01	0.01	0.01										
17	*Paracorollospora_angusta_* NBRC_32102_(JN941667)	0.02	0.02	0.02	0.02	0.02	0.02	0.03	0.01	0.01	0.01	0.01	0.01	0.01	0.01	0.00									
18	*Paracorollospora_luteola* _NBRC_31316_(JN941655)	0.02	0.03	0.03	0.02	0.02	0.01	0.02	0.01	0.01	0.01	0.01	0.01	0.01	0.01	0.01	0.01								
19	*Paracorollospora_luteola* _NBRC_31315_(JN941656)	0.02	0.03	0.03	0.02	0.02	0.01	0.02	0.01	0.01	0.01	0.01	0.01	0.01	0.01	0.01	0.01	0.00							
20	*Keraliethelia_pulchella* _NBRC_32123_(JN941650)	0.03	0.03	0.03	0.03	0.03	0.02	0.03	0.02	0.02	0.02	0.02	0.02	0.02	0.02	0.02	0.02	0.02	0.02						
21	*Honshuriella_fusca_* NBRC_32107_(JN941662)	0.03	0.03	0.03	0.03	0.03	0.02	0.03	0.02	0.02	0.02	0.02	0.02	0.02	0.02	0.02	0.02	0.02	0.02	0.03					
22	pNGenus_B_NBRC_32105_(JN941663)	0.03	0.03	0.03	0.02	0.02	0.02	0.03	0.01	0.01	0.02	0.02	0.01	0.01	0.02	0.02	0.02	0.02	0.02	0.02	0.03				
23	pNGenus_E_NBRC_32121_(JN941658)	0.02	0.03	0.03	0.03	0.03	0.02	0.03	0.02	0.02	0.02	0.02	0.02	0.02	0.02	0.01	0.01	0.02	0.02	0.02	0.02	0.02			
24	pNGenus_E_NBRC_32122_(JN941657)	0.02	0.03	0.03	0.03	0.03	0.02	0.03	0.02	0.02	0.02	0.02	0.02	0.02	0.02	0.01	0.01	0.02	0.02	0.02	0.02	0.02	0.00		
25	*Cucullosporella_mangrovei* _NTOU3744_(KX686771)	0.04	0.04	0.04	0.04	0.04	0.04	0.04	0.03	0.03	0.03	0.03	0.03	0.03	0.03	0.03	0.03	0.03	0.03	0.04	0.04	0.04	0.03	0.03	

This analysis involved 24 nucleotide sequences of the 18S gene.
There were a total of 839 positions in the final dataset.

p-distance thresholds:

same species: 0%
same genus: 0–2%
between genera: ≥1%. Exceptions: **0%** (*Shirahamella* and *Garethelia*; *Ajigaurospora* and pNGenus D)
to the outgroup: 3–4%
between the species of new genera and *C. maritima*: 1–3%
