# Supplementary material for: Redefining the Genus Corollospora Based on Morphological and Phylogenetic Approaches
Source: J Fungi (Basel). 2023 Aug 11;9(8):841. doi: 10.3390/jof9080841 (PMC10455288; doi:10.3390/jof9080841)
Supplement: Supplementary file 1 [file jof-09-00841-s001.zip › File S4_PDFs/File S4_(p-distances_ITS region).pdf]

This analysis involved 46 nucleotide sequences of the ITS region.
There were a total of 441 positions in the final dataset.

p-distance thresholds:
same species: 0−**3**%
same genus: ≤12%
between genera: >12%. Exceptions: **8−12%**
to the outgroups: 21−27%; 33−41%
between the species of the new genera and *C. maritima* : ≥14%

		2	3	4	5	6	7	8	9	10	11	12	13	14	15	16	17	18	19	20	21	22	23	24	25	26	27	28	29	30	31	32	33	34	35	36	37	38	39	40	41	42	43	44	45	46	47	
2	*Corollospora maritima* FCUL CaC1																																															
3	*Corollospora maritima* FCUL280207CF8	0.01																																														
4	*Corollospora maritima* CUB10 (KX261252)	0.01	0.01																																													
5	*Corollospora maritima* DB10 (KX261256)	0.01	0.01	0.00																																												
6	*Corollospora maritima* NBRC 32117 (JN943388)	0.01	0.01	0.00	0.00																																											
7	*Corollospora* sp. CMG 52 (MT235714)	0.05	0.04	0.04	0.04	0.04																																										
8	*Corollospora mediterranea* MUT 5040 (MW582549)	0.06	0.06	0.05	0.06	0.05	0.09																																									
9	*Corollospora mediterranea* MUT 5048 (MW582550)	0.06	0.06	0.05	0.06	0.05	0.09	0.00																																								
10	*Corollosporopsis portsaidica* MUT 1941 (KU935658)	0.18	0.18	0.18	0.18	0.18	0.22	0.20	0.20																																							
11	*Corollosporopsis portsaidica* IMI 395684 (NR175013)	0.18	0.18	0.17	0.17	0.17	0.21	0.19	0.19	0.02																																						
12	*Corollosporopsis* sp. 1 ACA 10 (KX261227)	0.18	0.17	0.17	0.17	0.17	0.21	0.19	0.19	0.03	0.03																																					
13	*Corollosporopsis* sp. 1 PV6 (KX261315)	0.18	0.17	0.17	0.17	0.17	0.21	0.19	0.19	0.03	0.03	0.00																																				
14	*Corollosporopsis* sp. 2 CAN5 (KX261239)	0.17	0.17	0.16	0.16	0.16	0.20	0.18	0.18	0.08	0.08	0.08	0.08																																			
15	*Corollosporopsis* sp. 2 CDC8 (KX261250)	0.17	0.17	0.17	0.17	0.16	0.21	0.18	0.18	0.08	0.08	0.08	0.08	0.00																																		
16	*Corollosporopsis* sp. 3 NBRC 32118 (JN943387)	0.18	0.18	0.18	0.18	0.18	0.22	0.19	0.19	0.04	0.04	0.04	0.04	0.08	0.09																																	
17	*Corollosporopsis* sp. 3 DUN7 (KX261267)	0.18	0.18	0.18	0.18	0.18	0.22	0.19	0.19	0.04	0.04	0.04	0.04	0.08	0.09	0.00																																
18	*Corollosporopsis* sp. 4 CUB9 (KX261255)	0.18	0.18	0.17	0.18	0.17	0.21	0.19	0.19	0.05	0.05	0.04	0.04	0.08	0.09	0.03	0.03																															
19	*Corollosporopsis* sp. 5 NBRC 106651	0.16	0.16	0.16	0.16	0.16	0.20	0.18	0.18	0.11	0.11	0.10	0.10	0.09	0.08	0.11	0.11	0.11																														
20	pNGenus A sp.1 HAW1 (KX261278)	0.16	0.15	0.14	0.15	0.15	0.19	0.17	0.17	0.15	0.14	0.14	0.14	0.14	0.14	0.15	0.15	0.15	0.13																													*Corollospora maritima.*
21	pNGenus A sp. 1 HAW2 (KX261279)	0.16	0.16	0.15	0.16	0.16	0.20	0.18	0.18	0.15	0.14	0.14	0.14	0.14	0.14	0.15	0.15	0.15	0.13	0.00																												*Corollospora maritima.*
22	pNGenus A sp. 2 CUB4 (KX261253)	0.15	0.15	0.14	0.15	0.15	0.19	0.17	0.17	0.17	0.16	0.15	0.15	0.15	0.15	0.17	0.17	0.16	0.14	0.04	0.04																											*Corollospora maritima.*
23	pNGenus A sp. 2 CUB7 (KX261254)	0.15	0.15	0.14	0.15	0.15	0.19	0.17	0.17	0.17	0.16	0.15	0.15	0.15	0.15	0.17	0.17	0.16	0.14	0.04	0.04	0.00																										*Corollospora maritima.*
24	*Corollosporella ramulos* a CBS:398.65 (MH858634)	0.17	0.17	0.16	0.16	0.16	0.20	0.17	0.17	0.19	0.18	0.17	0.17	0.18	0.18	0.19	0.19	0.18	0.16	0.17	0.17	0.17	0.17																									
25	*Corollosporella anglusa* NBRC 104919	0.17	0.17	0.17	0.17	0.17	0.20	0.18	0.18	0.17	0.17	0.16	0.16	0.17	0.17	0.19	0.19	0.18	0.15	0.16	0.16	0.17	0.17	0.05																								
26	*Shirahamella gracilis* 62 P1815996 001 (MT569588)	0.15	0.15	0.14	0.15	0.14	0.18	0.15	0.15	0.15	0.15	0.14	0.14	0.14	0.14	0.16	0.16	0.16	0.15	0.15	0.16	0.16	0.16	**0.12**	**0.12**																							
27	*Shirahamella gracilis* 14 A09 (MT569589)	0.15	0.15	0.14	0.15	0.14	0.18	0.15	0.15	0.15	0.15	0.14	0.14	0.14	0.14	0.16	0.16	0.16	0.15	0.15	0.16	0.16	0.16	**0.12**	**0.12**	0.00																						
28	*Shirahamella gracilis* NBRC 32111 (JN943386)	0.16	0.16	0.15	0.15	0.15	0.19	0.16	0.16	0.16	0.16	0.14	0.14	0.15	0.14	0.16	0.16	0.16	0.15	0.15	0.15	0.16	0.16	**0.12**	**0.12**	**0.03**	**0.03**																					
29	*Shirahamella* sp. 1 MD 828 (AB361026)	0.16	0.16	0.16	0.16	0.15	0.19	0.16	0.16	0.17	0.17	0.16	0.16	0.17	0.17	0.18	0.18	0.17	0.16	0.16	0.16	0.17	0.17	**0.12**	**0.12**	0.05	0.05	0.04																				
30	*Ajigaurospora pseudopulchella* NBRC 32112 (UDBO34332)	0.16	0.15	0.15	0.15	0.15	0.19	0.16	0.16	0.17	0.17	0.16	0.16	0.17	0.17	0.18	0.18	0.17	0.15	0.16	0.16	0.17	0.17	**0.09**	**0.09**	**0.10**	**0.10**	**0.10**	**0.11**																			
31	*Garethelia parvula* NBRC 32159 (FJ591159)	0.16	0.15	0.14	0.15	0.15	0.19	0.16	0.16	0.18	0.17	0.17	0.17	0.16	0.16	0.18	0.18	0.17	0.16	0.15	0.15	0.16	0.16	**0.11**	**0.10**	**0.12**	**0.12**	**0.12**	0.13	**0.08**																		
32	*Garethelia parvula* TUB6982 (EU557357)	0.16	0.16	0.14	0.15	0.15	0.19	0.16	0.16	0.18	0.17	0.17	0.17	0.16	0.16	0.18	0.18	0.17	0.16	0.16	0.16	0.16	0.16	**0.11**	**0.10**	**0.12**	**0.12**	**0.12**	0.13	**0.08**	0.00																	
33	*Paracorollospora angusta* NBRC 32102 (JN943383)	0.19	0.18	0.18	0.18	0.18	0.22	0.20	0.20	0.20	0.20	0.21	0.21	0.19	0.19	0.21	0.21	0.20	0.21	0.19	0.20	0.21	0.21	0.19	0.17	0.17	0.17	0.18	0.19	0.18	0.16	0.17																
34	*Paracorollospora angusta* NBRC 32101 (JN943381)	0.19	0.18	0.18	0.18	0.18	0.22	0.20	0.20	0.20	0.20	0.21	0.21	0.19	0.19	0.21	0.21	0.20	0.21	0.19	0.20	0.21	0.21	0.19	0.17	0.17	0.17	0.18	0.19	0.18	0.16	0.17	0.00															
35	*Paracorollospora marina* NBRC 103271 (EU557359)	0.19	0.18	0.19	0.19	0.19	0.22	0.19	0.19	0.20	0.19	0.20	0.20	0.19	0.19	0.20	0.20	0.20	0.18	0.18	0.18	0.18	0.18	0.18	0.17	0.17	0.17	0.18	0.19	0.18	0.17	0.17	0.11	0.11														
36	*Honshuriella fusca* NBRC 32107 (JN943382)	0.22	0.22	0.22	0.22	0.22	0.25	0.23	0.23	0.20	0.20	0.19	0.19	0.19	0.19	0.21	0.21	0.20	0.17	0.19	0.19	0.20	0.20	0.22	0.21	0.18	0.18	0.18	0.19	0.19	0.20	0.20	0.21	0.21	0.21													
37	*Honshuriella fusca* NBRC 32108 (JN943385)	0.22	0.22	0.22	0.22	0.22	0.25	0.23	0.23	0.20	0.20	0.19	0.19	0.19	0.19	0.21	0.21	0.20	0.17	0.19	0.19	0.20	0.20	0.22	0.21	0.18	0.18	0.18	0.19	0.19	0.20	0.20	0.21	0.21	0.21	0.00												
38	*Honshuriella* sp.1 NBRC 32109 (JN943384)	0.24	0.23	0.23	0.23	0.23	0.26	0.24	0.24	0.21	0.22	0.20	0.20	0.20	0.20	0.22	0.22	0.21	0.17	0.19	0.18	0.20	0.20	0.22	0.21	0.17	0.17	0.17	0.20	0.19	0.21	0.21	0.21	0.21	0.20	0.07	0.07											
39	*Keraliethelia pulchella* NBRC 31323	0.25	0.24	0.24	0.24	0.24	0.27	0.26	0.26	0.24	0.23	0.23	0.23	0.23	0.23	0.24	0.24	0.24	0.23	0.21	0.21	0.23	0.23	0.21	0.20	0.22	0.22	0.21	0.22	0.22	0.22	0.23	0.23	0.23	0.23	0.24	0.24	0.25										
40	*Keraliethelia pulchella* NBRC 32123 (JN943446)	0.26	0.25	0.25	0.25	0.25	0.28	0.27	0.27	0.24	0.24	0.25	0.25	0.23	0.23	0.25	0.25	0.24	0.22	0.21	0.21	0.22	0.22	0.21	0.21	0.22	0.22	0.22	0.22	0.23	0.22	0.22	0.24	0.24	0.24	0.23	0.23	0.26	**0.03**									
41	pNGenus B sp. 1 NBRC 32105 (JN943445)	0.24	0.24	0.23	0.24	0.23	0.27	0.24	0.24	0.18	0.19	0.18	0.18	0.18	0.18	0.19	0.19	0.18	0.19	0.23	0.23	0.24	0.24	0.22	0.22	0.21	0.21	0.21	0.22	0.22	0.23	0.23	0.22	0.22	0.21	0.24	0.24	0.24	0.25	0.24								*Corollospora colossa*
42	pNGenus C sp. 1 NBRC 106650	0.25	0.25	0.24	0.25	0.24	0.27	0.25	0.25	0.19	0.19	0.19	0.19	0.20	0.19	0.19	0.19	0.19	0.20	0.21	0.21	0.22	0.22	0.25	0.24	0.22	0.22	0.22	0.23	0.25	0.22	0.22	0.23	0.23	0.22	0.23	0.23	0.23	0.26	0.25	0.15							*Corollospora colossa*
43	*Nakagariella filiformis* CBS:125146 (MH863458)	0.20	0.20	0.20	0.19	0.20	0.23	0.20	0.20	0.21	0.21	0.21	0.21	0.21	0.21	0.21	0.21	0.21	0.17	0.18	0.19	0.18	0.18	0.19	0.18	0.16	0.16	0.18	0.19	0.19	0.19	0.20	0.23	0.23	0.20	0.22	0.22	0.21	0.20	0.19	0.21	0.22						
44	*Cucullosporella mangrovei* NBRC 32477	0.24	0.24	0.24	0.23	0.24	0.27	0.25	0.25	0.25	0.24	0.24	0.24	0.25	0.25	0.25	0.25	0.25	0.24	0.21	0.21	0.24	0.24	0.25	0.24	0.24	0.24	0.24	0.25	0.24	0.25	0.25	0.25	0.25	0.24	0.26	0.26	0.27	0.25	0.27	0.27	0.26	0.26					
45	*Cucullosporella mangrovei* NBRC 105272	0.24	0.24	0.24	0.24	0.24	0.27	0.25	0.25	0.25	0.25	0.24	0.24	0.26	0.25	0.25	0.25	0.25	0.24	0.21	0.21	0.24	0.24	0.25	0.24	0.24	0.24	0.24	0.25	0.25	0.25	0.25	0.25	0.25	0.24	0.26	0.26	0.27	0.26	0.27	0.27	0.26	0.26	0.00				
46	*Trailia ascophylli* NBRC 106638	0.36	0.36	0.36	0.36	0.36	0.38	0.36	0.36	0.36	0.36	0.36	0.36	0.36	0.36	0.36	0.36	0.36	0.36	0.37	0.37	0.36	0.36	0.35	0.35	0.36	0.36	0.35	0.36	0.35	0.35	0.35	0.33	0.33	0.35	0.37	0.37	0.36	0.41	0.40	0.37	0.35	0.38	0.37	0.37			
47	*Trailia ascophylli* NBRC 106637	0.36	0.36	0.36	0.36	0.36	0.38	0.36	0.36	0.36	0.36	0.36	0.36	0.36	0.36	0.36	0.36	0.36	0.36	0.37	0.37	0.36	0.36	0.35	0.35	0.36	0.36	0.35	0.36	0.35	0.35	0.35	0.33	0.33	0.35	0.37	0.37	0.36	0.41	0.40	0.37	0.35	0.38	0.37	0.37	0.00		

Current identification
