# Supplementary material for: Redefining the Genus Corollospora Based on Morphological and Phylogenetic Approaches
Source: J Fungi (Basel). 2023 Aug 11;9(8):841. doi: 10.3390/jof9080841 (PMC10455288; doi:10.3390/jof9080841)
Supplement: Supplementary file 1 [file jof-09-00841-s001.zip › Table S1.pdf]

Table S1			Sequences retrieved from the databases					
Taxon (according to the present study)	Source	Registered Taxon	Access Number (Remarks)			Sequence length (Nº nucleotides)		
			18S gene	ITS region	28S gene	18S gene	ITS	28S gene
Keraliethelia pulchella	NBRC 8152	*Clavariopsis bulbosa*	—	—	NBRC 8152	—	—	569
Keraliethelia pulchella	*NBRC 31323	*Clavariopsis bulbosa*	—	NBRC 31323	NBRC 31323	—	677	569
Keraliethelia pulchella	NBRC 31324	*Clavariopsis bulbosa*	—	—	NBRC 31324	—	—	580
Keraliethelia pulchella	NBRC 8152	*Corollospora pulchella*			AB361021	—	—	1230
Keraliethelia pulchella	NBRC 32124	*Corollospora pulchella*	JN941649 (FBC) (d)	—	JN941497 (FBC)	1094	—	897
Keraliethelia pulchella	NBRC 32123	*Corollospora pulchella*	JN941650 (FBC)	JN943446 (FBC)	JN941496 (FBC)	1091	1289	897
Keraliethelia pulchella	NTOU5721	*Corollospora pulchella*	—	—	OQ130072	—	—	990
Keraliethelia pulchella	NTOU5725	*Corollospora pulchella*	—	—	OQ130070	—	—	991
Keraliethelia pulchella	NTOU5727	*Corollospora pulchella*	—	—	OQ130071	—	—	992
Keraliethelia sp. 1	NTOU5730	*Corollospora pulchella*	—	—	OQ130076	—	—	993
Keraliethelia sp. 1	NTOU5732	*Corollospora pulchella*	—	—	OQ130075	—	—	994
Keraliethelia sp. 1	NTOU5738	*Corollospora pulchella*	—	—	OQ130074	—	—	995
Keraliethelia sp. 1	NTOU5741	*Corollospora pulchella*	—	—	OQ130073	—	—	992
Inconclusive placement (see notes)	PP4294	*Corollospora pulchella*	—	—	AF491261 (FBC) (d)	—	—	603
Inconclusive placement (see notes)	PP4206	*Corollospora pulchella*	—	—	AF491274 (d)	—	—	721
Alternaria sp. **EXCLUDED**	MUT 399	*Corollospora anglusa*	—	MK578241	—	—	438	—
Corollosporella anglusa	MF 827	*Corollospora anglusa*	—	AB361022 (c, d)	AB361008	—	598	1331
Corollosporella anglusa	* NBRC 104919 (MF 827)	*Corollospora anglusa*	—	NBRC 104919	NBRC 104919	—	637	565
Paracorollospora angusta	KUC21260	*Corollospora angusta*	—	KT207778	KT207728	—	612	1080
Paracorollospora angusta	KUC21258	*Corollospora angusta*	—	KT207776	KT207726	—	614	789
Paracorollospora angusta	KUC21257	*Corollospora angusta*	—	KT207775	KT207725	—	612	989
Paracorollospora angusta	KUC21256	*Corollospora angusta*	—	KT207774	KT207724	—	612	989
Paracorollospora angusta	KUC21255	*Corollospora angusta*	—	KT207773	KT207723	—	612	864
Paracorollospora angusta	KUC21254	*Corollospora angusta*	—	KT207772	KT207722	—	596	1080
Paracorollospora angusta	KUC21252	*Corollospora angusta*	—	KT207770	KT207720	—	612	1080
Paracorollospora angusta	KUC21251	*Corollospora angusta*	—	KT207769	KT207719	—	612	1080
Paracorollospora angusta	KUC21250	*Corollospora angusta*	—	KT207768	KT207718	—	600	779
Paracorollospora angusta	KUC21249	*Corollospora angusta*	—	KT207767	KT207717	—	612	781

Paracorollospora angusta	KUC21248	*Corollospora angusta*	—	KT207766	KT207716	—	612	1080
Paracorollospora angusta	KUC21247	*Corollospora angusta*	—	KT207765	KT207715	—	612	857
Paracorollospora angusta	KUC21246	*Corollospora angusta*	—	KT207764	KT207714	—	612	—
Paracorollospora angusta	NBRC 32102	*Corollospora angusta*	NBRC 32102	JN943383 (FBC)	JN941478 (FBC)	1751	616	888
Paracorollospora angusta	*NBRC 32101	*Corollospora angusta*	NBRC 32101	JN943381 (FBC)	JN941477 (FBC)	1751	1180	888
Paracorollospora angusta	NBRC 32100	*Corollospora angusta*	NBRC 32100 (FBC)	JN943380 (FBC)	JN941476 (FBC)	1751	616	888
Paracorollospora angusta	TUB1	*Corollospora angusta*	—	EU557360 (AFToL)	—	—	683	—
			—	—	AY305031	—	—	526
Paracorollospora angusta	PP3903	*Corollospora angusta*	—	—	AF491254	—	—	559
Not subjected to the global evaluation (see notes)	AFTOL-ID 5010	*Corollospora angusta*	FJ176845 (AFToL) (d)	—	FJ176900 (AFToL)	572	—	1211
pNGenus D; sp. 1 (see notes)	NBRC 32125	*Corollospora cinnamomea*	—	AB361023 (b,c)	—	—	471	—
pNGenus D; sp. 1 (see notes)			JN941665 (FBC)	—	JN941479 (FBC)	1094	—	893
pNGenus D; sp. 1 (see notes)	NBRC 32126	*Corollospora cinnamomea*	JN941666 (FBC)	—	JN941480 (FBC)	1094	—	893
pNGenus D; sp. 1 (see notes)	NTOU4949	*Corollospora cinnamomea*	—	—	OQ130064	—	—	986
pNGenus D; sp. 1 (see notes)	NTOU4950	*Corollospora cinnamomea*	—	—	OQ130065	—	—	986
Tokurathelia colossa	*NBRC 32103	*Corollospora colossa*	JN941664 (FBC) (d)	—	JN941481 (FBC)	1094	—	933
Not subjected to the global evaluation (see notes)	PP3904	*Corollospora colossa*	—	EU557366 (AFToL) (a)	—	—	167	—
			—	—	AF491255 (d)	—	—	623
pNGenus B; sp. 1	NBRC 32105	*Corollospora colossa*	JN941663 (FBC)	JN943445 (FBC)	JN941482 (FBC)	1205	1083	904
pNGenus C; sp. 1	NBRC 106650	*Corollospora colossa*	—	NBRC 106650	NBRC 106650	—	526	572
Nakagariella filiformis	*NBRC 32106	*Corollospora filiformis*	—	—	NBRC 32106	—	—	571
Nakagariella filiformis (see notes)	*CBS 125146	*Corollospora filiformis*	—	MH863458	MH874958	—	665	910
Nakagariella filiformis	PP3905	*Corollospora filiformis*	—	—	AF491256	—	—	539
Nakagariella filiformis	NTOU5709	*Corollospora filiformis*	—	—	OQ130068	—	—	993
Nakagariella filiformis	NTOU5712	*Corollospora filiformis*	—	—	OQ130066	—	—	993
Nakagariella filiformis	NTOU5714	*Corollospora filiformis*	—	—	OQ130067	—	—	993
Not subjected to the global evaluation (see notes)	PP3906	*Corollospora fusca*	—	—	AF491257 (d)	—	—	602
Honshuriella fusca	NBRC 32108	*Corollospora fusca*	JN941661 (FBC) (d)	JN943385 (FBC)	JN941484 (FBC)	1094	1203	964
Honshuriella fusca	*NBRC 32107	*Corollospora fusca*	JN941662 (FBC)	JN943382 (FBC)	JN941483 (FBC)	1548	605	964
Honshuriella sp.	NBRC 32109	*Corollospora fusca*	—	JN943384 (FBC)	JN941485 (FBC)	—	602	897
Shirahamella gracilis	23	*Corollospora gracilis*	—	MT569575	—	—	546	—

Shirahamella gracilis	9 E08	*Corollospora gracilis*	—	MT569576	—	—	546	—
Shirahamella gracilis	62 P1815996 001	*Corollospora gracilis*	—	MT569588	—	—	627	—
Shirahamella gracilis	14 A09	*Corollospora gracilis*	—	MT569589	—	—	627	—
Shirahamella gracilis	5 D08	*Corollospora gracilis*	—	MT569590	—	—	627	—
Shirahamella gracilis	30 E10	*Corollospora gracilis*	—	MT569591	—	—	627	—
Shirahamella gracilis	M3-1G F02	*Corollospora gracilis*	—	MT569592	—	—	627	—
Shirahamella gracilis	11 G08	*Corollospora gracilis*	—	MT569593	—	—	627	—
Shirahamella gracilis	112 P1815996 096	*Corollospora gracilis*	—	MT569594	—	—	627	—
Shirahamella gracilis	111 P1815996 095	*Corollospora gracilis*	—	MT569595	—	—	627	—
Shirahamella gracilis	1 A08	*Corollospora gracilis*	—	MT569596	—	—	627	—
Shirahamella gracilis	4 C08	*Corollospora gracilis*	—	MT569597	—	—	627	—
Shirahamella gracilis	26 A10	*Corollospora gracilis*	—	MT569598	—	—	627	—
Shirahamella gracilis	10 F08	*Corollospora gracilis*	—	MT569599	—	—	627	—
Shirahamella gracilis	28 C10	*Corollospora gracilis*	—	MT569600	—	—	627	—
Shirahamella gracilis	SFC102400	*Corollospora gracilis*	—	MH374560	—	—	627	—
Shirahamella gracilis	NBRC 32111	*Corollospora gracilis*	JN941659 (FBC)	JN943386 (FBC)	JN941487 (FBC)	1750	625	892
Shirahamella gracilis	*NBRC 32110	*Corollospora gracilis*	JN941660 (FBC)	AB361025 (a)	JN941486 (FBC)	1750	206	892
Shirahamella sp. 1	MD 828	*Corollospora gracilis*	—	AB361026	AB361019	—	730	870
Inconclusive placement (see Table 4)	PP3910	*Corollospora intermedia*	—	—	AF491258 (b, d)	—	—	672
Not subjected to the global evaluation (see notes)	—	*Corollospora intermedia*	—	EU557363 (AFToL) (a)	—	—	375	—
Controversial placement (*Corollospora intermedia* ?) (see	NBRC 104402	*Corollospora intermedia*	NBRC 104402	NBRC 104402	NBRC 104402	1751	652	565
pNGenus E; sp. 1 (see notes)	NBRC 32121	*Corollospora lacera*	JN941658 (FBC)	—	JN941488 (FBC)	1751	—	889
pNGenus E; sp. 1 (see notes)	NBRC 32122	*Corollospora lacera*	JN941657 (FBC)	—	JN941489 (FBC)	1751	—	889
Inconclusive placement (see notes)	PP2509	*Corollospora lacera*	—	—	AF491259	—	—	742
Not subjected to the global evaluation (see notes)	LF813	*Corollospora lacera*	KM096212	—	—	1443	—	—
Paracorollospora luteola	*NBRC 31315 (TKB-C-1250)	*Corollospora luteola*	JN941656 (FBC)	—	JN941490 (FBC)	1751	—	891
Paracorollospora luteola	NBRC 31316	*Corollospora luteola*	JN941491 (FBC)	—	JN941491 (FBC)	1751	—	891
Paracorollospora luteola	—	*Corollospora luteola*	DQ104805	—	DQ104809	1058	—	1210
Inconclusive placement (see notes)	MD 825	*Corollospora maritima*	—	AB361032 (b)	AB361012	—	450	1222
Corollospora maritima	SON9	*Corollospora maritima*	—	KX261321	—	—	518	—
Corollospora maritima	SON7	*Corollospora maritima*	—	KX261320	—	—	518	—

Corollospora maritima	SON6	*Corollospora maritima*	—	KX261319	—	—	519	—
Corollospora maritima	SON5	*Corollospora maritima*	—	KX261318	—	—	519	—
Corollospora maritima	SON3	*Corollospora maritima*	—	KX261317	—	—	519	—
Corollospora maritima	SON2	*Corollospora maritima*	—	KX261316	—	—	519	—
Corollospora maritima	PRO9	*Corollospora maritima*	—	KX261312	—	—	519	—
Corollospora maritima	PRO8	*Corollospora maritima*	—	KX261311	—	—	519	—
Corollospora maritima	PRO7bis	*Corollospora maritima*	—	KX261310	—	—	520	—
Corollospora maritima	PRO4	*Corollospora maritima*	—	KX261309	—	—	520	—
Corollospora maritima	PRO3	*Corollospora maritima*	—	KX261308	—	—	520	—
Corollospora maritima	MIR9	*Corollospora maritima*	—	KX261298	—	—	519	—
Corollospora maritima	MIR7	*Corollospora maritima*	—	KX261297	—	—	519	—
Corollospora maritima	MIR6	*Corollospora maritima*	—	KX261296	—	—	519	—
Corollospora maritima	MIR4	*Corollospora maritima*	—	KX261295	—	—	519	—
Corollospora maritima	MIR3	*Corollospora maritima*	—	KX261294	—	—	519	—
Corollospora maritima	MIR2	*Corollospora maritima*	—	KX261293	—	—	519	—
Corollospora maritima	MIR1	*Corollospora maritima*	—	KX261292	—	—	519	—
Corollospora maritima	LP8	*Corollospora maritima*	—	KX261290	—	—	519	—
Corollospora maritima	LP3	*Corollospora maritima*	—	KX261289	—	—	519	—
Corollospora maritima	LP2	*Corollospora maritima*	—	KX261288	—	—	519	—
Corollospora maritima	LP1	*Corollospora maritima*	—	KX261287	—	—	519	—
Corollospora maritima	DUN8	*Corollospora maritima*	—	KX261268	—	—	519	—
Corollospora maritima	DUN3	*Corollospora maritima*	—	KX261266	—	—	519	—
Corollospora maritima	DUN2	*Corollospora maritima*	—	KX261265	—	—	519	—
Corollospora maritima	DB9	*Corollospora maritima*	—	KX261264	—	—	520	—
Corollospora maritima	DB8	*Corollospora maritima*	—	KX261263	—	—	520	—
Corollospora maritima	DB7	*Corollospora maritima*	—	KX261262	—	—	520	—
Corollospora maritima	DB6	*Corollospora maritima*	—	KX261260	—	—	518	—
Corollospora maritima	DB4	*Corollospora maritima*	—	KX261259	—	—	519	—
Corollospora maritima	DB3	*Corollospora maritima*	—	KX261258	—	—	519	—
Corollospora maritima	DB1	*Corollospora maritima*	—	KX261257	—	—	519	—
Corollospora maritima	DB10	*Corollospora maritima*	—	KX261256	—	—	520	—

Corollospora maritima	CUB10	*Corollospora maritima*	—	KX261252	—	—	518	—
Corollospora maritima	CDC6	*Corollospora maritima*	—	KX261249	—	—	519	—
Corollospora maritima	CDC5	*Corollospora maritima*	—	KX261248	—	—	519	—
Corollospora maritima	CDC4	*Corollospora maritima*	—	KX261247	—	—	519	—
Corollospora maritima	CDC3	*Corollospora maritima*	—	KX261246	—	—	519	—
Corollospora maritima	CDC2	*Corollospora maritima*	—	KX261245	—	—	519	—
Corollospora maritima	CDC1	*Corollospora maritima*	—	KX261244	—	—	519	—
Corollospora maritima	CDC10	*Corollospora maritima*	—	KX261243	—	—	519	—
Corollospora maritima	NBRC 32117	*Corollospora maritima*	JN941654<br>(FBC)	JN943388<br>(FBC)	JN941492<br>(FBC)	1091	1231	893
Corollospora maritima	MD 835	*Corollospora maritima*	—	AB361027	AB361009	—	700	864
Corollospora maritima	MD 831	*Corollospora maritima*	—	AB361028	AB361010	—	653	1314
Corollospora maritima	MD 812	*Corollospora maritima*	—	AB361029	AB361011	—	600	865
Corollospora maritima	PAV-M 144	*Corollospora maritima*	—	KF993401	—	—	645	—
Corollospora maritima	MUT 5102	*Corollospora maritima*	—	MK581072	—	—	619	—
Corollospora maritima	SIC-023	*Corollospora maritima*	—	MW218880	—	—	481	—
Corollospora maritima	NBRC 106640	*Corollospora maritima*	—	NBRC 106640	NBRC 106640	—	632	565
Corollospora maritima	NTOU4008	*Corollospora maritima*	KX686769	—	KX686770	1018	—	989
Corollospora maritima	AFTOL-ID 5011	*Corollospora maritima*	FJ176846<br>(AFToL)	—	FJ176901<br>(AFToL)	991	—	865
Corollospora maritima	01092011III.3	*Corollospora maritima*	—	KM272367	KM272359	—	598	882
Corollospora maritima	14	*Corollospora maritima*	—	MT569527	—	—	626	—
Corollospora maritima	3 P1815	*Corollospora maritima*	—	MT569528	—	—	626	—
Corollospora maritima	20 D05	*Corollospora maritima*	—	MT569544	—	—	591	—
Corollospora maritima	29	*Corollospora maritima*	—	MT569545	—	—	578	—
Corollospora maritima	11	*Corollospora maritima*	—	MT569546	—	—	588	—
Corollospora maritima	2	*Corollospora maritima*	—	MT569547	—	—	588	—
Corollospora maritima	9	*Corollospora maritima*	—	MT569548	—	—	588	—
Corollospora maritima	10	*Corollospora maritima*	—	MT569549	—	—	588	—
Corollospora maritima	12	*Corollospora maritima*	—	MT569550	—	—	588	—
Corollospora maritima	20	*Corollospora maritima*	—	MT569551	—	—	588	—
Corollospora maritima	6	*Corollospora maritima*	—	MT569552	—	—	588	—
Corollospora maritima	M10 4	*Corollospora maritima*	—	MT569553	—	—	538	—

Corollospora maritima	102	*Corollospora maritima*	—	MT569560	—	—	628	—
Corollospora maritima	34	*Corollospora maritima*	—	MT569561	—	—	628	—
Corollospora maritima	21	*Corollospora maritima*	—	MT569562	—	—	628	—
Corollospora maritima	31	*Corollospora maritima*	—	MT569563	—	—	628	—
Corollospora maritima	47	*Corollospora maritima*	—	MT569564	—	—	628	—
Corollospora maritima	7	*Corollospora maritima*	—	MT569565	—	—	628	—
Corollospora maritima	15	*Corollospora maritima*	—	MT569566	—	—	628	—
Corollospora maritima	28	*Corollospora maritima*	—	MT569567	—	—	628	—
Corollospora maritima	17	*Corollospora maritima*	—	MT569568	—	—	628	—
Corollospora maritima	M10 2	*Corollospora maritima*	—	MT569569	—	—	628	—
Corollospora maritima	19	*Corollospora maritima*	—	MT569570	—	—	590	—
Corollospora maritima	27	*Corollospora maritima*	—	MT569571	—	—	590	—
Corollospora maritima	24	*Corollospora maritima*	—	MT569572	—	—	620	—
Corollospora maritima	18 P1815996 073	*Corollospora maritima*	—	MT569580	—	—	572	—
Corollospora maritima	1 P1815996 028	*Corollospora maritima*	—	MT569581	—	—	572	—
Corollospora maritima	32 P1815996 072	*Corollospora maritima*	—	MT569582	—	—	572	—
Corollospora maritima	16 P185996 057	*Corollospora maritima*	—	MT569583	—	—	572	—
Corollospora maritima	123 P1815996 093	*Corollospora maritima*	—	MT569584	—	—	555	—
Corollospora maritima	22 P1815996 007	*Corollospora maritima*	—	MT569585	—	—	555	—
Corollospora maritima	23 P1815996 008	*Corollospora maritima*	—	MT569586	—	—	587	—
Corollospora maritima	MUT 1652	*Corollospora maritima*	MW543046	MW543050	MW543054	1018	587	1260
Corollospora maritima	MUT 1662	*Corollospora maritima*	MW543047	MW543051	MW543055	1042	526	1285
Corollospora maritima	MUT 1663	*Corollospora maritima*	—	KF915979	—	—	567	—
Corollospora maritima	MUT 3370	*Corollospora maritima*	—	KF915985	—	—	550	—
Corollospora maritima	MUT 3408	*Corollospora maritima*	MW543048	MW543052	MW543056	1025	621	1292
Corollospora maritima	MUT 3410	*Corollospora maritima*	MW543049	MW543053	MW543057	1019	623	1234
Corollospora maritima	SFC102442	*Corollospora maritima*	—	MH374552	—	—	634	—
Corollospora maritima	PP1991	*Corollospora maritima*	—	—	AF491260	—	—	859
Corollospora maritima	AW2871	*Corollospora maritima*	—	MT802512	—	—	573	—
Corollospora sp. 1	<u>CMG 52</u>	*Corollospora maritima*	—	<u>MT235714</u>	<u>MT232383</u>	—	612	1229
Corollospora mediterranea	MUT 1938	*Corollospora mediterranea*	MW584966	MW582548	MW584957	1434	580	1429

Corollospora mediterranea	MUT 5040	*Corollospora mediterranea*	MW584967	MW582549	MW584958	1450	625	1421
Corollospora mediterranea	MUT 5048	*Corollospora mediterranea*	MW584968	MW582550	MW584959	1270	624	1351
Corollospora mediterranea	MUT 5049	*Corollospora mediterranea*	MW584969	MW582551	MW584960	1086	628	943
Corollospora mediterranea	MUT 5082	*Corollospora mediterranea*	—	MW582552	MW584961	—	550	1030
Corollospora mediterranea	MUT 1587	*Corollospora mediterranea*	MW584971	KF915998	MW584962	1321	628	1340
Corollospora mediterranea	*MUT 1950	*Corollospora mediterranea*	NG_077446	KU935664	MW584963	1073	487	1137
Corollospora mediterranea	MUT 1954	*Corollospora mediterranea*	MW584973	KU935659 (a)	MW584964	1391	418	1420
Corollospora mediterranea	MUT 1961	*Corollospora mediterranea*	MW584974	KU935662	MW584965	1393	475	840
pNGenus A; sp. 1	CUB7	*Corollospora maritima*	—	KX261254	—	—	448	—
pNGenus A; sp. 1	CUB4	*Corollospora maritima*	—	KX261253	—	—	448	—
pNGenus A; sp. 2	HAW9	*Corollospora maritima*	—	KX261286	—	—	489	—
pNGenus A; sp. 2	HAW8	*Corollospora maritima*	—	KX261285	—	—	489	—
pNGenus A; sp. 2	HAW7	*Corollospora maritima*	—	KX261284	—	—	489	—
pNGenus A; sp. 2	HAW6	*Corollospora maritima*	—	KX261283	—	—	489	—
pNGenus A; sp. 2	HAW5	*Corollospora maritima*	—	KX261282	—	—	489	—
pNGenus A; sp. 2	HAW4	*Corollospora maritima*	—	KX261281	—	—	489	—
pNGenus A; sp. 2	HAW3	*Corollospora maritima*	—	KX261280	—	—	489	—
pNGenus A; sp. 2	HAW2	*Corollospora maritima*	—	KX261279	—	—	489	—
pNGenus A; sp. 2	HAW1	*Corollospora maritima*	—	KX261278	—	—	489	—
Not subjected to the global evaluation	JK 4834	*Corollospora maritima*	U46871	—	U46884	1031	—	878
Corollosporopsis sp. 3	CAN9	*Corollospora maritima*	—	KX261242	—	—	500	—
Corollosporopsis sp. 3	CAN8	*Corollospora maritima*	—	KX261241	—	—	500	—
Corollosporopsis sp. 3	CAN6	*Corollospora maritima*	—	KX261240	—	—	500	—
Corollosporopsis sp. 3	CAN5	*Corollospora maritima*	—	KX261239	—	—	500	—
Corollosporopsis sp. 3	CDC8	*Corollospora maritima*	—	KX261250	—	—	496	—
Corollosporopsis sp. 5	NBRC 106651 (*)	*Corollospora maritima*	—	NBRC 106651	NBRC 106651	—	590	563
Corollosporopsis sp. 2	ACA10	*Corollospora maritima*	—	KX261227	—	—	506	—
Corollosporopsis sp. 2	BR1	*Corollospora maritima*	—	KX261229	—	—	506	—
Corollosporopsis sp. 2	BR2	*Corollospora maritima*	—	KX261230	—	—	505	—
Corollosporopsis sp. 2	BR3	*Corollospora maritima*	—	KX261231	—	—	505	—
Corollosporopsis sp. 2	BR4	*Corollospora maritima*	—	KX261232	—	—	505	—

Corollosporopsis sp. 2	BR5	*Corollospora maritima*	—	KX261233	—	—	506	—
Corollosporopsis sp. 2	BR6	*Corollospora maritima*	—	KX261234	—	—	507	—
Corollosporopsis sp. 2	BR7	*Corollospora maritima*	—	KX261235	—	—	507	—
Corollosporopsis sp. 2	BR8	*Corollospora maritima*	—	KX261236	—	—	505	—
Corollosporopsis sp. 2	BR9	*Corollospora maritima*	—	KX261237	—	—	505	—
Corollosporopsis sp. 2	BR10	*Corollospora maritima*	—	KX261228	—	—	505	—
Corollosporopsis sp. 2	TUX7	*Corollospora maritima*	—	KX261334	—	—	505	—
Corollosporopsis sp. 2	TUX2	*Corollospora maritima*	—	KX261331	—	—	505	—
Corollosporopsis sp. 2	TUX1	*Corollospora maritima*	—	KX261330	—	—	495	—
Corollosporopsis sp. 2	TUX10	*Corollospora maritima*	—	KX261329	—	—	505	—
Corollosporopsis sp. 2	TUX9	*Corollospora maritima*	—	KX261336	—	—	505	—
Corollosporopsis sp. 2	TUX8	*Corollospora maritima*	—	KX261335	—	—	505	—
Corollosporopsis sp. 2	TUX6	*Corollospora maritima*	—	KX261333	—	—	505	—
Corollosporopsis sp. 2	TUX4	*Corollospora maritima*	—	KX261332	—	—	505	—
Corollosporopsis sp. 2	TAB8	*Corollospora maritima*	—	KX261328	—	—	505	—
Corollosporopsis sp. 2	TAB7	*Corollospora maritima*	—	KX261327	—	—	505	—
Corollosporopsis sp. 2	TAB6	*Corollospora maritima*	—	KX261326	—	—	495	—
Corollosporopsis sp. 2	TAB5	*Corollospora maritima*	—	KX261325	—	—	505	—
Corollosporopsis sp. 2	TAB4	*Corollospora maritima*	—	KX261324	—	—	505	—
Corollosporopsis sp. 2	TAB2	*Corollospora maritima*	—	KX261323	—	—	505	—
Corollosporopsis sp. 2	TAB10	*Corollospora maritima*	—	KX261322	—	—	506	—
Corollosporopsis sp. 2	PV6	*Corollospora maritima*	—	KX261315	—	—	506	—
Corollosporopsis sp. 2	PV4	*Corollospora maritima*	—	KX261314	—	—	506	—
Corollosporopsis sp. 2	PV3	*Corollospora maritima*	—	KX261313	—	—	503	—
Corollosporopsis sp. 2	PAN3	*Corollospora maritima*	—	KX261307	—	—	503	—
Corollosporopsis sp. 2	PAN2	*Corollospora maritima*	—	KX261306	—	—	505	—
Corollosporopsis sp. 2	NAU8	*Corollospora maritima*	—	KX261305	—	—	505	—
Corollosporopsis sp. 2	NAU7	*Corollospora maritima*	—	KX261304	—	—	505	—
Corollosporopsis sp. 2	NAU5	*Corollospora maritima*	—	KX261303	—	—	505	—
Corollosporopsis sp. 2	NAU4	*Corollospora maritima*	—	KX261302	—	—	503	—
Corollosporopsis sp. 2	NAU3	*Corollospora maritima*	—	KX261301	—	—	505	—

Corollosporopsis sp. 2	NAU2	*Corollospora maritima*	—	KX261300	—	—	506	—
Corollosporopsis sp. 2	NAU10	*Corollospora maritima*	—	KX261299	—	—	507	—
Corollosporopsis sp. 2	MIR10	*Corollospora maritima*	—	KX261291	—	—	506	—
Corollosporopsis sp. 2	ESC9	*Corollospora maritima*	—	KX261277	—	—	506	—
Corollosporopsis sp. 2	ESC8	*Corollospora maritima*	—	KX261276	—	—	505	—
Corollosporopsis sp. 2	ESC7	*Corollospora maritima*	—	KX261275	—	—	506	—
Corollosporopsis sp. 2	ESC6	*Corollospora maritima*	—	KX261274	—	—	505	—
Corollosporopsis sp. 2	ESC3	*Corollospora maritima*	—	KX261273	—	—	478	—
Corollosporopsis sp. 2	ESC2	*Corollospora maritima*	—	KX261272	—	—	505	—
Corollosporopsis sp. 2	EC4	*Corollospora maritima*	—	KX261271	—	—	503	—
Corollosporopsis sp. 2	EC3	*Corollospora maritima*	—	KX261270	—	—	503	—
Corollosporopsis sp. 2	EC1	*Corollospora maritima*	—	KX261269	—	—	503	—
Corollosporopsis sp. 2	CDC9	*Corollospora maritima*	—	KX261251	—	—	505	—
Corollosporopsis sp. 2	CAN10	*Corollospora maritima*	—	KX261238	—	—	492	—
Corollosporopsis sp. 1	NBRC 32118 (*)	*Corollospora maritima*	JN941653 (FBC)	JN943387 (FBC)	JN941493 (FBC)	1094	611	956
Corollosporopsis sp. 1	DUN7	*Corollospora maritima*	—	KX261267	—	—	500	—
Corollosporopsis sp. 4	CUB9	*Corollospora maritima*	—	KX261255	—	—	492	—
Corollosporopsis portsaidica	MD 1301	*Corollospora portsaidica*	—		KJ406564	—	—	1377
Corollosporopsis portsaidica	MUT 1941	*Corollospora portsaidica*	—	KU935658	—	—	520	—
			—	NR_175013	NG_078629	—	699	1369
Corollosporopsis portsaidica	*MF 832 (IMI 395684 / MD 832)	*Corollospora portsaidica*		AB361031	AB361016			1369
			—	NBRC 105265	NBRC 105265	—	699	1369
Ajigaurospora pseudopulchella	NBRC 32113	*Corollospora pseudopulchella*	JN941651 (FBC)	—	JN941495 (FBC)	1094	—	947
Ajigaurospora pseudopulchella	*NBRC 32112	*Corollospora pseudopulchella*	JN941652 (FBC)	UDBO34332 (UNITE)	JN941494 (FBC)	1094	—	947
pNGenus F; sp. 1	NBRC 106641	*Corollospora pseudopulchella*	—	—	NBRC 106641	—	—	562
pNGenus F; sp. 1	NBRC 106642	*Corollospora pseudopulchella*	—	—	NBRC 106642	—	—	562
Corollospora quinqueseptata	*NBRC 32115	*Corollospora quinqueseptata*	JN941647 (FBC)	—	JN941499 (FBC)	1751	—	891
Corollospora quinqueseptata	NBRC 32114	*Corollospora quinqueseptata*	JN941648 (FBC)	—	JN941498 (FBC)	1108	—	891
pNGenus G; sp. 1	PP3909	*Corollospora quinqueseptata*	—	—	AF491262	—	—	664
Not subjected to the global evaluation (see notes)	LF747	*Corollospora quinqueseptata*	KM096200	—	—	1488	—	—
Not subjected to the global evaluation (see notes)	TUB557	*Sigmoidea luteola*	—	EU557358 (FBC) (a)	—	—	381	—

Paracorollospora marina	TUB2	*Sigmoidea marina*	—	—	AY305032	—	—	530
Paracorollospora marina	*NBRC 103271	*Sigmoidea marina*	—	EU557359 (AFToL)	—	—	635	—
Paracorollospora marina			—	—	NBRC 103271	—	—	562
Paracorollospora marina	AFTOL-ID 5008	*Sigmoidea marina*	FJ176843 (AFToL)	—	FJ176898 (AFToL)	1261	—	784
Paracorollospora marina	—	*Sigmoidea marina*	—	—	DQ104811	—	—	1208
Garethelia parvula	NBRC 666	*Sigmoidea parvula*	—	FJ591161	—	—	658	—
Garethelia parvula	NBRC 32160	*Sigmoidea parvula*	—	FJ591160	NBRC 32160	—	658	1219
Garethelia parvula	NBRC 32159 / IFO32159	*Sigmoidea parvula*	—	FJ591159 (e)	—	—	658	—
Garethelia parvula			DQ104806 (e)	—	DQ104810 (e)	1216	—	563
Garethelia parvula	TUB6982	*Sigmoidea parvula*	—	EU557357 (AFToL)	—	—	518	—
Garethelia parvula	TUB6989	*Sigmoidea parvula*	—	EU557356 (AFToL)	—	—	415	—
Garethelia parvula	*CBS 116644	*Sigmoidea parvula*	—	MH863000	MH874556	—	674	899
Garethelia parvula	NBRC 103558	*Sigmoidea parvula*	—	—	NBRC 103558	—	—	563
Garethelia parvula	NBRC 106655	*Sigmoidea parvula*	—	—	NBRC 106655	—	—	563
Garethelia parvula	NBRC 106659	*Sigmoidea parvula*	—	—	NBRC 106659	—	—	563
Garethelia parvula	NBRC 106660	*Sigmoidea parvula*	—	—	NBRC 106660	—	—	563
Controversial placement (*Corollosporella prolifera* ?) (see	NBRC 32120	*Varicosporina proflifera*	NBRC 32120	NBRC 32120	NBRC 32120	1751	672	565
Corollosporella ramulosa	*CBS 398.65	*Varicosporina ramulosa*	—	MH858634	MH870274	—	614	896
Corollosporella ramulosa	PP1232	*Varicosporina ramulosa*	—	—	AF491276	—	—	867
Corollosporella ramulosa	RVG-113	*Varicosporina ramulosa*	U43846	—	U44092	1000	—	878
Corollosporella ramulosa	NBRC 32163	*Varicosporina ramulosa*	—	—	NBRC32163	—	—	563
Corollosporella ramulosa (see notes)	*NBRC 31325	*Varicosporina ramulosa*	—	AB361033 (d)	AB361020	—	571	1298
Cucullosporela mangrovei	NBRC 32477	*Cucullosporela mangrovei*	—	NBRC 32477	NBRC 32477	—	1329	1329
Cucullosporela mangrovei	NBRC 105272	*Cucullosporela mangrovei*	—	NBRC 105272	NBRC 105272	—	785	562
Cucullosporela mangrovei	NTOU3744	*Cucullosporela mangrovei*	KX686771	—	KX686772	964	—	963
Trailia ascophylli	NBRC 106638	*Trailia ascophylli*	—	NBRC 106638	NBRC 106638	—	597	566
Trailia ascophylli	NBRC 106637	*Trailia ascophylli*	—	NBRC 106637	NBRC 106637	—	598	566
Ascosacculus fusiformis	MELUCC 14-0036	*Ascosacculus fusiformis*	NG_070127	MK878373	NG_070127			—
	MELU 15-0068	*Ascosacculus fusiformis*	—	NR_168817	—	—		—
Ascosacculus heteroguttulatus	A108-7D	*Ascosacculus heteroguttulatus*	AF352085	—	AF352085		—	—
Carbosphaerella	NBRC 106647	*Carbosphaerella leptosphaerioides*	NBRC106647	NBRC106647	—			—

leptosphaerioides	NBRC 106648	*Carbosphaerella leptosphaerioides*	NBRC106648	NBRC106648	—			—
Cucurbitinus constrictus	CGMCC3.19606	*Cucurbitinus constrictus*	MN431419	MN431306	MN437320	822	629	1438
	CGMCC3.19640	*Cucurbitinus constrictus*	MN431420	MN431307	MN437321	801	613	1026
Halosphaeriopsis mediosetigera/Trichocladium achrasporum	NBRC 32127	*Halosphaeriopsis mediosetigera*	NBRC32127	NBRC32127	NBRC32127	547	593	1751
	NBRC 32128	*Halosphaeriopsis mediosetigera*	NBRC32128	NBRC32128	NBRC32128	559	594	1751
	NBRC 32161	*Trichocladium achrasporum*	NBRC32161	NBRC32161	NBRC32161	1195	1195	—
	NBRC 105258	*Trichocladium achrasporum*	NBRC105258	NBRC105258	NBRC105258	559	586	—
Oceanitis scuticella	M0229768	*Oceanitis scuticella*	KU712509	KU712506	—	992	436	—
	CP2421	*Oceanitis scuticella*	EU118367	KU712507	—	993	437	—
	CP2429	*Oceanitis scuticella*	EU118366	KU712508	—	987	436	—
Phaeonectriella lignicola	PP7008	*Phaeonectriella lignicola*	AY150224	AY150224	—	1774	1774	—
Tinhaudeus formosanus	NTOU3805	*Tinhaudeus formosanus*	KT159899	KT159895	KT159897	961	599	964
Microascus longirostris	CBS 415.64	*Microascus longirostris*	LM652516	LM652422	—	584	533	—
	*CBS 196.61	*Microascus longirostris*	NG_058479	NR_132945	—	784	540	—
Microascus cirrosus	*CBS 217.31	*Microascus cirrosus*	MH866642	MH855194	—	899	590	—
Petriella sordida	CBS 144612	*Petriella sordida*	MK442544	MK442608	—	794	565	—
	CBS 145121	*Petriella sordida*	MK442545	MK442609	—	791	576	—
Xylaria hypoxylon	ATCC_42768	*Xylaria hypoxylon*	AY327480	AY327477	—	573	963	—
	AFTOL-ID 51	*Xylaria hypoxylon*	AY544648	DQ491487	—	1373	573	—
