# Supplementary material for: Redefining the Genus Corollospora Based on Morphological and Phylogenetic Approaches
Source: J Fungi (Basel). 2023 Aug 11;9(8):841. doi: 10.3390/jof9080841 (PMC10455288; doi:10.3390/jof9080841)
Supplement: Supplementary file 1 [file jof-09-00841-s001.zip › Table S2 (Notes to Table 2 and Table S1).pdf]

**TAXON**<br>**Current name**	**Table S2 (**Notes to Table 2 and to Table S1)<br>(according to the data provided in the Supplementary Files 1, 2)
Corollospora maritima (*C. maritima sensu stricto)*<br>**(Group 1)**	Consisting of 104 strains, all represented by ITS sequences and only 21 by 28S sequences. *C. maritima* NBRC 106651 (labeled ● in Table 2) was evaluated together. In the ITS region, the strain MD 825 strongly reduced the MSA length and was not used. In what concerns the p-distances found, detailed explanations are given in section 3.4.1.
Corollospora maritima* + *Corollospora portsaidica<br>**(Group 2)**	Consisting of 60 strains, three registered as *C. portsaidica* and 57 as *C. maritima*; 56 strains (two *C. portsaidica*) are represented by ITS sequences and three strains (two *C. portsaidica*) by 28S sequences. Only one strain (*C. maritima* NBRC 32118) was represented by ITS, 28S and 18S sequences. *C. maritima* NBRC 106651 (labeled ● in Table 2) was evaluated together. In what concerns p-distances found, detailed explanations are given in section 3.4.1.
Corollospora maritima<br>**(Group 3)**	Consisting of eleven isolates with only ITS sequences. They segregated in two sub-groups at a p-distance of 4% from each other and of 0% between the sequences within each group. One sub-group has nine sequences from samples collected in Hawaii, and the other has two sequences of samples from Cuba. *C. maritima* NBRC 106651 (labeled ● in Table 2), evaluated together, segregated by a p-distance of 16% from both sub-groups.
Corollospora mediterranea	Most isolates share p-distances of 0%; only two isolates (MUT 5049 and MUT 1954) showed p-distances of 1% to the others in the ITS region, as well as between themselves, in the 28S gene. Additional note: the 18S sequence of the isolate MUT 5082 has 1027/1027 identities (100%) with 18S sequences of *Paralulworthia elbensis*, in accordance with the p-distance of 7% evidenced to the other eight isolates of *C. mediterranea*.
Corollospora quinqueseptata	No available ITS sequences. In the 28S gene, the strains NBRC 32114 and NBRC 32115 (type strain) segregate by a p-distance of 0% and by 8% from the isolate PP3909 (member of a new genus?).
Corollospora anglusa	The holotype of *C. anglusa* is the strain MF 827 [= NBRC 104919, =MD 827 (holotype specimen, in NCBI)]. The ITS and 28S sequences retrieved from NBRC were used instead of the NCBI sequences because the ITS sequence from NCBI (AB361022) mostly covers the 28S region (82%) without showing the expected 100% identity with its own 28S sequenc (AB361008); however, as expected, the 28S sequences from both origins are identical (p-distance of 0%).
Corollospora cinnamomea	Represented by four strains (none is type strain) with 28S sequences, and one of them (NBRC 32125) with a short ITS sequence (471 nt but also partially covering the 18S region). In the ITS region (MSA with 414 positions) this strain segregates from *C. maritima sensu stricto*, by a p-distance of 14%. Due to length limitations, the ITS sequence could not be subjected to the global alignment.
Corollospora gracilis	In the ITS region, 17 members segregate by a maximum p-distances of 2%, and by 3–4% from the strain MD 828. Three sub-groups of sequences clustering at p-distances of 0%: 1) strains 9 E08 and 23; 2) strains SFC102400 and NBRC 32111; 3) the other 13 strains. The type strain NBRC 32110 could not be assessed for the ITS region due to its short length (206 nt, only covering the ITS 2 region) but in a short MSA (204 nt) it evidenced a greater proximity with the strains SFC102400 and NBRC 32111. Concerning the 28S gene (only three strains), they segregated by p-distances of 0%, also including the strain MD 828.

Corollospora parvula	The isolate IFO32159 (=NBRC 32159) was originally identified as *Sigmoidea marina* and later reassigned to *Sigmoideia parvula* (Jones et al. 2009). However, this reclassification was not updated in the NCBI database where its sequences still are registered as *Sigmoidea marina* .
Varicosporina ramulosa	Only one ITS sequence available (from the type strain CBS 398.65) because other ITS sequence (NBRC 31325) was rejected considering that it does not match with any sequence from this region and evidences (the best hit by BLAST) 94% identity with the 28S sequence from the type strain. Moreover, both strains have 28S sequences clustering with other three strains at p-distances of 0%.
Corollospora pseudopulchella	Only the type strain NBRC 32112 has an ITS sequence, retrieved from UNITE. For the 28S gene, the four strains NBRC segregated in two sub-groups, with two sequences each (NBRC 32112/32113 and NBRC 106641/106642); p-distances of 0% were found within each sub-group and of 8% between the two sub-groups, evidencing that they comprise distinct species. According to the values of p-distance to *C. maritima sensu stricto* (15-16% in the ITS region and 5% or 8% in the 28S gene), these sub-groups may correspond to new genera.
Corollospora angusta	In the ITS region, the 18 strains segregate by a p-distance of 0–1%. In the 28S gene, 17 segregate by p-distances of 0%, exception for a strain without ITS sequence (AFTOL-ID 5010) at p-distance of 1% from the others. This strain was not included in the global evaluations, considering the absence of representation in the ITS region.
Corollospora luteola* + *Corollospora marina	*Corollospora luteola and Corollospora marina* (=*Halosigmoidea marina,* =*Sigmoidea marina*) were evaluated together because their proximity was previously recognized by Jones et al. (2009; [61]). However, only one isolate (*Sigmoidea marina* NBRC 103271) could be considered in the evaluations involving the ITS region due to the short length (381 nt) of the sequence from *Halosigmoidea luteola* TUB 557, and absence of published sequences of *C. luteola.* In the 28S gene, the p-distances depended on the length of the MSA: a short MSA evidenced p-distances of 0% between six strains under analysis (three *C. luteola* + three *Halosigmoidea marina*) and 1% between them and *Sigmoidea marina* AFTOL ID 5008; a larger MSA (without *Sigmoidea marina* TUB 2) evidenced p-distances of 0% within each species and 1% between the two species.
Corollospora colossa	Two strains with ITS and 28S sequences subjected to the global assessments, as well as the type strain NBRC 32103 without ITS sequence. The strain PP3904 was not considered because it only has a short ITS sequence (167 nt) and the 28S sequence evidences a p-distance of 5–12%, from the others. The type strain, yet not represented in the ITS region, was considered for the global evaluation due to its clear segregation, either from the other two strains (p-distance of 7–8%), or from *C. maritima sensu stricto* (11%). Additional note: the type strain has a problematic 18S sequence: 100% identity with the 18S sequences of *C. pulchella* NBRC 32124 and *C. fusc* a NBRC 32108 (1094/1094 identities with both).
Corollospora filiformis	Taxon with two type strains originated from the same organism; only one (CBS 125146) has an ITS sequence. Other four strains (including the type strain: NBRC 32106) also have 28S sequences at a p-distance of 0–1% to each other and 2% to the strain CBS 125146. This conflicting result indicates that the molecular data needs re-evaluation.

Corollospora fusca	The strain PP3906 was not evaluated because no significant correspondence was found between its single 28S sequence and any *Corollospora*. Two strains (NBRC 32107/32108) evidenced p-distances of 0% between them and 5% / 1%, from the strain NBRC 32109, in the ITS / 28S regions. Additional note: the 18S sequence of the strain NBRC 32108 is a problematic sequence since it has 100% identity with *C. colossa* NBRC 32103 and *C. pulchella* NBRC 32124 (1094/1094 identities with both).
Corollospora lacera	ITS sequences not available and 28S sequences not attributed to any type strain. However, very clear results were obtained in the 28S assessment: two strains (NBRC 32121/32122) segregated from each other by a p-distance of 0%, and 7% from the strain PP2509, which appears to belong to a differnt species.
Corollospora pulchella* + *Clavariopsis bulbosa	Only *Clavariopsis bulbosa* NBRC 31323 (type strain) and *C. pulchella* NBRC 32123 have ITS and 28S sequences. The strains PP4294 and PP4206 were not considered because they are only represented by 28S sequences with low identity (≤90%) with the other strains of *C. pulchella*. For the 28S gene, the 13 strains evaluated segregate in two groups (9+4 sequences) by p-distances of 0–1% within each group and 3% between the groups. Additional note: the single 18S sequence from *C. pulchella* NBRC 32124 is problematic: 100% identity with the 18S sequences from *C. colossa* NBRC 32103 and *C. fusca* NBRC 32108 (1094/1094 identities with both).
***Corollospora intermedia* + *Varicosporina prolifera* /** Inconclusive placement due to contraditory results	*Corollospora intermedia* and *Varicosporina prolifera* were evaluated together because they were recognized as two morphs of the same species by Nakagiri (1986; [59]). Only the strains *C. intermedia* NBRC 104402 and *Varicosporina prolifera* NBRC 32120 could be compared in the ITS region (p-distance of 11%) because an unnamed strain has a very short sequence (375 nt). They also segregate from each other by a high p-distance (4%) in the 28S gene, where both segregate by much higher distances (12% and 15%, respectively) from another member: *C. intermedia* PP3910.
